# Supplementary material for: A unique symbiosome in an anaerobic single-celled eukaryote
Source: Nat Commun. 2024 Nov 9;15:9726. doi: 10.1038/s41467-024-54102-7 (PMC11550330; doi:10.1038/s41467-024-54102-7)
Supplement: Supplementary file 1 — Supplementary Information [file 41467_2024_54102_MOESM1_ESM.pdf]

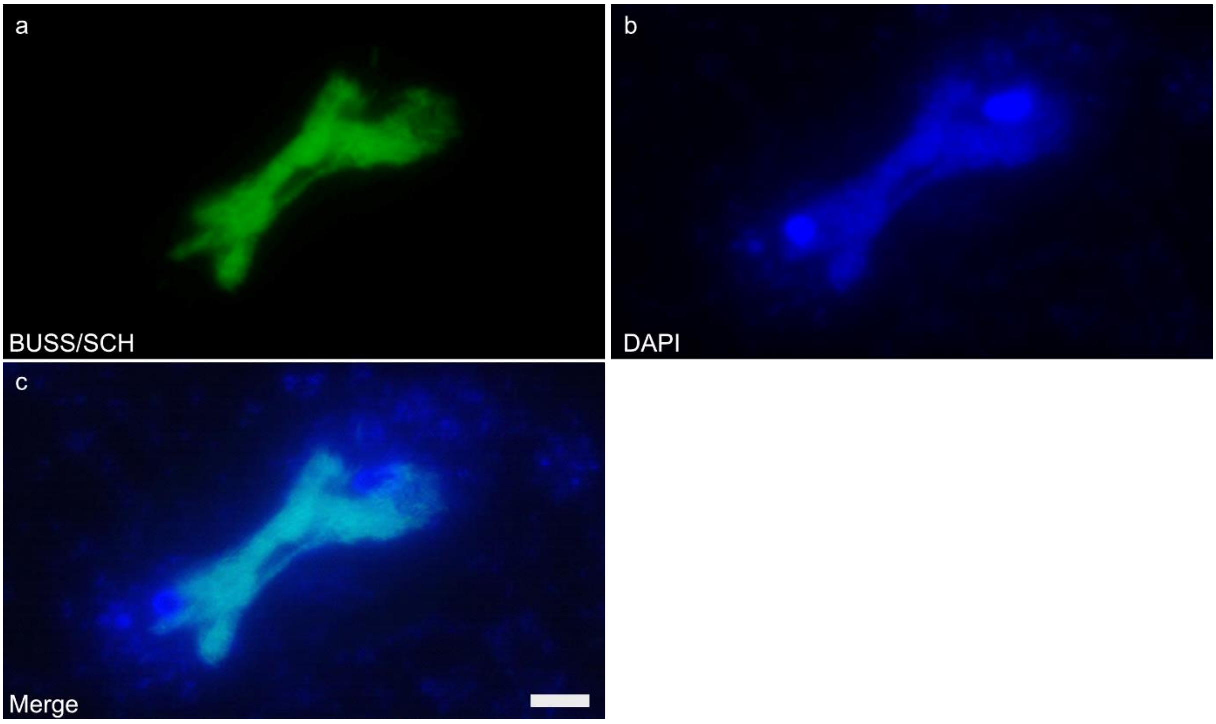

Figure S1: *A. flamelloides* BUSSELTON2 symbionts segregating during cytokinesis. *A. flamelloides* BUSSELTON2 hybridized with **a,** probe BUSS/SCH-BMN-488 and stained with **b,** DAPI. **c,** merged image of **a,** and **b,**. Scale bar 5 µm.

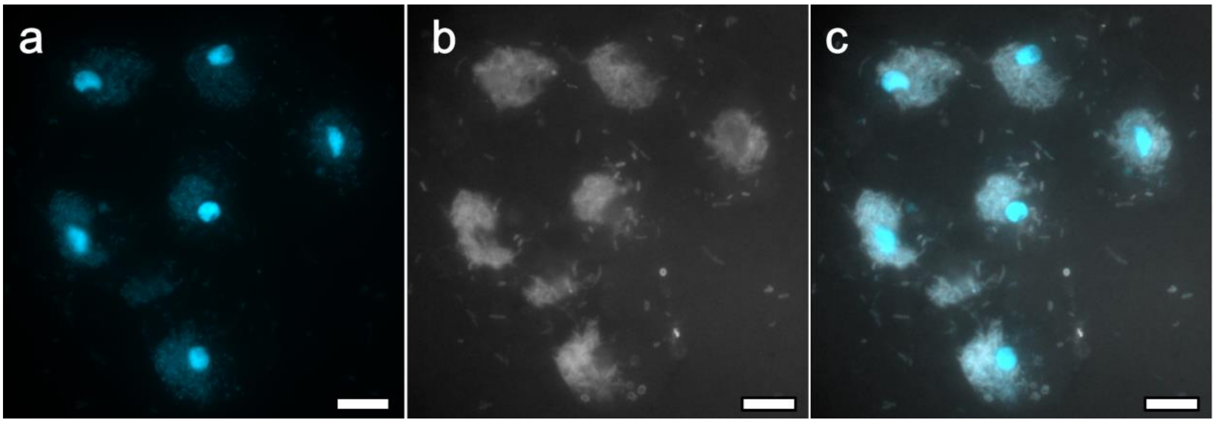

Figure S2: Wheat germ agglutinin (WGA) staining of *Anaeramoeba* and symbionts. *A. flamelloides* BUSSELTON2 cells were live-stained for 10 min using WGA-CF633, washed using artificial sea water and fixed in 4% formaldehyde. The cells were mounted in DAPI containing mounting media and were imaged using wide-field microscopy. The surface of the symbiont is stained by the WGA lectin at a similar intensity as free-living bacteria. **a,** *A. flamelloides* BUSSELTON2 stained with DAPI. **b,** WGA-CF633 staining, **c,** merged image of **a,** and **b,**. Scale bar 10 µm.

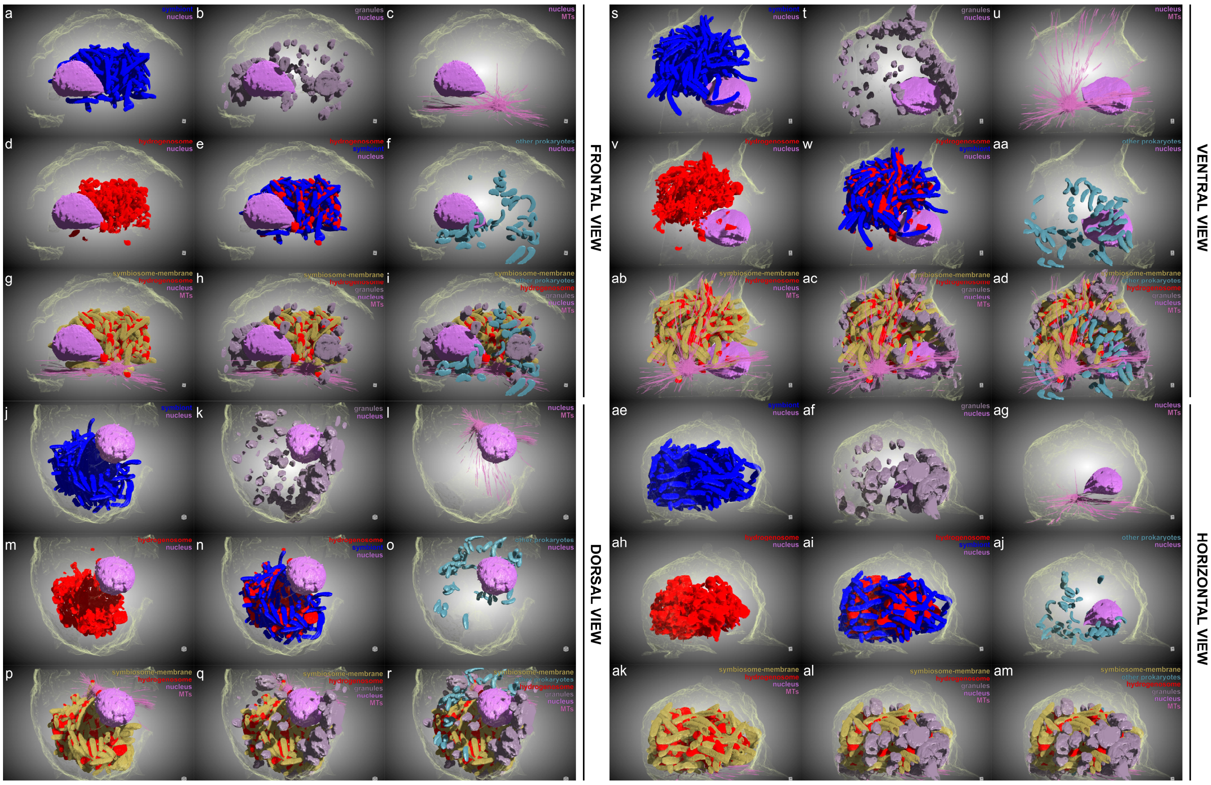

Figure S3: Segmentations of *Anaeramoeba* cell features. 3D reconstructions of segmented cell structures from FIB-SEM of *A. flamelloides* BUSSELTON2. **a-i,** frontal view. **j-r,** dorsal view. **s-ad,** ventral view. **ae-am,** horizontal view. The outline of the plasma membrane (shaded yellow) and nucleus (purple) are shown in each view. Depending on the panel, segmented regions of interest (symbiont – blue, hydrogenosome – red, symbiosome-membrane – gold, granules – aubergine, other prokaryotes – blue green, microtubules (MTs) - pink) are displayed as indicated on each respective legend.

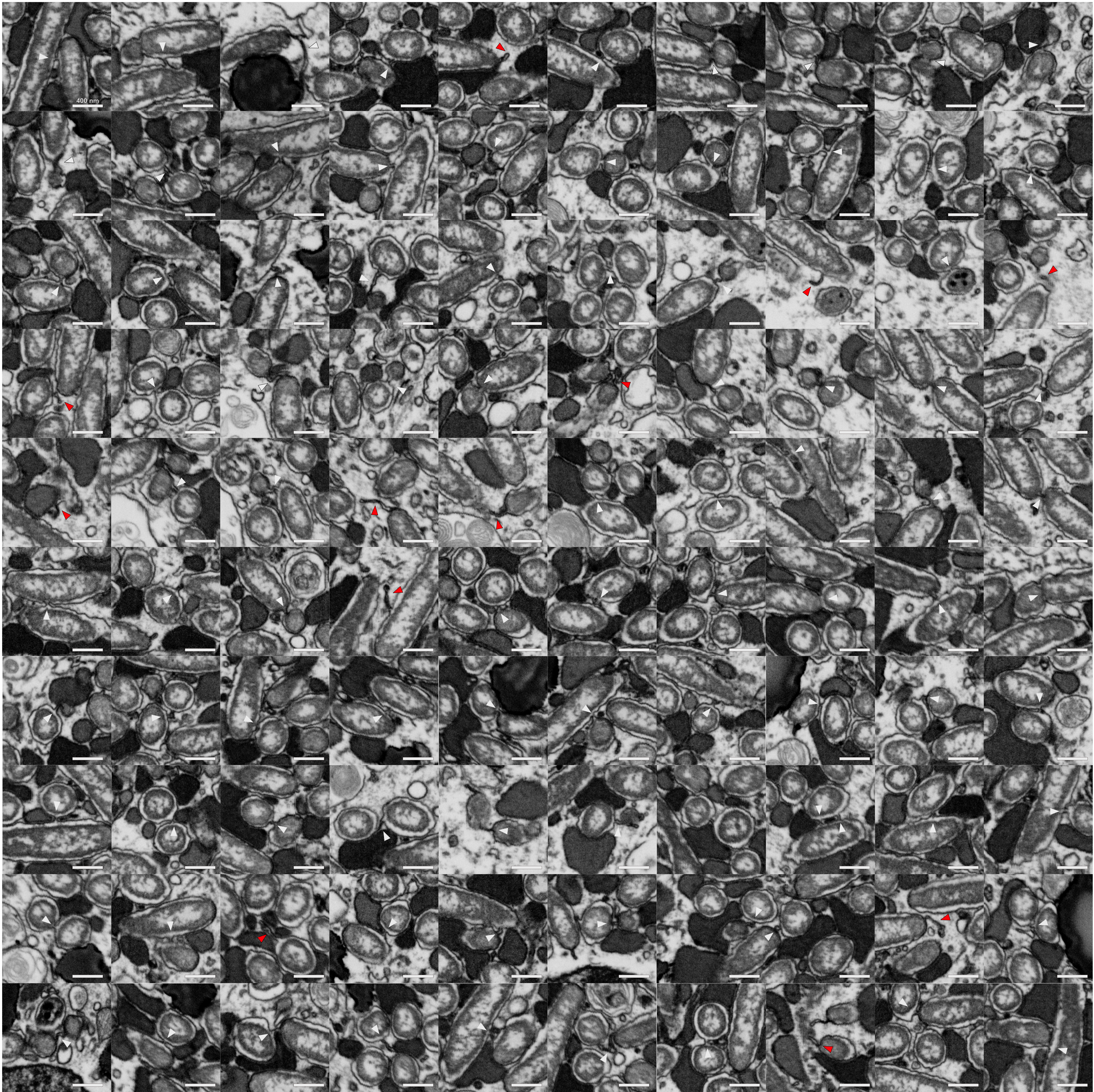
400 nm

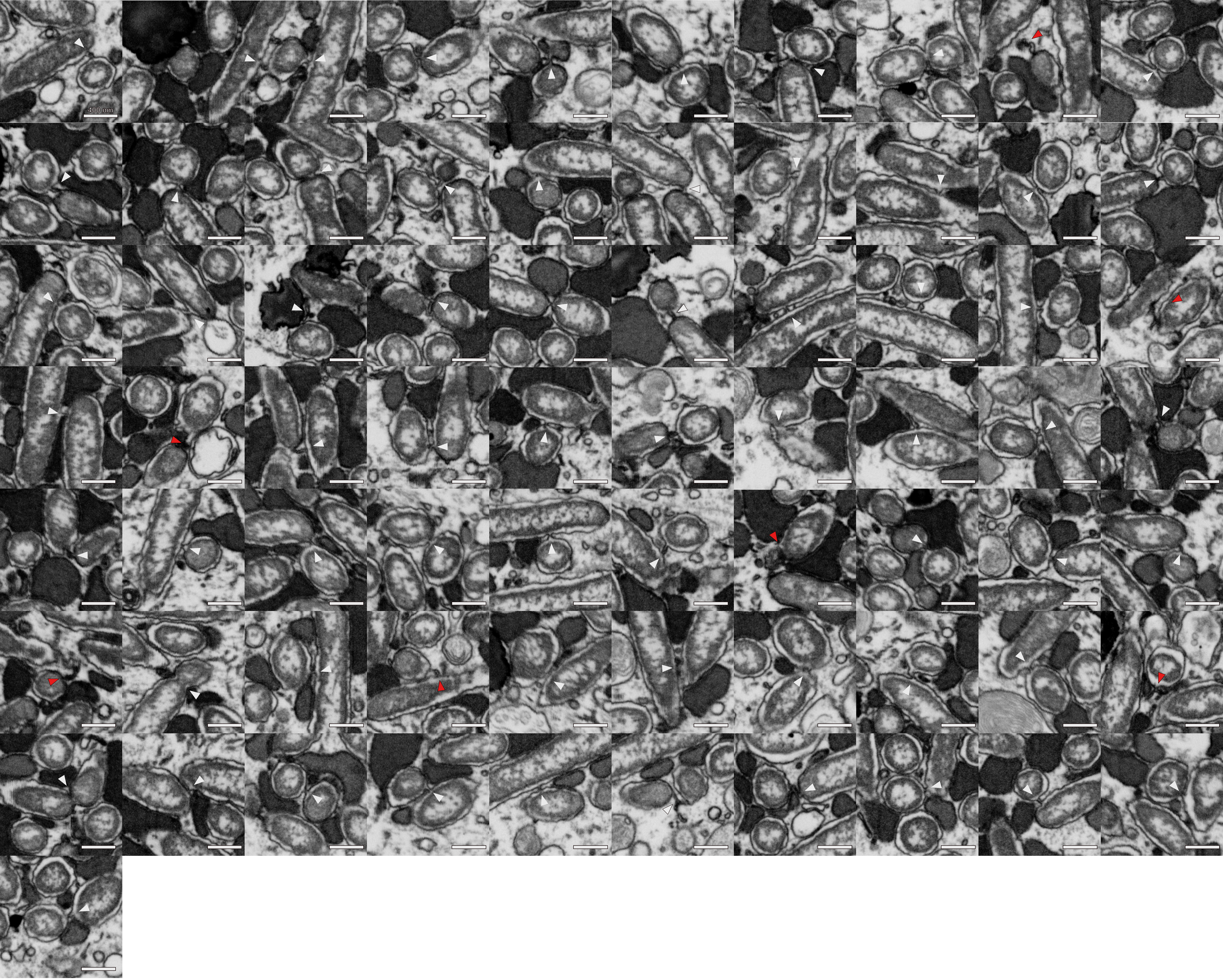
400 nm

Figure S4: Symbiosome-membrane contacts. Extracted regions of interest from slices in the *A. flamelloides* BUSSELTON2 FIB-SEM stack showing 171 examples of symbiosome-membrane contact sites. White arrowheads display a cell-to-cell connection traceable in a single slice. Red arrowheads indicate a connection that is traceable across several slices. Scale bars 400 nm.

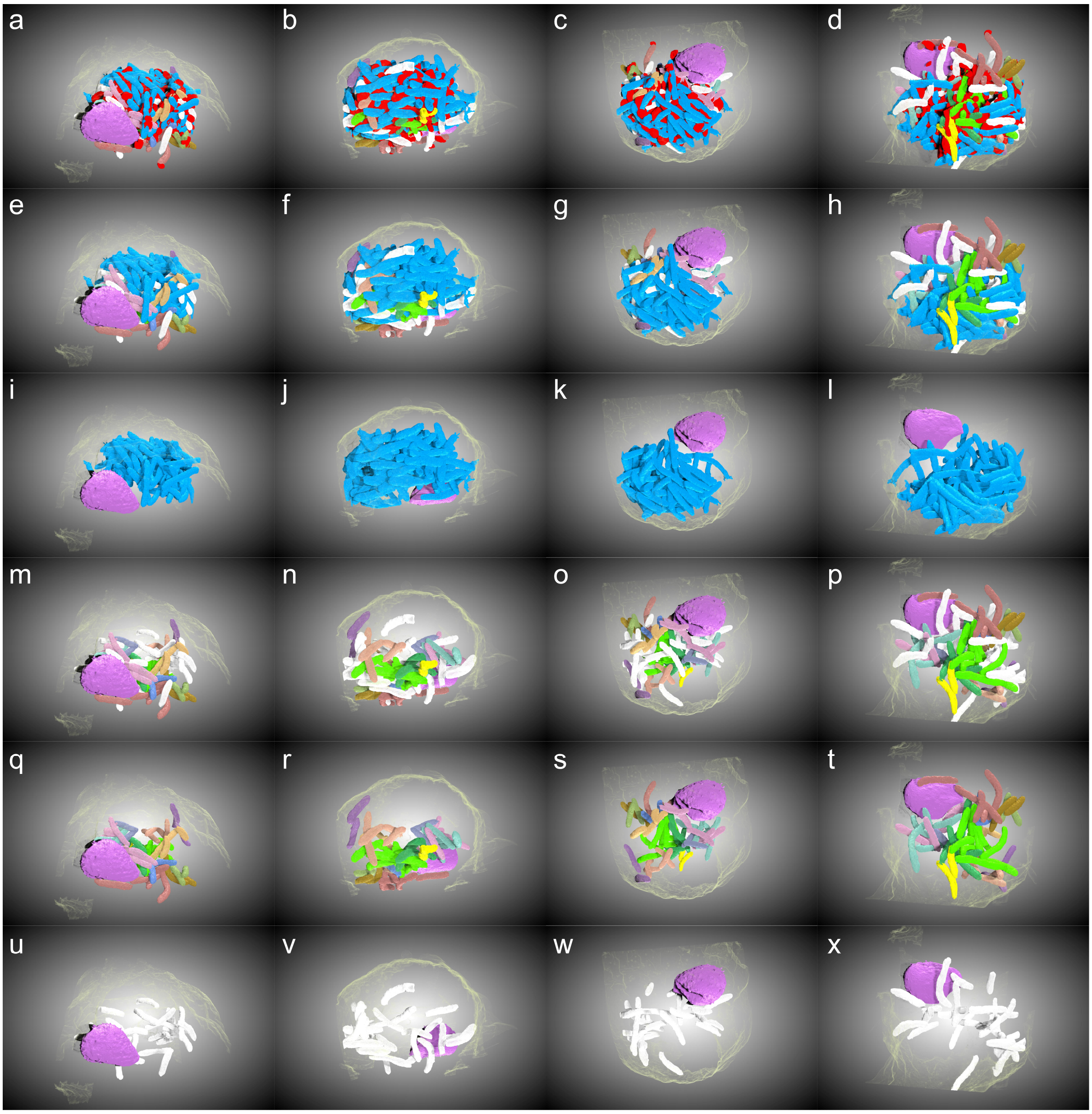

FRONTAL VIEW | HORIZONTAL VIEW | DORSAL VIEW | VENTRAL VIEW

Figure S5: Segmentations of *Anaeramoeba* connected symbiosome components. 3D reconstructions of connected symbiosome components from FIB-SEM of *A. flamelloides* BUSSELTON2. **a-d,** all symbiosome components, hydrogenosomes. **e-h,** all symbiosome components. **i-l,** symbiosome components connected to the outside media. **m-p,** symbiosome components not showing connections to the outside. **q-t,** symbiosome compartments with two or more connected components. **u-x,** symbiosome compartments that are isolated. **a, e, l, m, q, u,** frontal view. **b, f, j, n, r, v,** horizontal view **c, g, k, o, s, w,** dorsal view. **d, h, l, p, t, x,** ventral view. The outline of the plasma membrane (shaded yellow) and nucleus (purple) are shown in each view.

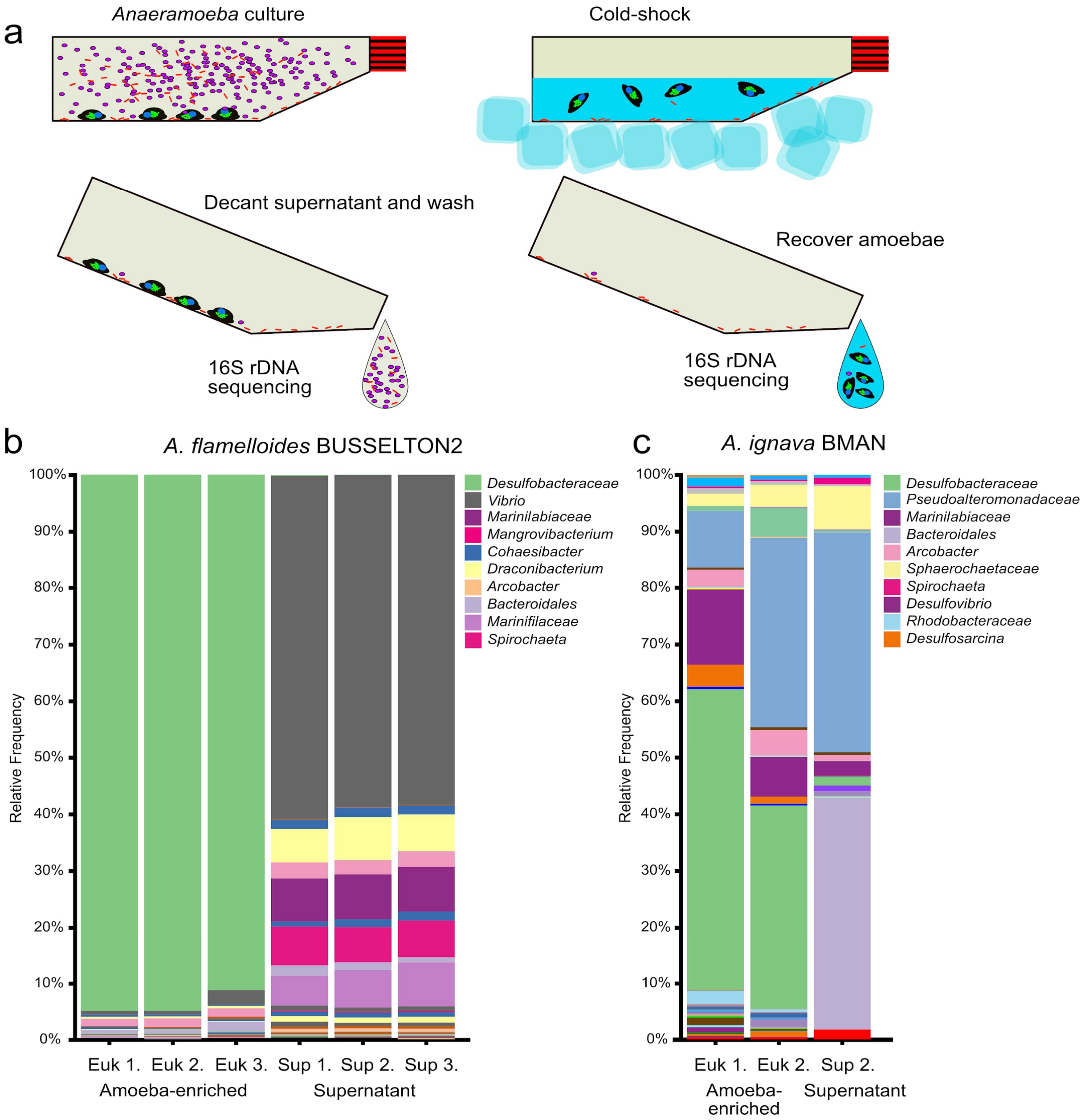

Figure S6: Amoebae enrichment procedure and 16S rDNA sequencing. a, The amoebae are enriched from xenic cultures by resuspending planktonic bacteria and decanting the supernatant followed by several washes using artificial sea water. The amoebae are selectively enriched from adherent bacteria using cold-shock detachment and differential centrifugation. The efficiency of enrichment is monitored by 16S rDNA sequencing. **b,** The relative frequency of bacterial taxa based on the V4 region of 16S rDNA analysis from triplicate amoeba-enriched and supernatant fractions of *A. flamelloides* BUSSELTON2. **c,** The relative frequency of bacterial taxa based on analysis of the V4 region of 16S rDNA from two amoeba-enriched and one supernatant fraction of *A. ignava* BMAN. The enrichments in **b,** and **c,** were prepared using the method described in **a,**.

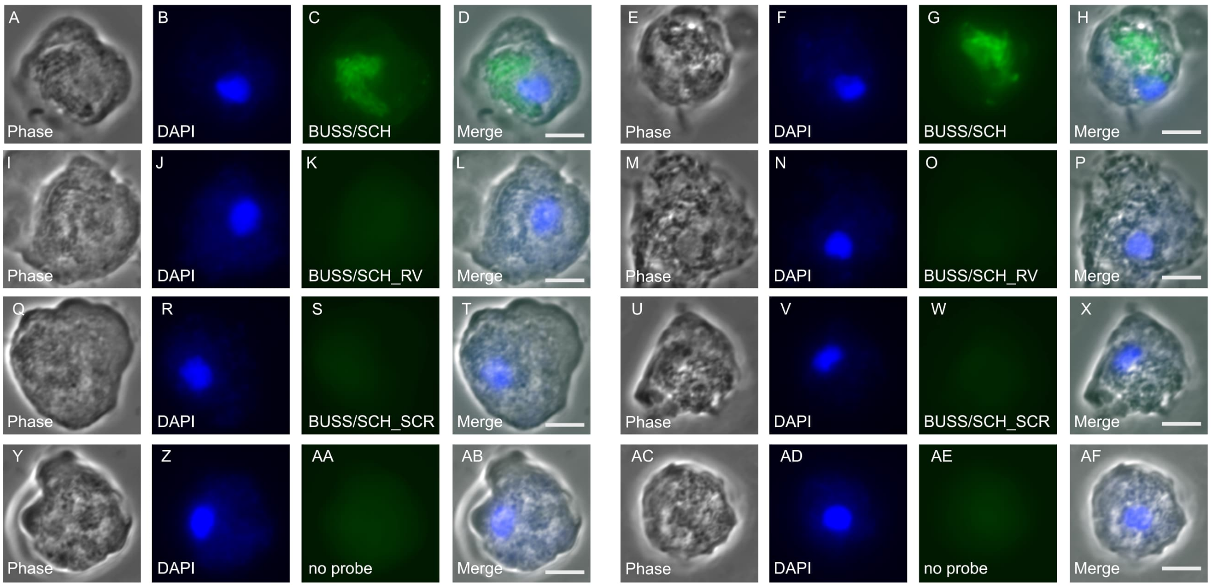

Figure S7: Specificity testing of BUSS/SCH FISH probe in *A. flamelloides* BUSSELTON2/SCHOONER1. The BUSS/SCH probe hybridize to *A. flamelloides* BUSSELTON2/SCHOONER1 symbionts whereas the reverse complement probe BUSS/SCH_RV and the scrambled probe BUSS/SCH_SCR show background similar to no probe control hybridizations. **a-d,** *A. flamelloides* SCHOONER1, **a,** phase contrast of amoeba stained with **b,** DAPI and hybridized with **c,** probe BUSS/SCH-BMN-488, **d,** merged **a-c,**. **e-h,** *A. flamelloides* BUSSELTON2, **e,** phase contrast of amoeba stained with **f,** DAPI and hybridized with **g,** probe BUSS/SCH-BMN-488, **h,** merged **e-g,**. **i-l,** *A. flamelloides* SCHOONER1, **i,** phase contrast of amoeba stained with **j,** DAPI and hybridized with **k,** probe BUSS/SCH_RV-BMN-488, **l,** merged **i-k,**. **m-p,** *A. flamelloides* BUSSELTON2, **m,** phase contrast of amoeba stained with **n,** DAPI and hybridized with **o,** probe BUSS/SCH_RV-BMN-488, **p,** merged **m-o,**. **q-t,** *A. flamelloides* SCHOONER1, **q,** phase contrast of amoeba stained with **r,** DAPI and hybridized with **s,** probe BUSS/SCH_SCR-BMN-488, **t,** merged **q-s,**. **u-x,** *A. flamelloides* BUSSELTON2, **u,** phase contrast of amoeba stained with **v,** DAPI and hybridized with **w,** probe BUSS/SCH_SCR-BMN-488, **x,** merged **u-w,**. **y-ab,** *A. flamelloides* SCHOONER1, **y,** phase contrast of amoeba stained with **z,** DAPI and hybridized with **aa,** no probe, **ab,** merged **y-aa,**. **ac-af,** *A. flamelloides* BUSSELTON2, **ac,** phase contrast of amoeba stained with **ad,** DAPI and hybridized with **ae,** no probe, **af,** merged **ac-ae,**. Scale bar 10 µm.

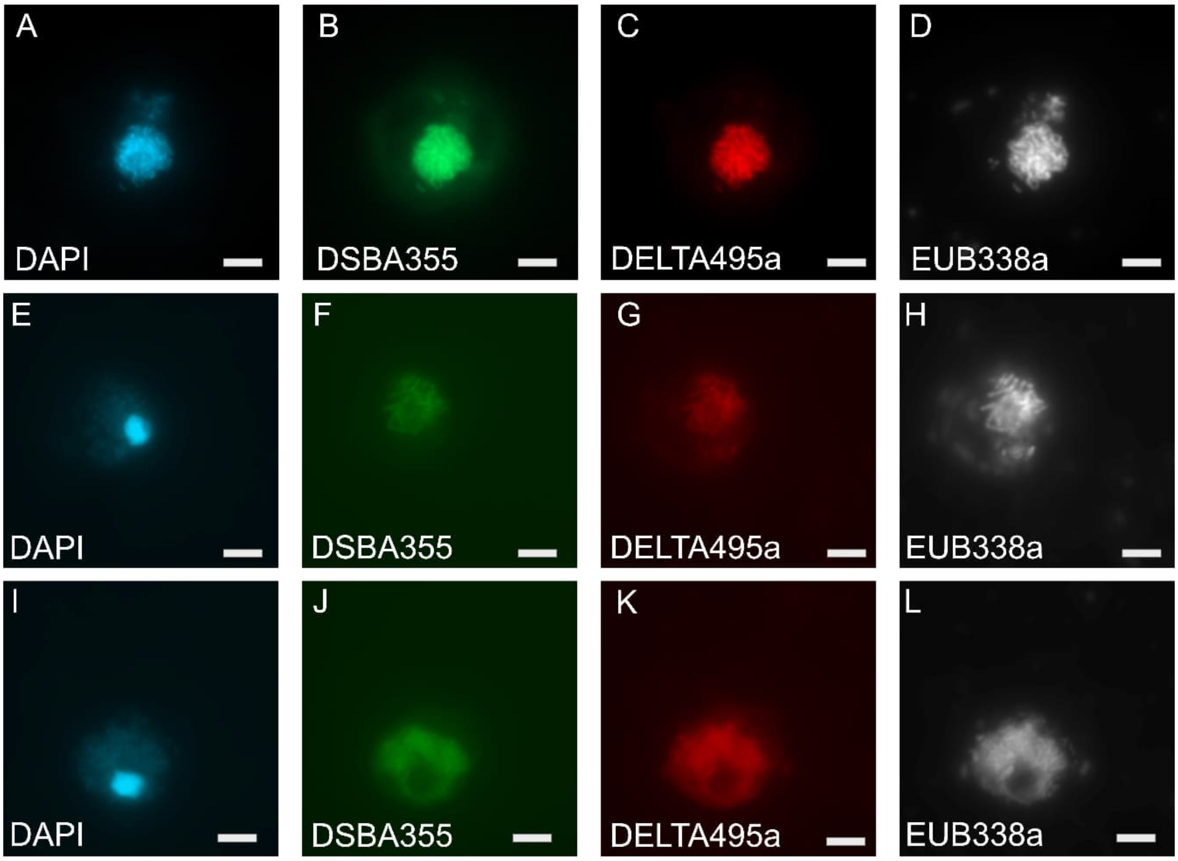

Figure S8: Fluorescence in situ hybridization (FISH) of the symbionts in *A. ignava* BMAN and *A. flamelloides* BUSSELTON2/SCHOONER1. a-d, *A. ignava* BMAN stained with **a,** DAPI and hybridized with **b,** probe DSBA355-BMN-488, **c,** probe Delta495a-Atto 550 and **d,** probe EUB338a-Atto 633. **e-h,** *A. flamelloides* BUSSELTON2 stained with **e,** DAPI and hybridized with **f,** probe DSBA355-BMN-488, **g,** probe Delta495a-Atto 550 and **h,** probe EUB338a-Atto 633. **i-l,** *A. flamelloides* SCHOONER1 stained with **i,** DAPI and hybridized with **j,** probe DSBA355-BMN-488, **k,** probe Delta495a-Atto 550 and **l,** probe EUB338a-Atto 633. Scale bar 5 µm.

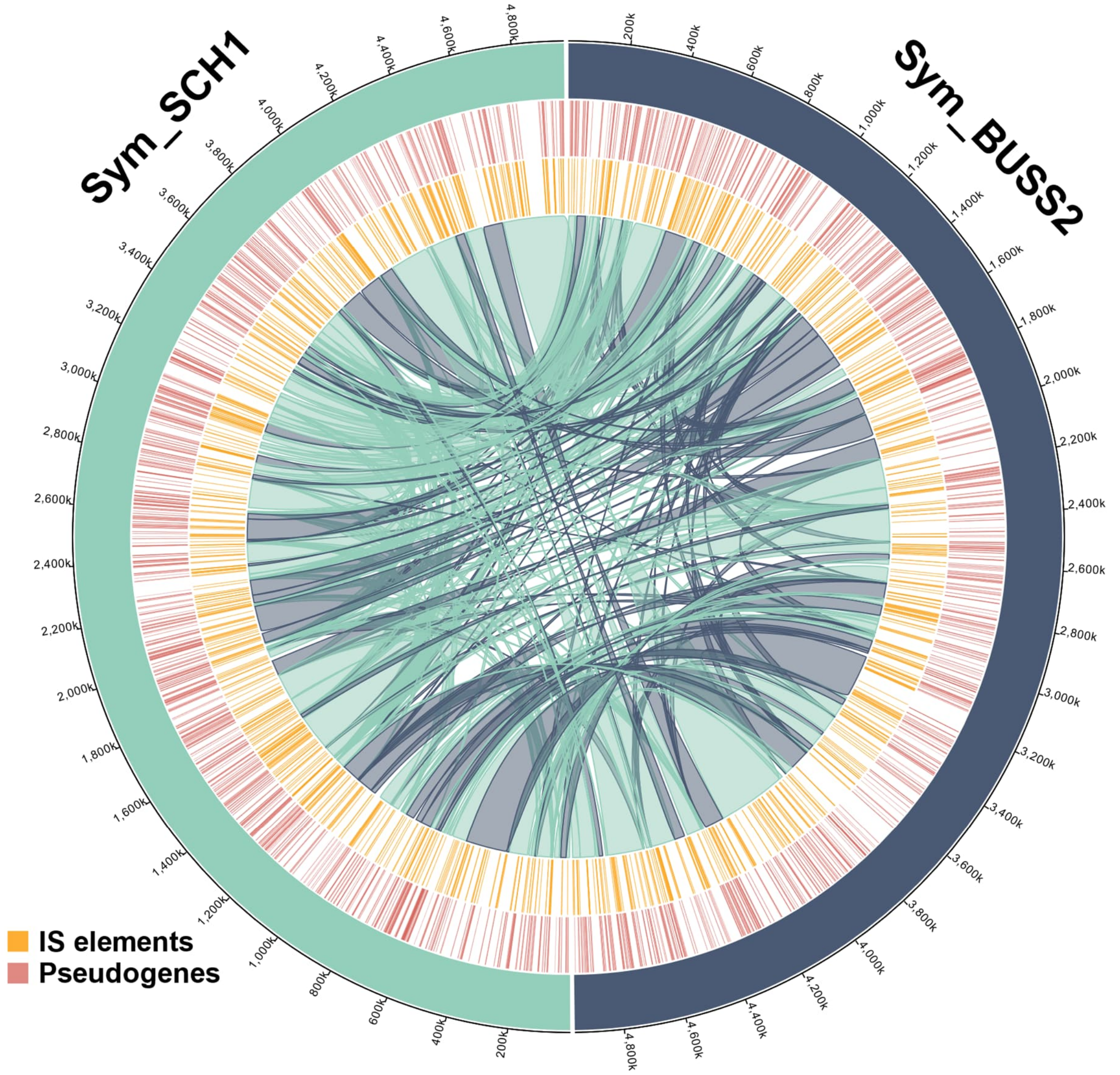

Figure S9: Extensive synteny differences between Sym_BUSS2 and Sym_SCH1. The synteny of the Sym_BUSS2 genome (4,958,664 bp) and the largest contig of the Sym_SCH1 genome (4,969,409 bp) were determined using Sibelia [1] and visualized as a ribbon-plot (dark blue – sense segment, cyan – inverted segment). The inner track (yellow lines) shows IS elements predicted using ISsaga 2.0 (http://issaga.biotoul.fr/). The middle track (red lines) shows the positions of pseudogenes. The outer track displays the respective genomes (cyan – Sym_SCH1, dark blue – Sym_BUSS2). Genome coordinates are indicated by outer ticks every 200 kbp. The figure was prepared using Circa (OMGenomics).

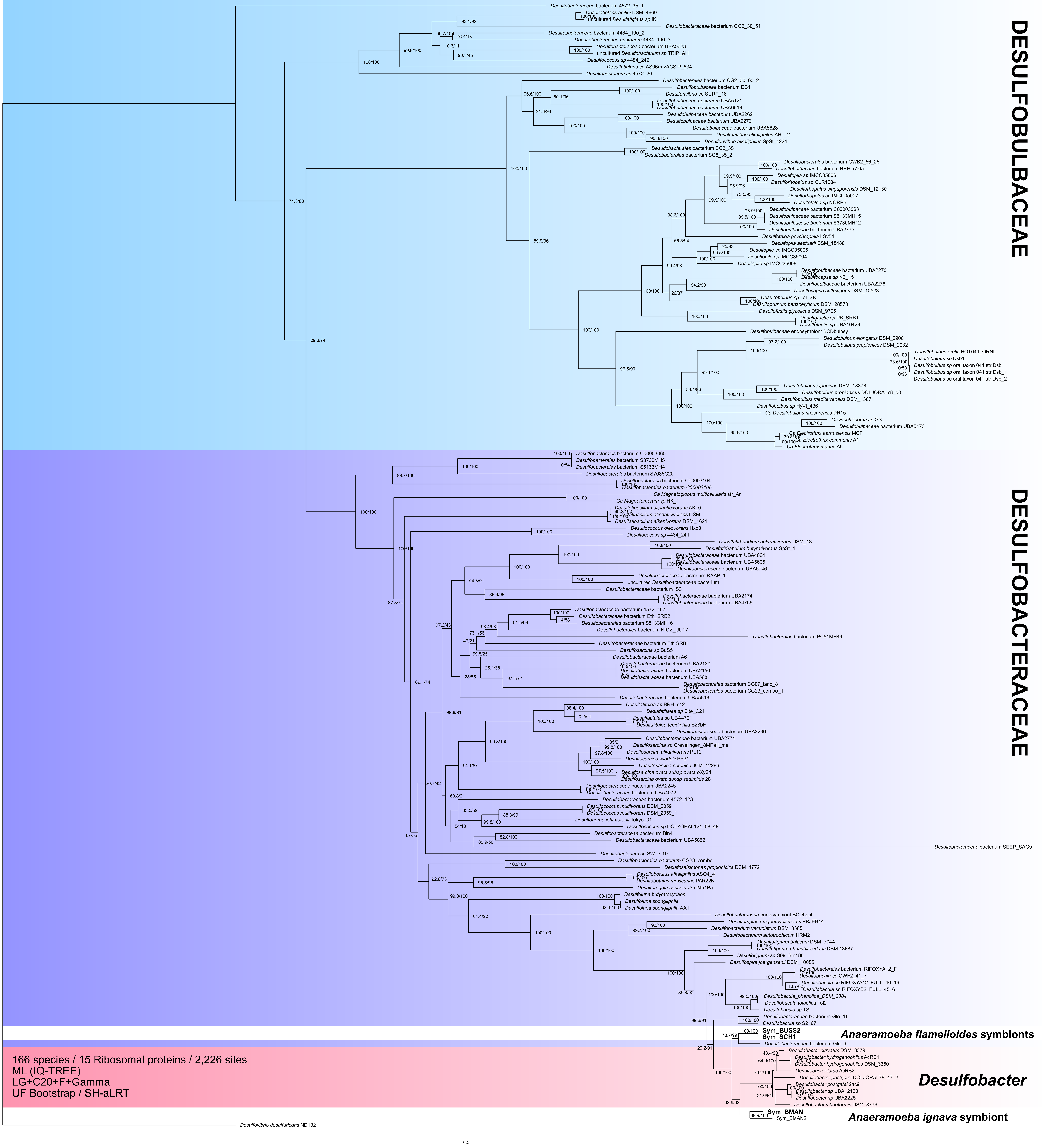

DESULFOBULBACEAE
DESULFOBACTERACEAE
Anaeramoeba flamelloides symbionts
Desulfobacter
Anaeramoeba ignava symbiont
166 species / 15 Ribosomal proteins / 2,226 sites
ML (IQ-TREE)
LG+C20+F+Gamma
UF Bootstrap / SH-aLRT
Desulfobacteraceae bacterium 4572_35_1
Desulfatiglans anilini DSM_4660
uncultured Desulfatiglans sp IK1
Desulfobacteraceae bacterium CG2_30_51
Desulfobacteraceae bacterium 4484_190_2
Desulfobacteraceae bacterium 4484_190_3
Desulfobacteraceae bacterium UBA5623
uncultured Desulfobacterium sp TRIP_AH
Desulfococcus sp 4484_242
Desulfatiglans sp AS06rmzACSIP_634
Desulfobacterium sp 4572_20
Desulfobacterales bacterium CG2_30_60_2
Desulfobulbaceae bacterium DB1
Desulfurivibrio sp SURF_16
Desulfobulbaceae bacterium UBA5121
Desulfobulbaceae bacterium UBA6913
Desulfobulbaceae bacterium UBA2262
Desulfobulbaceae bacterium UBA2273
Desulfobulbaceae bacterium UBA5628
Desulfurivibrio alkaliphilus AHT_2
Desulfurivibrio alkaliphilus SpSt_1224
Desulfobacterales bacterium SG8_35
Desulfobacterales bacterium SG8_35_2
Desulfobacterales bacterium GWB2_56_26
Desulfobulbaceae bacterium BRH_c16a
Desulfopila sp IMCC35006
Desulforhopalus sp GLR1684
Desulforhopalus singaporensis DSM_12130
Desulforhopalus sp IMCC35007
Desulfotalea sp NORP6
Desulfobulbaceae bacterium C00003063
Desulfobulbaceae bacterium S5133MH15
Desulfobulbaceae bacterium S3730MH12
Desulfobulbaceae bacterium UBA2775
Desulfotalea psychrophila LSv54
Desulfopila aestuarii DSM_18488
Desulfopila sp IMCC35005
Desulfopila sp IMCC35004
Desulfopila sp IMCC35008
Desulfobulbaceae bacterium UBA2270
Desulfocapsa sp N3_15
Desulfobulbaceae bacterium UBA2276
Desulfocapsa sulfexigens DSM_10523
Desulfobulbus sp Tol_SR
Desulfoprunum benzoelyticum DSM_28570
Desulfofustis glycolicus DSM_9705
Desulfofustis sp PB_SRB1
Desulfofustis sp UBA10423
Desulfobulbaceae endosymbiont BCDbulbsy
Desulfobulbus elongatus DSM_2908
Desulfobulbus propionicus DSM_2032
Desulfobulbus oralis HOT041_ORNL
Desulfobulbus sp Dsb1
Desulfobulbus sp oral taxon 041 str Dsb
Desulfobulbus sp oral taxon 041 str Dsb_1
Desulfobulbus sp oral taxon 041 str Dsb_2
Desulfobulbus japonicus DSM_18378
Desulfobulbus propionicus DOLJORAL78_50
Desulfobulbus mediterraneus DSM_13871
Desulfobulbus sp HyVt_436
Ca Desulfobulbus rimicarensis DR15
Ca Electronema sp GS
Desulfobulbaceae bacterium UBA5173
Ca Electrothrix aarhusiensis MCF
Ca Electrothrix communis A1
Ca Electrothrix marina A5
Desulfobacterales bacterium C00003060
Desulfobacterales bacterium S3730MH5
Desulfobacterales bacterium S5133MH4
Desulfobacterales bacterium S7086C20
Desulfobacterales bacterium C00003104
Desulfobacterales bacterium C00003106
Ca Magnetoglobus multicellularis str_Ar
Ca Magnetomorum sp HK_1
Desulfatibacillum aliphaticivorans AK_0
Desulfatibacillum aliphaticivorans DSM
Desulfatibacillum alkenivorans DSM_1621
Desulfococcus oleovorans Hxd3
Desulfococcus sp 4484_241
Desulfatirhabdium butyrativorans DSM_18
Desulfatirhabdium butyrativorans SpSt_4
Desulfobacteraceae bacterium UBA4064
Desulfobacteraceae bacterium UBA5605
Desulfobacteraceae bacterium UBA5746
Desulfobacteraceae bacterium RAAP_1
uncultured Desulfobacteraceae bacterium
Desulfobacteraceae bacterium IS3
Desulfobacteraceae bacterium UBA2174
Desulfobacteraceae bacterium UBA4769
Desulfobacteraceae bacterium 4572_187
Desulfobacteraceae bacterium Eth_SRB2
Desulfobacterales bacterium S5133MH16
Desulfobacterales bacterium NIOZ_UU17
Desulfobacterales bacterium PC51MH44
Desulfobacteraceae bacterium Eth SRB1
Desulfosarcina sp BuS5
Desulfobacteraceae bacterium A6
Desulfobacteraceae bacterium UBA2130
Desulfobacteraceae bacterium UBA2156
Desulfobacteraceae bacterium UBA5681
Desulfobacterales bacterium CG07_land_8
Desulfobacterales bacterium CG23_combo_1
Desulfobacteraceae bacterium UBA5616
Desulfatitalea sp BRH_c12
Desulfatitalea sp Site_C24
Desulfatitalea sp UBA4791
Desulfatitalea tepidiphila S28bF
Desulfobacteraceae bacterium UBA2230
Desulfobacteraceae bacterium UBA2771
Desulfosarcina sp Grevelingen_8MPall_me
Desulfosarcina alkanivorans PL12
Desulfosarcina widdelii PP31
Desulfosarcina cetonica JCM_12296
Desulfosarcina ovata subsp ovata oXyS1
Desulfosarcina ovata subsp sediminis 28
Desulfobacteraceae bacterium UBA2245
Desulfobacteraceae bacterium UBA4072
Desulfobacteraceae bacterium 4572_123
Desulfococcus multivorans DSM_2059
Desulfococcus multivorans DSM_2059_1
Desulfonema ishimotonii Tokyo_01
Desulfococcus sp DOLZORAL124_58_48
Desulfobacteraceae bacterium Bin4
Desulfobacteraceae bacterium UBA5852
Desulfobacteraceae bacterium SEEP_SAG9
Desulfobacterium sp SW_3_97
Desulfobacterales bacterium CG23_combo
Desulfosalsimonas propionicica DSM_1772
Desulfobotulus alkaliphilus ASO4_4
Desulfobotulus mexicanus PAR22N
Desulforegula conservatrix Mb1Pa
Desulfoluna butyratoxydans
Desulfoluna spongiiphila
Desulfoluna spongiiphila AA1
Desulfobacteraceae endosymbiont BCDbact
Desulfamplus magnetovallimortis PRJEB14
Desulfobacterium vacuolatum DSM_3385
Desulfobacterium autotrophicum HRM2
Desulfotignum balticum DSM_7044
Desulfotignum phosphitoxidans DSM 13687
Desulfotignum sp S09_Bin188
Desulfospira joergensenii DSM_10085
Desulfobacterales bacterium RIFOXYA12_F
Desulfobacula sp GWF2_41_7
Desulfobacula sp RIFOXYA12_FULL_46_16
Desulfobacula sp RIFOXYB2_FULL_45_6
Desulfobacula_phenolica_DSM_3384
Desulfobacula toluolica Tol2
Desulfobacula sp TS
Desulfobacteraceae bacterium Glo_11
Desulfobacula sp S2_67
Sym_BUSS2
Sym_SCH1
Desulfobacteraceae bacterium Glo_9
Desulfobacter curvatus DSM_3379
Desulfobacter hydrogenophilus AcRS1
Desulfobacter hydrogenophilus DSM_3380
Desulfobacter latus AcRS2
Desulfobacter postgatei DOLJORAL78_47_2
Desulfobacter postgatei 2ac9
Desulfobacter sp UBA12168
Desulfobacter sp UBA2225
Desulfobacter vibrioformis DSM_8776
Sym_BMAN
Sym_BMAN2
Desulfovibrio desulfuricans ND132
0.3

Figure S10: The *Anaeramoeba* symbionts belong to the *Desulfobacteraceae* family. Phylogenomic analysis is inferred from 168 taxa, 15 ribosomal proteins, and 2,226 sites. The ML tree was estimated with IQTree under the LG+C20+F+Gamma model of evolution. Bipartition support values are derived from 1,000 ultrafast bootstrap/SH-aLRT bootstraps. Scale bar indicates inferred number of substitutions per site. Tree files and alignments are available at FigShare: https://doi.org/10.6084/m9.figshare.20375601

Figure S11A: The distribution of IS elements in positional bins of 25 kb along the chromosome of Sym_BUSS2.

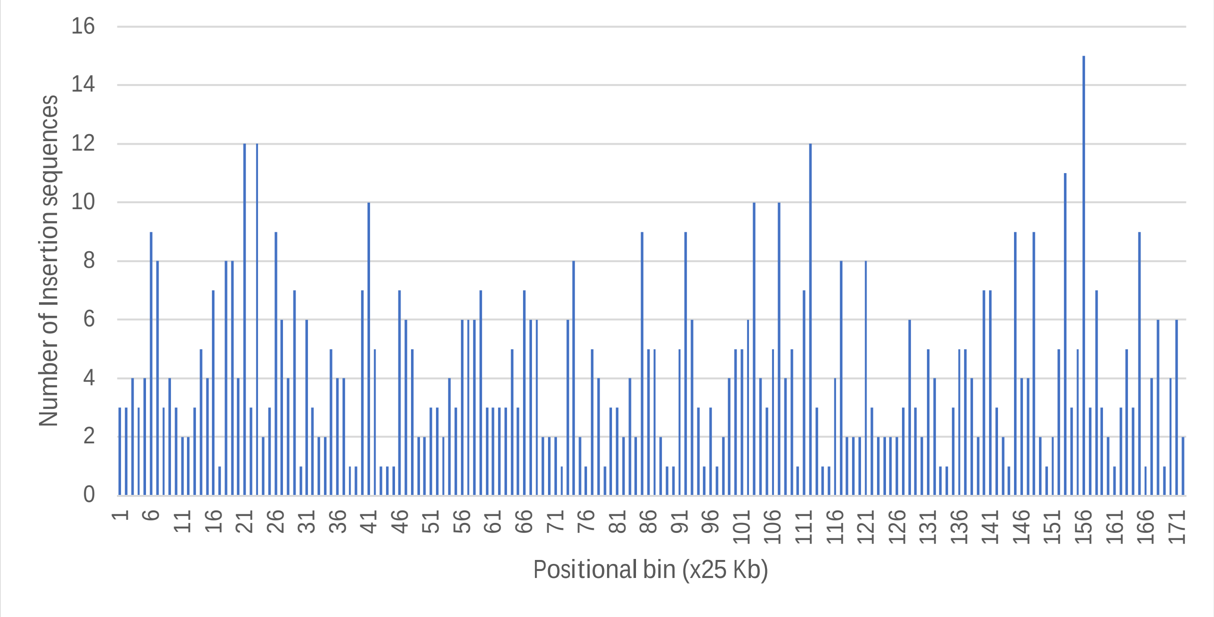

Figure S11B: The distribution of IS elements in positional bins of 25 kb along the chromosome of Sym_SCH1.

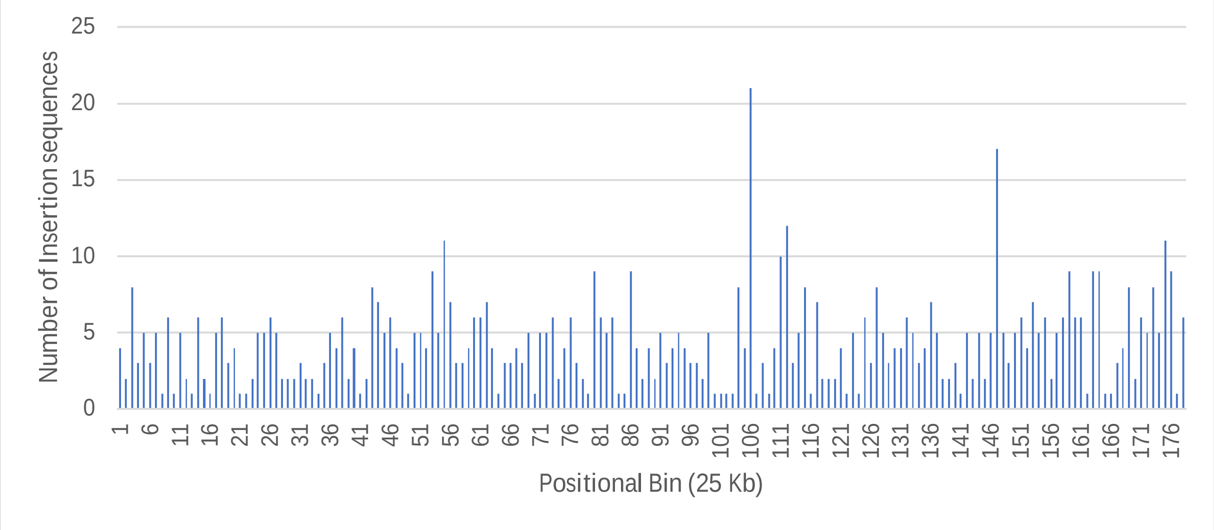

Figure S11C: Distance bins (500 bp) of IS elements positions of Sym_BUSS2 to a shift in synteny between Sym_BUSS2 and Sym_SCH1. The synteny blocks were inferred using Sibelia [1]. The five most common IS elements categories are shown.

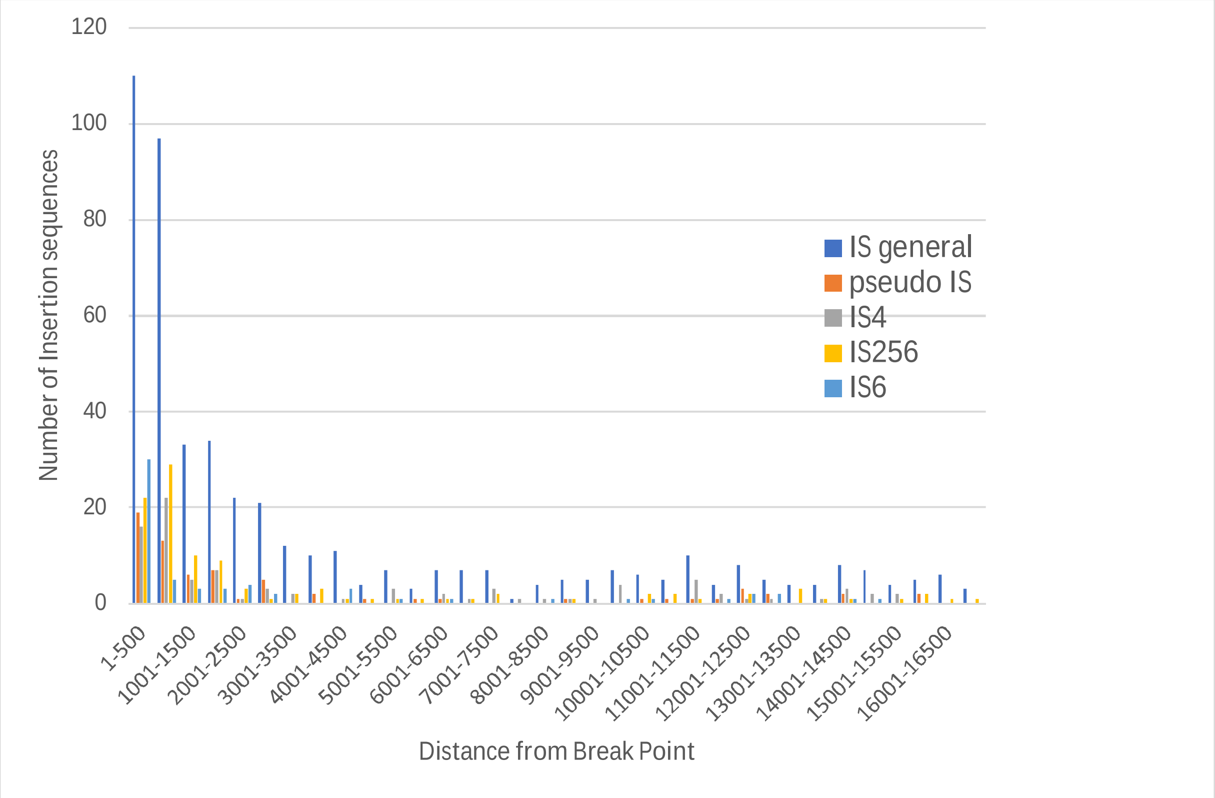

Figure S11D: Distance bins (500 bp) of IS elements positions of Sym_SCH1 to a shift in synteny between Sym_BUSS2 and Sym_SCH1. The synteny blocks were inferred using Sibelia [1]. The five most common IS elements categories are shown.

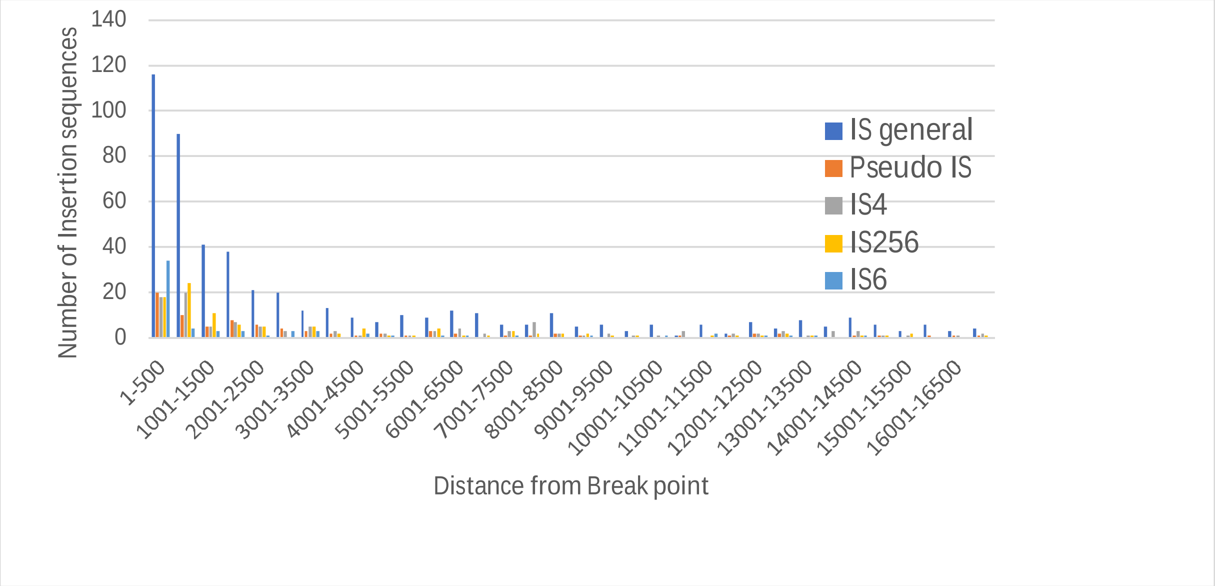

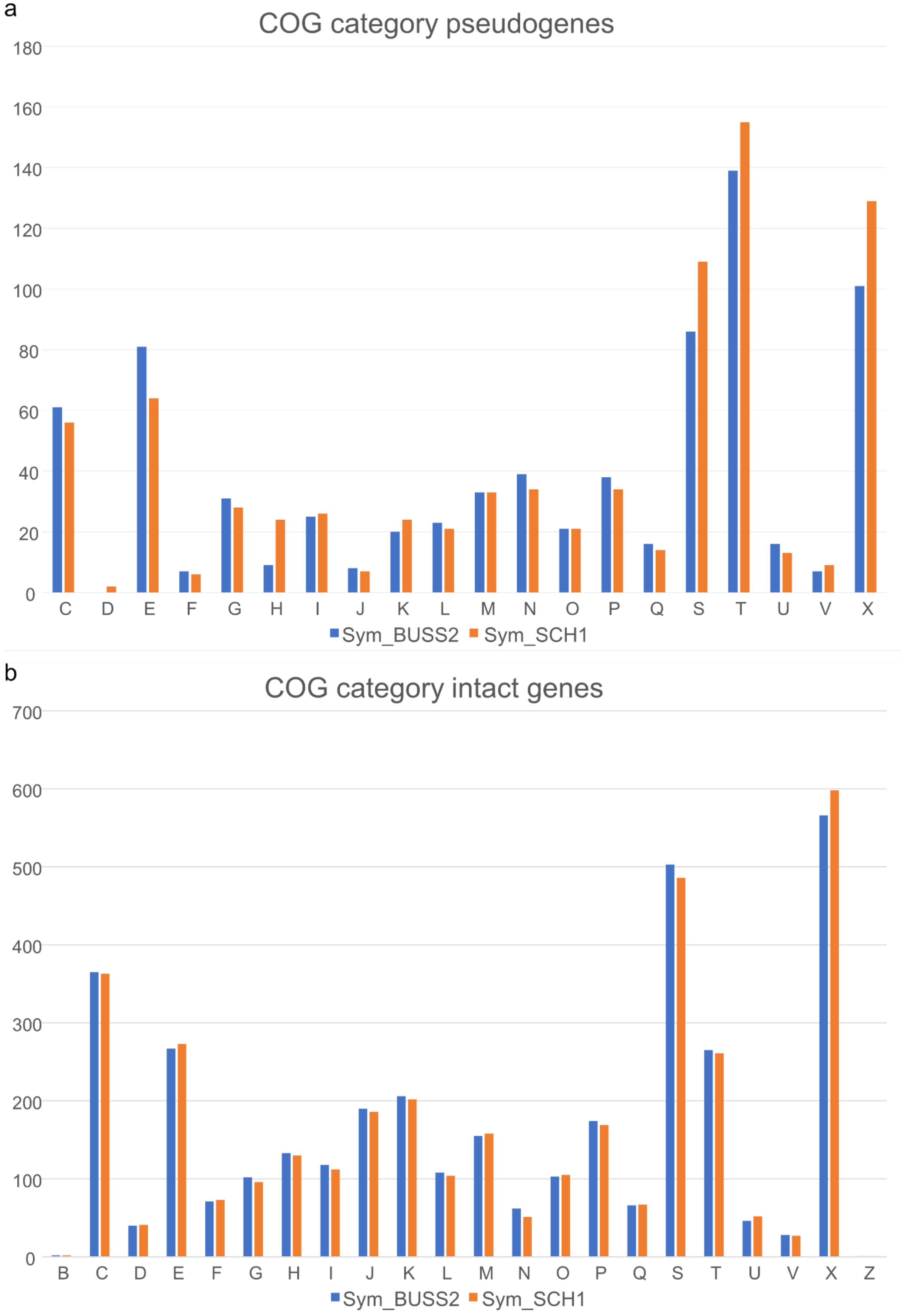
a
COG category pseudogenes
180
160
140
120
100
80
60
40
20
0
C D E F G H I J K L M N O P Q S T U V X
Sym_BUSS2 Sym_SCH1
b
COG category intact genes
700
600
500
400
300
200
100
0
B C D E F G H I J K L M N O P Q S T U V X Z
Sym_BUSS2 Sym_SCH1

Figure S12: Genes in signal transduction are commonly pseudogenized *A. flamelloides* symbionts. COG categories of *A. flamelloides* symbiont pseudogenes **a**, and intact genes **b,.** The COG categories of pseudogenes and intact genes for Sym_BUSS2 (blue) and Sym_SCH1 (orange) were determined by eggnog-mapper v2.1.7. Proteins classified in multiple COG categories were counted once in each category. The y-axis shows the number of classifications. Abbreviations: B - Chromatin structure and dynamics, C - Energy production and conversion, D - Cell cycle control and mitosis, E - Amino acid metabolism and transport, F - Nucleotide metabolism and transport, G - Carbohydrate metabolism and transport, H - Coenzyme metabolism, I - Lipid metabolism, J - Translation, K - Transcription, L - Replication and repair, M - Cell wall/membrane/envelope biogenesis, N - Cell motility, O - Post-translational modification, protein turnover, chaperone functions, P - Inorganic ion transport and metabolism, Q - Secondary Structure, T - Signal transduction, U - Intracellular trafficking and secretion, Y - Nuclear structure, Z - Cytoskeleton, R - General functional prediction only, S - Function unknown, V - Defense mechanisms, X - Mobilome: prophages, transposons.

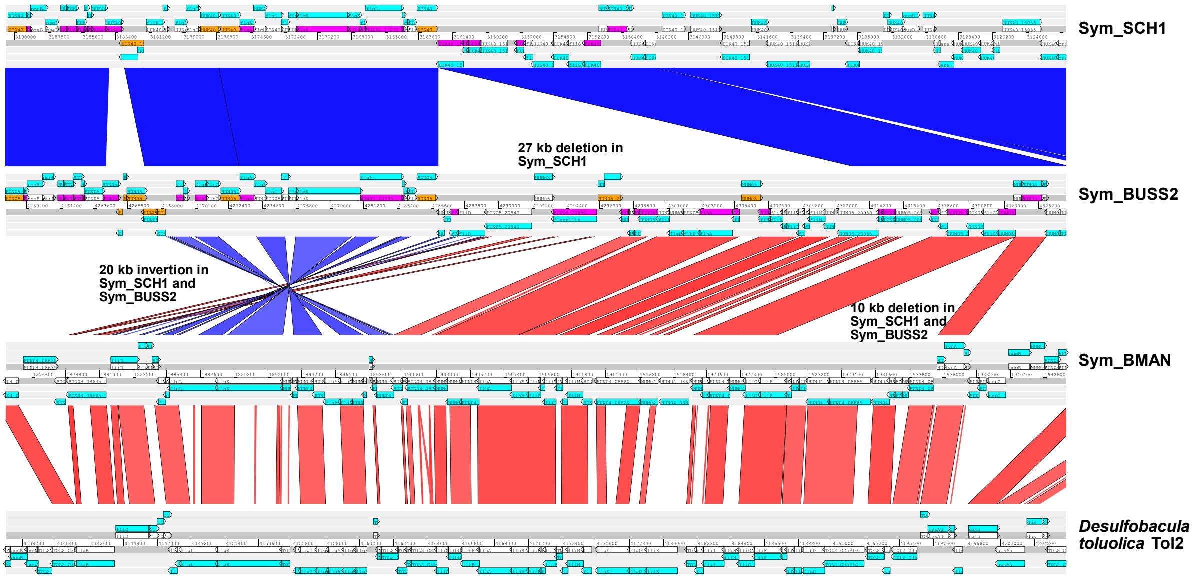

Figure S13: Pseudogenization of the flagellar operon in Sym_SCH1 and Sym_BUSS2. A synteny plot of the flagellar operons of Sym_SCH1, Sym_BUSS2, Sym_BMAN and the free-living *Desulfobacula toluolica* Tol2. Sym_BMAN and *Desulfobacula toluolica* Tol2 share conserved gene order and lack pseudogenes (purple) and IS elements (orange). Sym_SCH1 and Sym_BUSS2 have experienced substantial genome decay in this region. This is due to deletions, inversions, frame-shift pseudogenization as well as IS element induced pseudogenization. The part of the genome upstream on the flagellar operon in Sym_SCH1 shows no evidence of decay. Syntenic regions as determined by blastn similarity. Select IS element connections were pruned to IS to increase viewability.

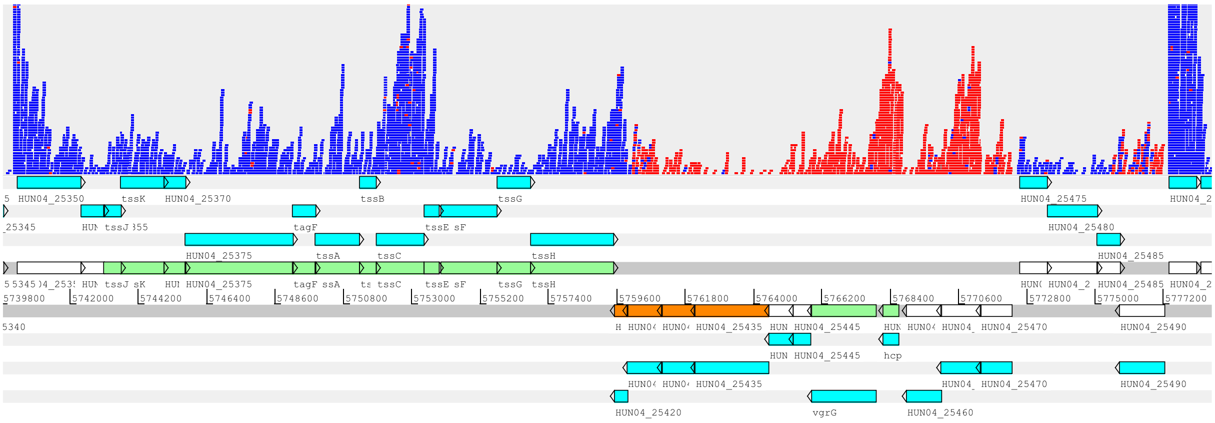

Figure S14: Type VI secretion system in Sym_BMAN. Sym_BMAN encodes a type VI secretion system (T6SS) that consists of two convergent transcription units of 25 genes, with most genes showing evidence of expression. The first transcription unit includes 12 genes (*tssJKLM*, *tagF*, *tssA*, *tssBC*, *tssEFGH*) and two hypothetical proteins. The second transcription unit encodes, *vgrG*, *hcp*, and upstream of these; a forkhead associated domain (FHA) protein, a TPR repeat protein, a carbohydrate esterase 4 (CE4) superfamily protein. The downstream genes include a DUF4280 family protein previously suggested as potential spike (PAAR) proteins [2]. Directly downstream of *vgrG*, we identified a DUF4123 protein (HUN04_25440), which is generally encoded upstream of putative T6SS effectors (Liang et al. 2015). The four effector candidates (HUN04_25435, HUN04_25430, HUN04_25425, HUN04_25420) encoded between the DUF4123 domain protein and DUF4280 domain protein have no known function, but putative homologs are present in both free-living and bacterial symbiont genomes. Light green – conserved T6SS genes. Orange – effector candidate genes.

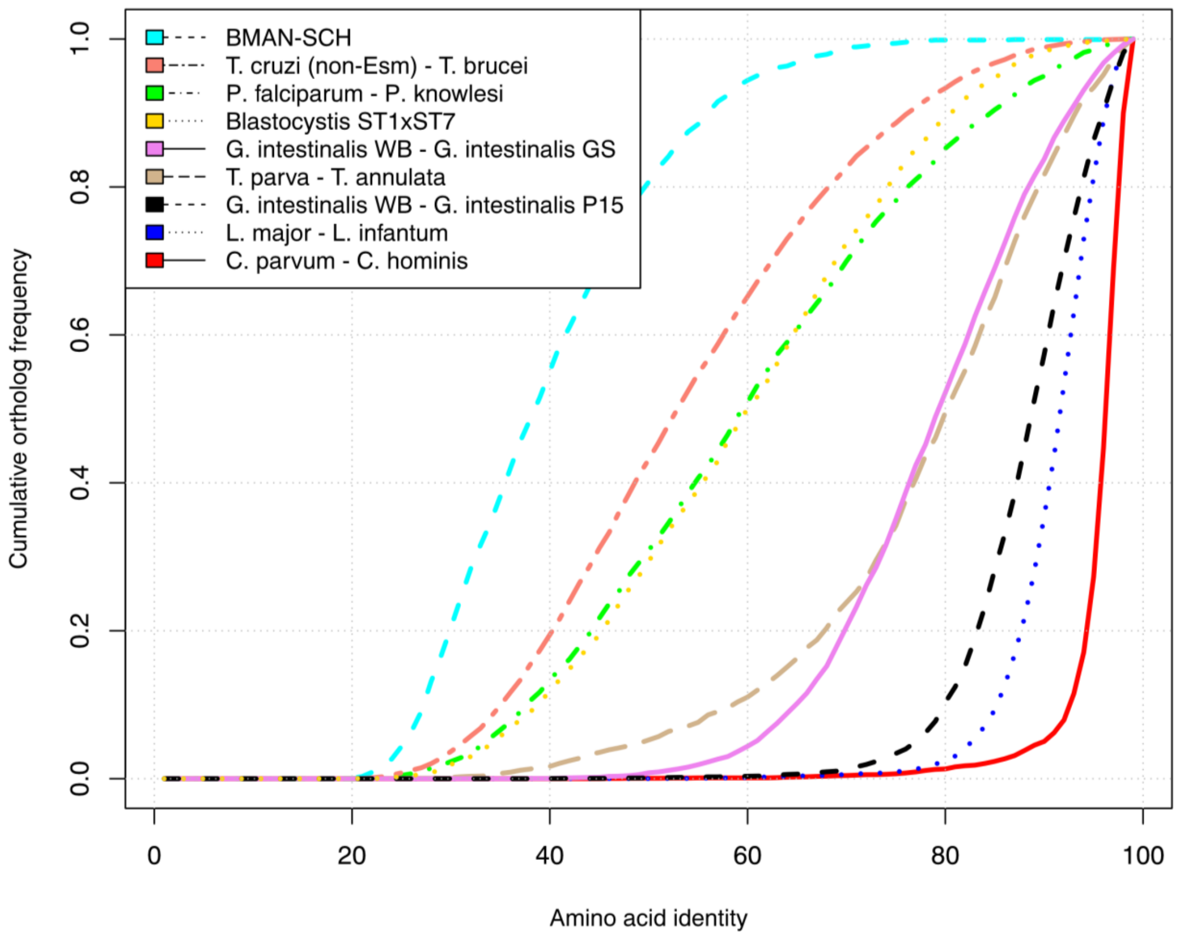

Figure S15: Comparison of amino acid identities between *Anaeramoeba* species to other eukaryotes. Cumulative ortholog frequency is plotted on the y-axis while the x-axis represents the amino acid identity between orthologs. The more similar species pairs are to each other, the steeper the curve and further to the right. Note that the order of the pairs in the legend matches the order in the figure. Comparisons for *Anaeramoeba* species were based on aligned regions of reciprocal best BLAST hits, while non-*Anaeramoeba* data were obtained from the authors of a comparative study of *Giardia intestinalis* [3] and *Blastocystis* ST1 [4].

a

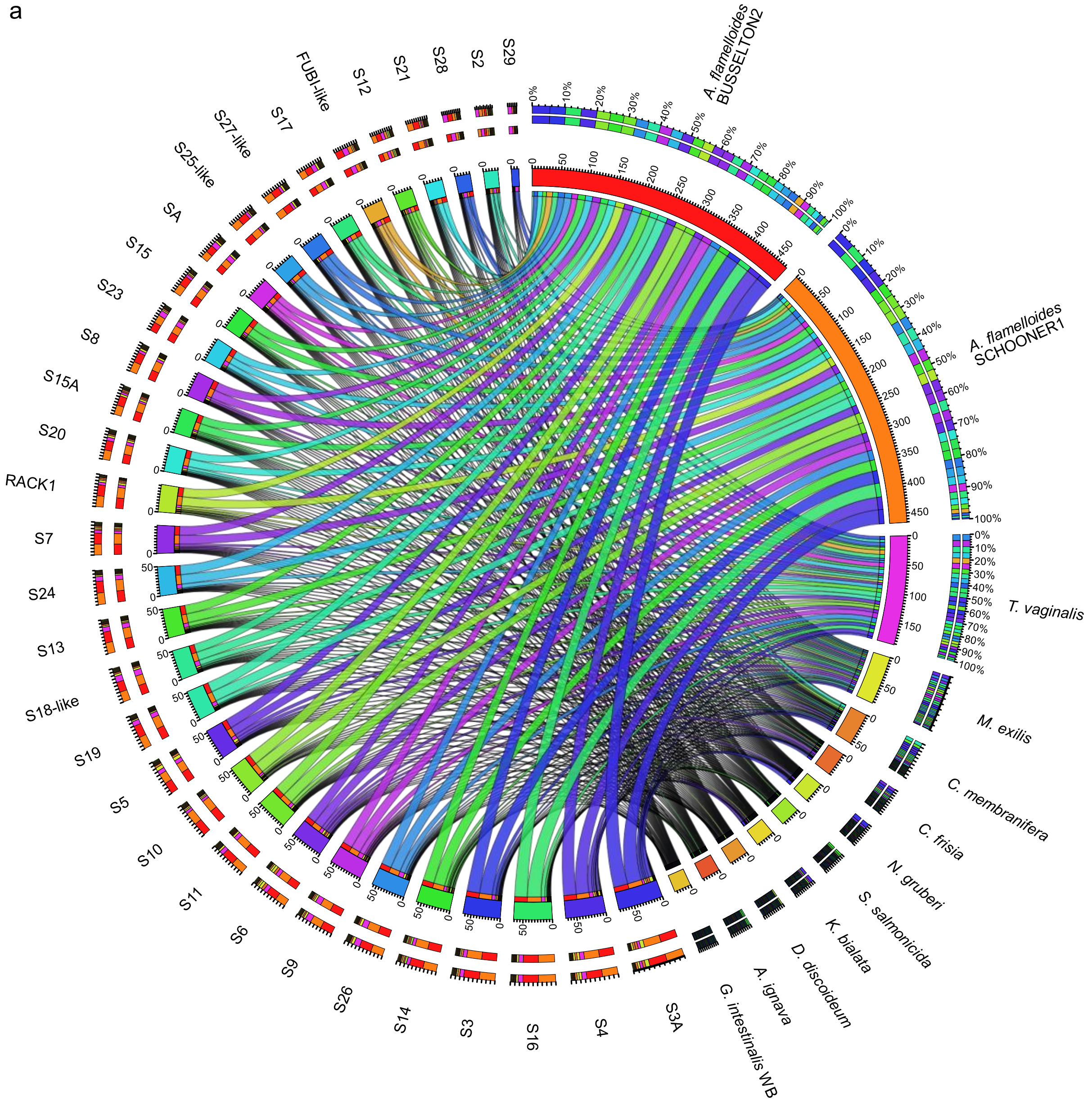

A. flamelloides BUSSELTON2
A. flamelloides SCHOONER1
T. vaginalis
M. exilis
C. membranifera
C. frisia
N. gruberi
S. salmonicida
K. bialata
D. discoideum
A. ignava
G. intestinalis WB
S3A
S4
S16
S3
S14
S26
S9
S6
S11
S10
S5
S19
S18-like
S13
S24
S7
RACK1
S20
S15A
S8
S23
S15
SA
S25-like
S27-like
S17
FUBI-like
S12
S21
S28
S2
S29

b

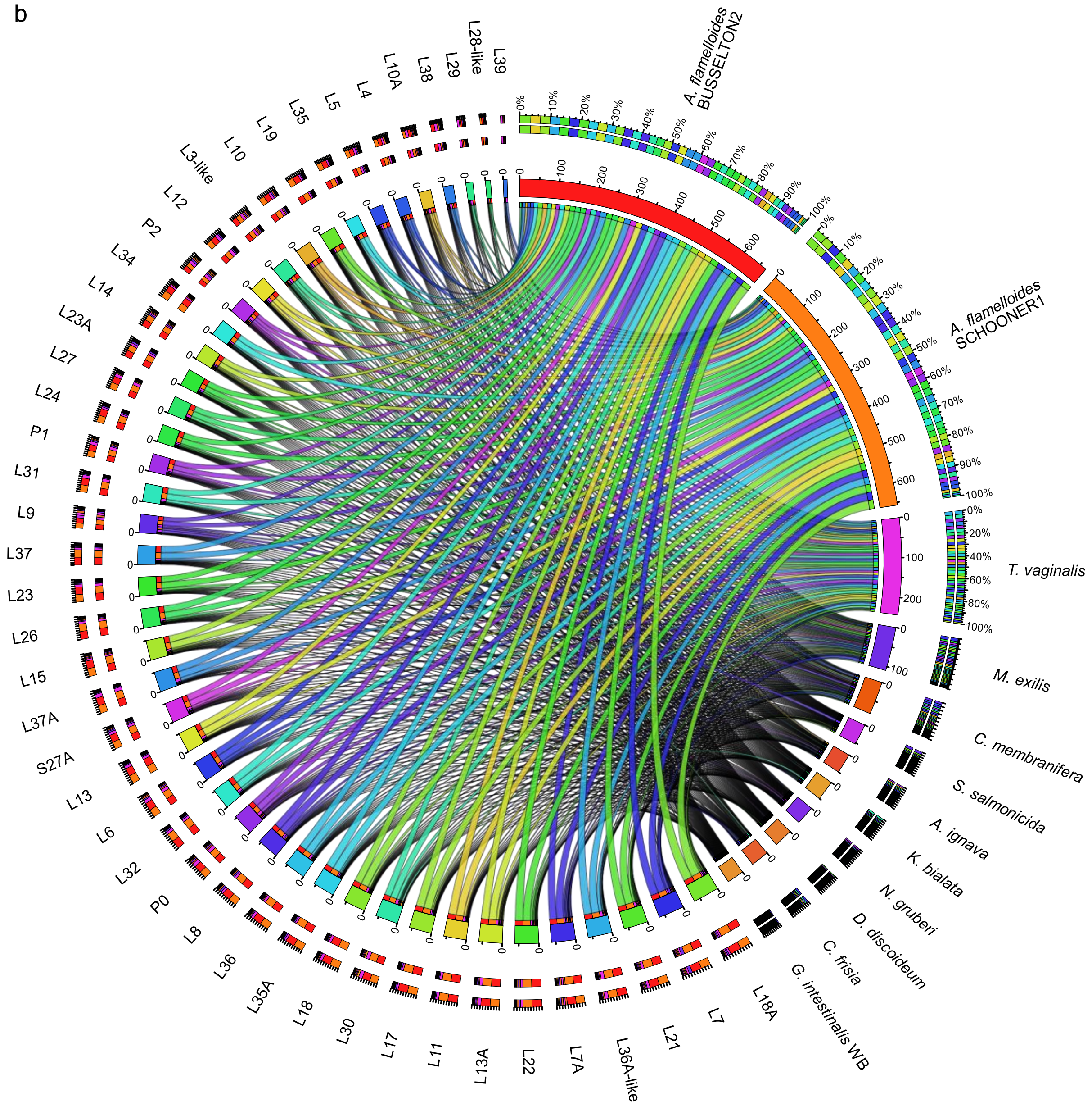

Figure S16: Expanded copy number of ribosomal proteins (RP) in *A. flamelloides*. The *A. flamelloides* genome is highly amplified in **a,** 40S ribosomal subunits, and **b,** 60S ribosomal subunits. Color-coded taxa [BUSSELTON2 – *G. intestinalis* WB] are depicted clockwise and sorted from the largest to the smallest number of RP orthologs detected in each taxon [S3A-S29 or L18A-L39]. Each color-coded ribbon from each taxon represents the RP ortholog specified in the opposite end of the ribbon and the width of the ribbon indicates the size of the expansion. The percentage of the expansion within each taxon matches the color-coded ribbon and it is depicted in the outer circle next to each taxon label. The outer circle next to the RP orthologs is color-coded by taxon. For example, in figure **a,** BUSSELTON2 largest expansions are for the orthologs S3A and S4 with 26 and 23 proteins, respectively (an approximation of these counts is shown in the inner red circle under the taxon name that is marked from 0 to 450 with small divisions each indicating 5 proteins). The expansions of these two orthologs amount to 10% of all the considered RP orthologs in BUSSELTON2. In terms of S3A and S4, the outer circle indicates that most proteins for such orthologues belong to BUSSELTON2 (red) and SCHOONER1 (orange).

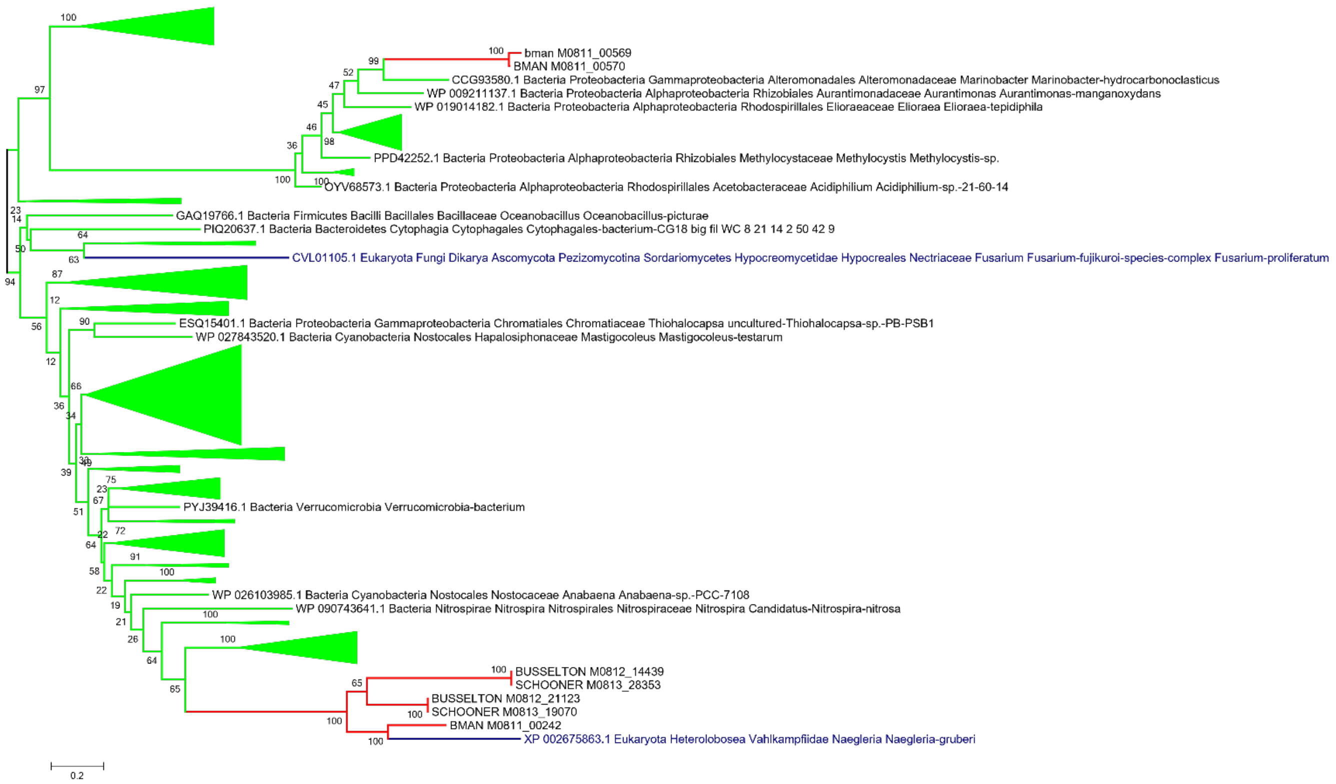

Figure S17A Phylogenetic reconstruction of 3-hydroxybutyrate dehydrogenase. Alignment with MAFFT, site selection BMGE and phylogeny IQTree model LG4X, 1000 ultrafast bootstraps. *Anaeramoeba* sequences are in red, eukaryotic sequences in blue, and prokaryotic sequences in bright green

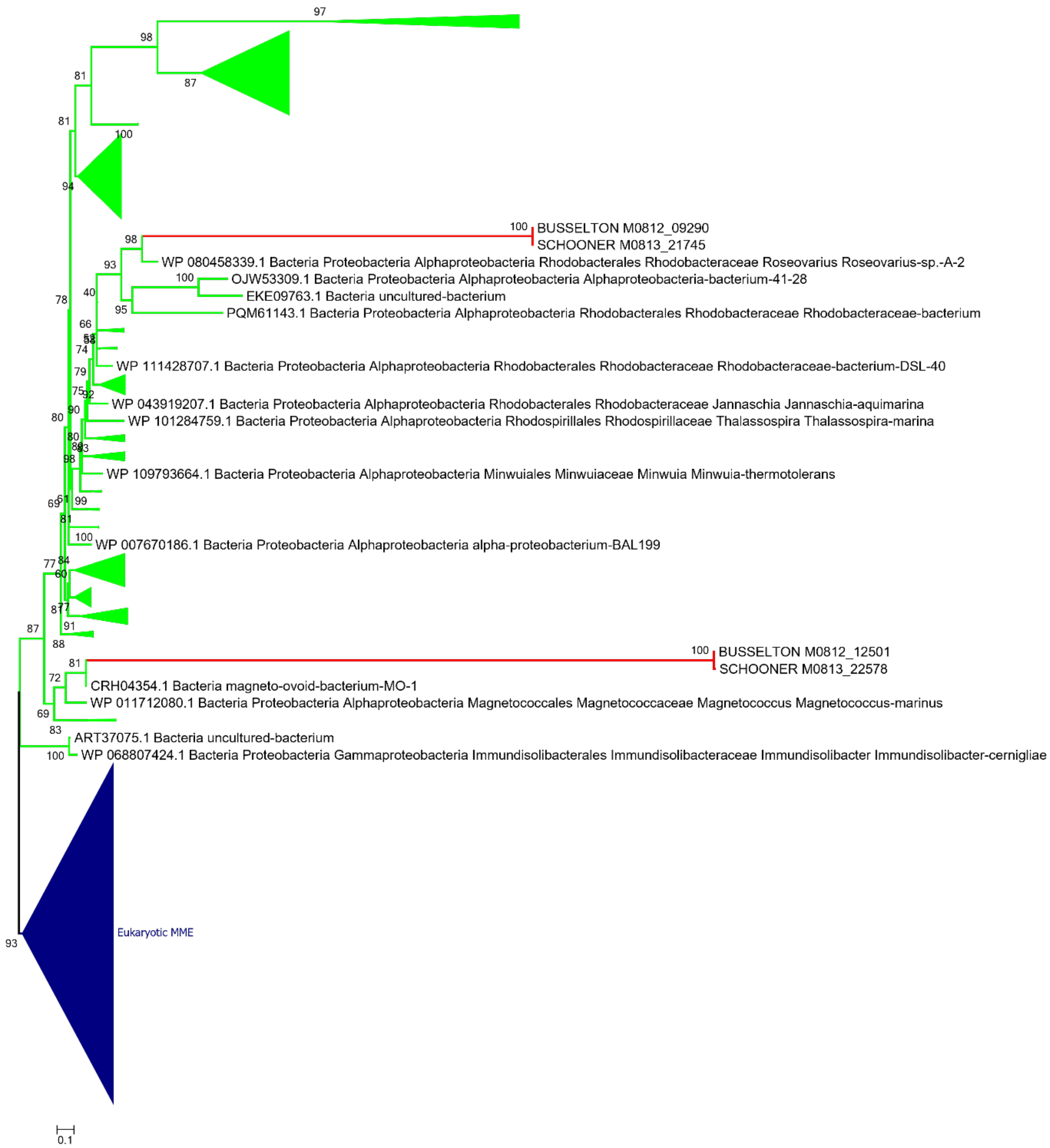

Figure S17B Phylogenetic reconstruction of Methylmalonyl CoA epimerase (MME). Alignment with MAFFT, site selection BMGE and phylogeny IQTree model C20 1000 ultrafast bootstrap. *Anaeramoeba* sequences are in red, eukaryotic sequences in blue, and prokaryotic sequences in bright green.

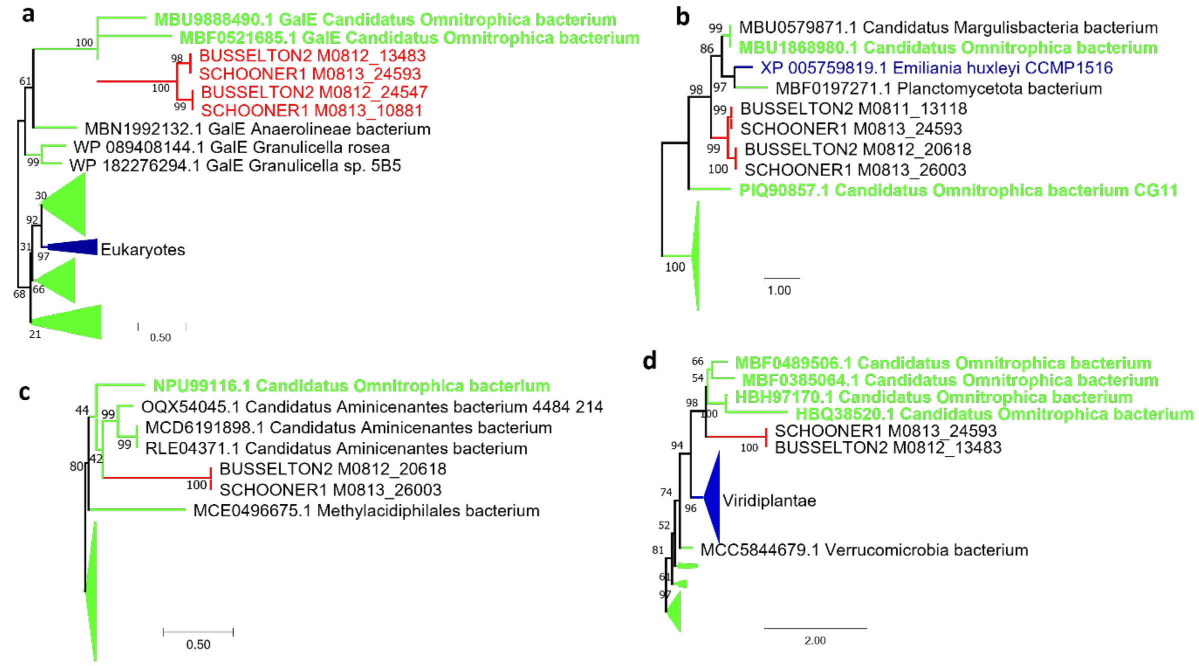

Figure S17C Phylogenetic reconstruction of the 4 enzymes constituting the Leloir pathway. Alignment with MAFFT, site selection BMGE, and phylogeny IQTree model C60 with 1000 ultrafast bootstrap. *Anaeramoeba* sequences are in red, eukaryotic sequences in blue, and prokaryotic sequences in bright green. Candidatus omnitrophica bacterium labels are in green bold characters. **a**, UDP-galactose 4 epimerase (GalE). **b**, galactokinase (GalK). **c**, galactose mutarotase (GalM). **d**, galactose 1-P uridyltransferase (GalT).

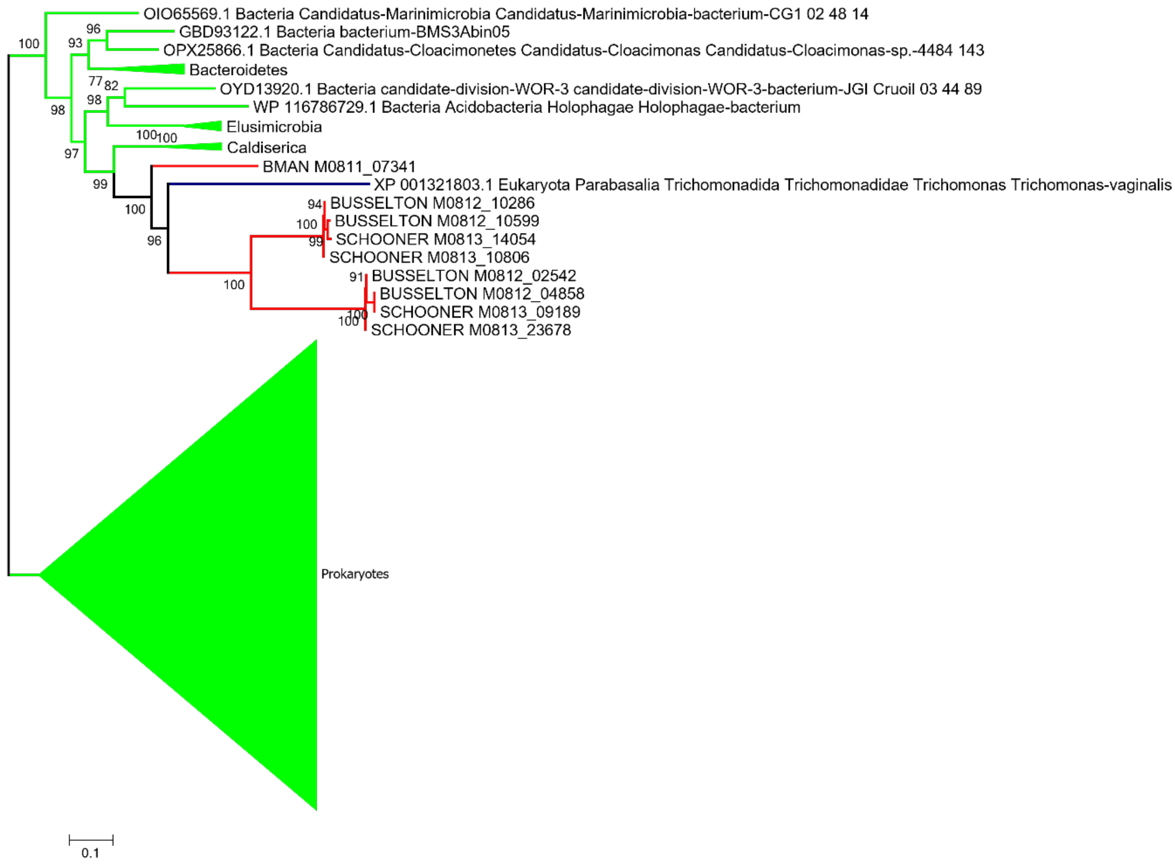

Figure S17D Phylogenetic reconstruction of CysK. Alignment with MAFFT, site selection BMGE and phylogeny IQTree model LG4X 1000 ultrafast bootstrap. *Anaeramoeba* sequences are in red, eukaryotic sequences in blue, and prokaryotic sequences in bright green.

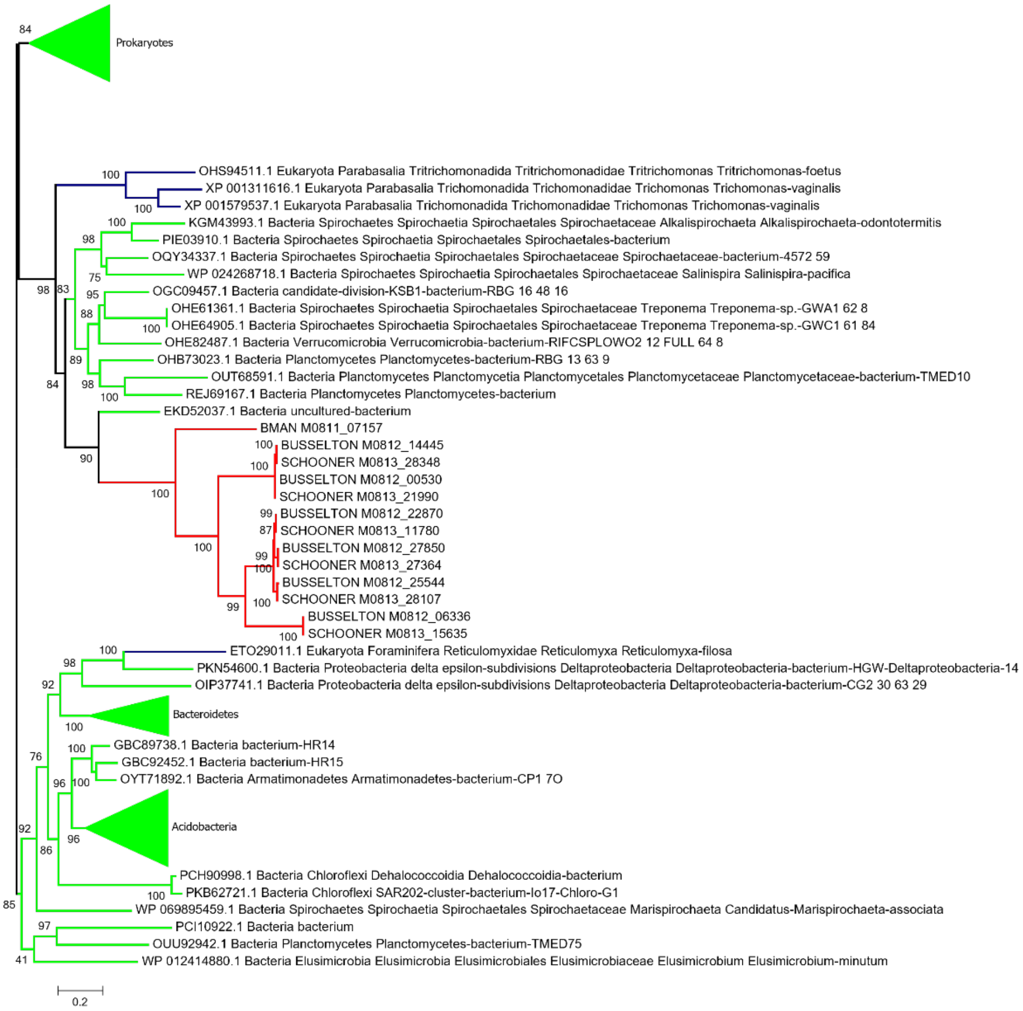

Figure S17E Phylogenetic reconstruction of D-phosphoglycerate dehydrogenase. Alignment with MAFFT, site selection BMGE and phylogeny IQTree model LG4X 1000 ultrafast bootstrap. *Anaeramoeba* sequences are in red, eukaryotic sequences in blue, and prokaryotic sequences in bright green.

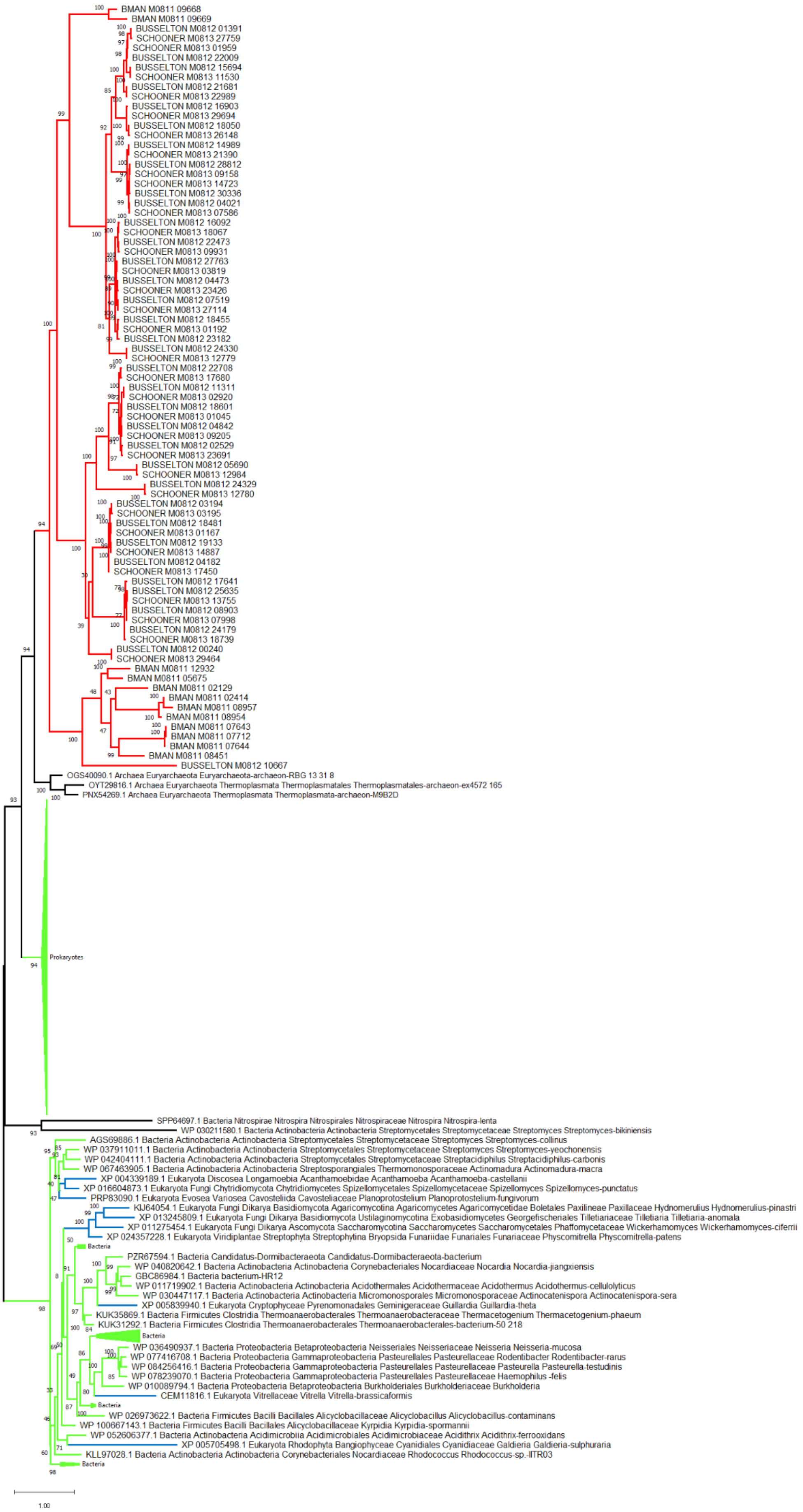

BMAN M0811 09668
BMAN M0811 09669
BUSSELTON M0812 01391
SCHOONER M0813 27759
SCHOONER M0813 01959
BUSSELTON M0812 22009
BUSSELTON M0812 15694
SCHOONER M0813 11530
BUSSELTON M0812 21681
SCHOONER M0813 22989
BUSSELTON M0812 16903
SCHOONER M0813 29694
BUSSELTON M0812 18050
SCHOONER M0813 26148
BUSSELTON M0812 14989
SCHOONER M0813 21390
BUSSELTON M0812 28812
SCHOONER M0813 09158
SCHOONER M0813 14723
BUSSELTON M0812 30336
BUSSELTON M0812 04021
SCHOONER M0813 07586
BUSSELTON M0812 16092
SCHOONER M0813 18067
BUSSELTON M0812 22473
SCHOONER M0813 09931
BUSSELTON M0812 27763
SCHOONER M0813 03819
BUSSELTON M0812 04473
SCHOONER M0813 23426
BUSSELTON M0812 07519
SCHOONER M0813 27114
BUSSELTON M0812 18455
SCHOONER M0813 01192
BUSSELTON M0812 23182
BUSSELTON M0812 24330
SCHOONER M0813 12779
BUSSELTON M0812 22708
SCHOONER M0813 17680
BUSSELTON M0812 11311
SCHOONER M0813 02920
BUSSELTON M0812 18601
SCHOONER M0813 01045
BUSSELTON M0812 04842
SCHOONER M0813 09205
BUSSELTON M0812 02529
SCHOONER M0813 23691
BUSSELTON M0812 05690
SCHOONER M0813 12984
BUSSELTON M0812 24329
SCHOONER M0813 12780
BUSSELTON M0812 03194
SCHOONER M0813 03195
BUSSELTON M0812 18481
SCHOONER M0813 01167
BUSSELTON M0812 19133
SCHOONER M0813 14887
BUSSELTON M0812 04182
SCHOONER M0813 17450
BUSSELTON M0812 17641
BUSSELTON M0812 25635
SCHOONER M0813 13755
BUSSELTON M0812 08903
SCHOONER M0813 07998
BUSSELTON M0812 24179
SCHOONER M0813 18739
BUSSELTON M0812 00240
SCHOONER M0813 29464
BMAN M0811 12932
BMAN M0811 05675
BMAN M0811 02129
BMAN M0811 02414
BMAN M0811 08957
BMAN M0811 08954
BMAN M0811 07643
BMAN M0811 07712
BMAN M0811 07644
BMAN M0811 08451
BUSSELTON M0812 10667
OGS40090.1 Archaea Euryarchaeota Euryarchaeota-archaeon-RBG 13 31 8
OYT29816.1 Archaea Euryarchaeota Thermoplasmata Thermoplasmatales Thermoplasmatales-archaeon-ex4572 165
PNX54269.1 Archaea Euryarchaeota Thermoplasmata Thermoplasmata-archaeon-M9B2D
Prokaryotes
SPP64697.1 Bacteria Nitrospirae Nitrospira Nitrospirales Nitrospiraceae Nitrospira Nitrospira-lenta
WP 030211580.1 Bacteria Actinobacteria Actinobacteria Streptomycetales Streptomycetaceae Streptomyces Streptomyces-bikiniensis
AGS69886.1 Bacteria Actinobacteria Actinobacteria Streptomycetales Streptomycetaceae Streptomyces Streptomyces-collinus
WP 037911011.1 Bacteria Actinobacteria Actinobacteria Streptomycetales Streptomycetaceae Streptomyces Streptomyces-yeochonensis
WP 042404111.1 Bacteria Actinobacteria Actinobacteria Streptomycetales Streptomycetaceae Streptacidiphilus Streptacidiphilus-carbonis
WP 067463905.1 Bacteria Actinobacteria Actinobacteria Streptosporangiales Thermomonosporaceae Actinomadura Actinomadura-macra
XP 004339189.1 Eukaryota Discosea Longamoebia Acanthamoebidae Acanthamoeba Acanthamoeba-castellanii
XP 016604873.1 Eukaryota Fungi Chytridiomycota Chytridiomycetes Spizellomycetales Spizellomycetaceae Spizellomyces Spizellomyces-punctatus
PRP83090.1 Eukaryota Evosea Variosea Cavosteliida Cavosteliaceae Planoprotostelium Planoprotostelium-fungivorum
KIJ64054.1 Eukaryota Fungi Dikarya Basidiomycota Agaricomycotina Agaricomycetes Agaricomycetidae Boletales Paxilineae Paxillaceae Hydnomerulius Hydnomerulius-pinastri
XP 013245809.1 Eukaryota Fungi Dikarya Basidiomycota Ustilaginomycotina Exobasidiomycetes Georgefischeriales Tilletiariaceae Tilletiaria Tilletiaria-anomala
XP 011275454.1 Eukaryota Fungi Dikarya Ascomycota Saccharomycotina Saccharomycetes Saccharomycetales Phaffomycetaceae Wickerhamomyces Wickerhamomyces-ciferrii
XP 024357228.1 Eukaryota Viridiplantae Streptophyta Streptophytina Bryopsida Funariidae Funariales Funariaceae Physcomitrella Physcomitrella-patens
Bacteria
PZR67594.1 Bacteria Candidatus-Dormibacteraeota Candidatus-Dormibacteraeota-bacterium
WP 040820642.1 Bacteria Actinobacteria Actinobacteria Corynebacteriales Nocardiaceae Nocardia Nocardia-jiangxiensis
GBC86984.1 Bacteria bacterium-HR12
WP 011719902.1 Bacteria Actinobacteria Actinobacteria Acidothermales Acidothermaceae Acidothermus Acidothermus-cellulolyticus
WP 030447117.1 Bacteria Actinobacteria Actinobacteria Micromonosporales Micromonosporaceae Actinocatenispora Actinocatenispora-sera
XP 005839940.1 Eukaryota Cryptophyceae Pyrenomonadales Geminigeraceae Guillardia Guillardia-theta
KUK35869.1 Bacteria Firmicutes Clostridia Thermoanaerobacterales Thermoanaerobacteraceae Thermacetogenium Thermacetogenium-phaeum
KUK31292.1 Bacteria Firmicutes Clostridia Thermoanaerobacterales Thermoanaerobacterales-bacterium-50 218
Bacteria
WP 036490937.1 Bacteria Proteobacteria Betaproteobacteria Neisseriales Neisseriaceae Neisseria Neisseria-mucosa
WP 077416708.1 Bacteria Proteobacteria Gammaproteobacteria Pasteurellales Pasteurellaceae Rodentibacter Rodentibacter-rarus
WP 084256416.1 Bacteria Proteobacteria Gammaproteobacteria Pasteurellales Pasteurellaceae Pasteurella Pasteurella-testudinis
WP 078239070.1 Bacteria Proteobacteria Gammaproteobacteria Pasteurellales Pasteurellaceae Haemophilus -felis
WP 010089794.1 Bacteria Proteobacteria Betaproteobacteria Burkholderiales Burkholderiaceae Burkholderia
CEM11816.1 Eukaryota Vitrellaceae Vitrella Vitrella-brassicaformis
Bacteria
WP 026973622.1 Bacteria Firmicutes Bacilli Bacillales Alicyclobacillaceae Alicyclobacillus Alicyclobacillus-contaminans
WP 100667143.1 Bacteria Firmicutes Bacilli Bacillales Alicyclobacillaceae Kyrpidia Kyrpidia-spormannii
WP 052606377.1 Bacteria Actinobacteria Acidimicrobiia Acidimicrobiales Acidimicrobiaceae Acidithrix Acidithrix-ferrooxidans
XP 005705498.1 Eukaryota Rhodophyta Bangiophyceae Cyanidiales Cyanidiaceae Galdieria Galdieria-sulphuraria
KLL97028.1 Bacteria Actinobacteria Actinobacteria Corynebacteriales Nocardiaceae Rhodococcus Rhodococcus-sp.-IITR03
Bacteria
1.00

Figure S17F Phylogenetic reconstruction of Acetate transporters. Alignment with MAFFT, site selection BMGE and phylogeny IQTree model LG4X, 1000 ultrafast bootstrap. *Anaeramoeba* sequences are in red, eukaryotic sequences in blue, and prokaryotic sequences in bright green.

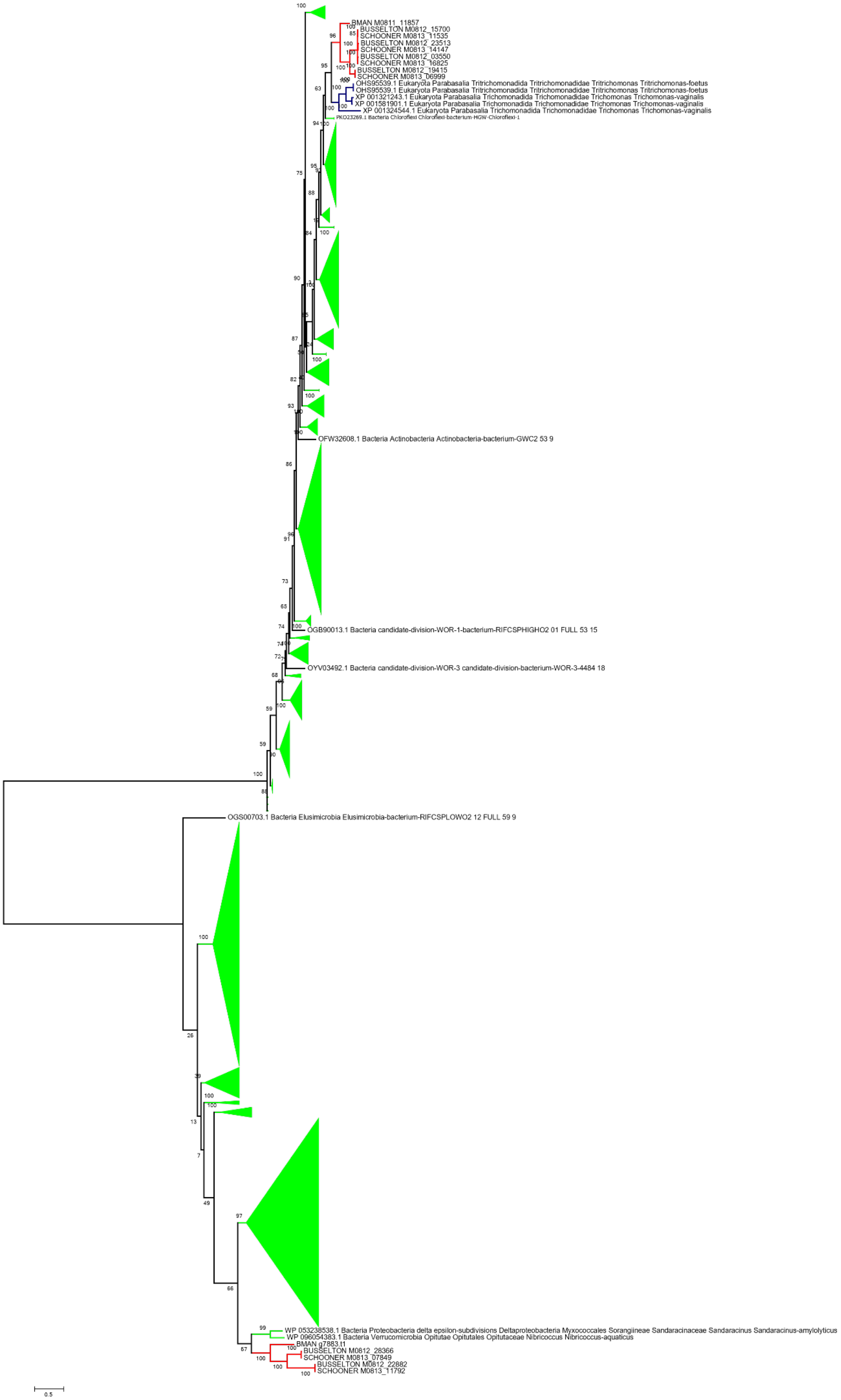

BMAN M0811_11857
BUSSELTON M0812_15700
SCHOONER M0813_11535
BUSSELTON M0812_23513
SCHOONER M0813_14147
BUSSELTON M0812_03550
SCHOONER M0813_16825
BUSSELTON M0812_19415
SCHOONER M0813_06999
OHS95539.1 Eukaryota Parabasalia Tritrichomonadida Tritrichomonadidae Tritrichomonas Tritrichomonas-foetus
OHS95539.1 Eukaryota Parabasalia Tritrichomonadida Tritrichomonadidae Tritrichomonas Tritrichomonas-foetus
XP 001321243.1 Eukaryota Parabasalia Trichomonadida Trichomonadidae Trichomonas Trichomonas-vaginalis
XP 001581901.1 Eukaryota Parabasalia Trichomonadida Trichomonadidae Trichomonas Trichomonas-vaginalis
XP 001324544.1 Eukaryota Parabasalia Trichomonadida Trichomonadidae Trichomonas Trichomonas-vaginalis
PKO23269.1 Bacteria Chloroflexi Chloroflexi-bacterium-HGW-Chloroflexi-1
OFW32608.1 Bacteria Actinobacteria Actinobacteria-bacterium-GWC2 53 9
OGB90013.1 Bacteria candidate-division-WOR-1-bacterium-RIFCSPHIGHO2 01 FULL 53 15
OYV03492.1 Bacteria candidate-division-WOR-3 candidate-division-bacterium-WOR-3-4484 18
OGS00703.1 Bacteria Elusimicrobia Elusimicrobia-bacterium-RIFCSPLOWO2 12 FULL 59 9
WP 053238538.1 Bacteria Proteobacteria delta epsilon-subdivisions Deltaproteobacteria Myxococcales Sorangiineae Sandaracinaceae Sandaracinus Sandaracinus-amylolyticus
WP 096054383.1 Bacteria Verrucomicrobia Opitutae Opitutales Opitutaceae Nibricoccus Nibricoccus-aquaticus
BMAN g7883.t1
BUSSELTON M0812_28366
SCHOONER M0813_07849
BUSSELTON M0812_22882
SCHOONER M0813_11792
0.5

Figure S17G Phylogenetic reconstruction of pyruvate phosphate dikinase. Alignment with MAFFT, site selection BMGE and phylogeny IQTree model LG4X 1000 ultrafast bootstrap. *Anaeramoeba* sequences are in red, eukaryotic sequences in blue, and prokaryotic sequences in bright green.

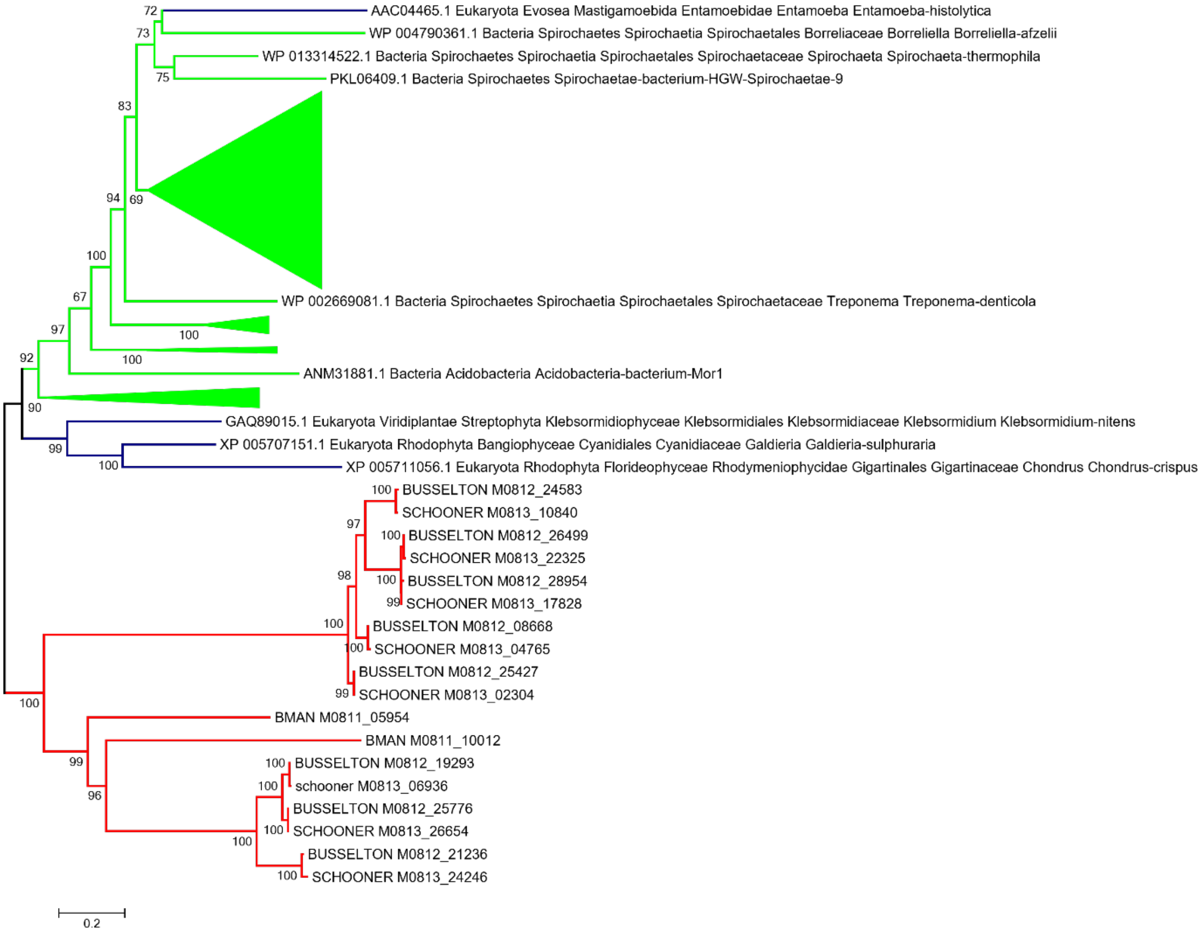

Figure S17H Phylogenetic reconstruction of ATP-independent phosphofructokinase. Alignment with MAFFT, site selection BMGE and phylogeny IQTree model LG4X 1000 ultrafast bootstrap. *Anaeramoeba* sequences are in red, eukaryotic sequences in blue, and prokaryotic sequences in bright green.

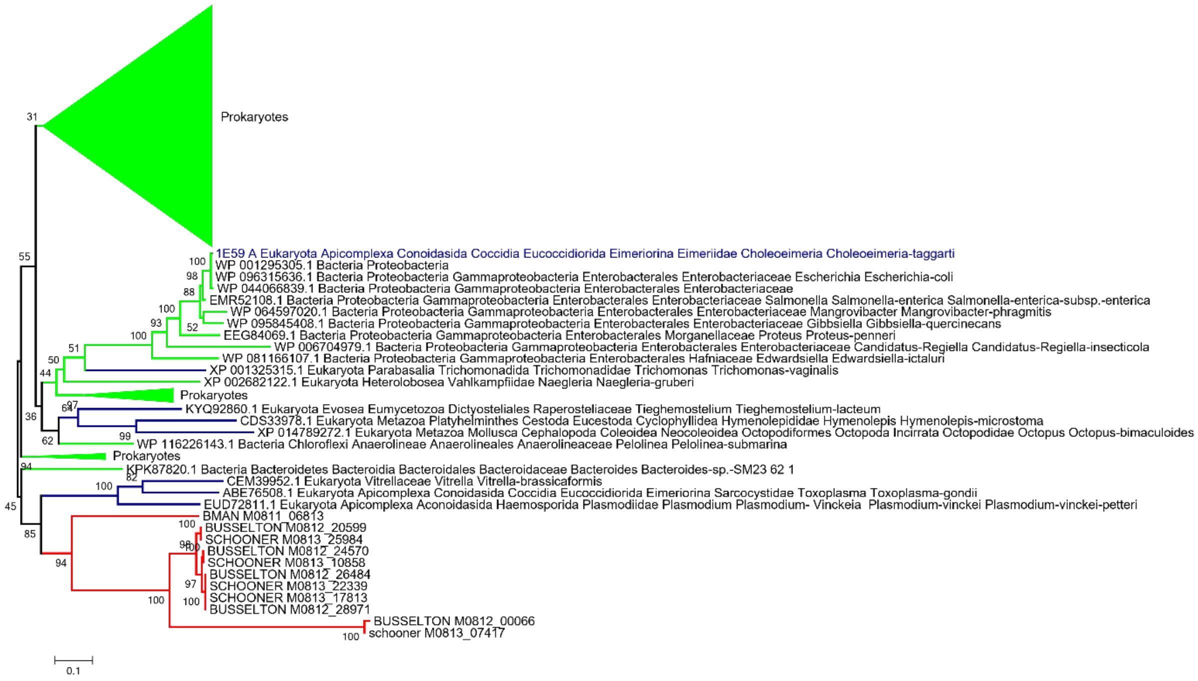

Figure S17I Phylogenetic reconstruction of Phosphoglycerate mutase (OG 363). Alignment with MAFFT, site selection BMGE and phylogeny IQTree model LG4X 1000 ultrafast bootstrap. *Anaeramoeba* sequences are in red, eukaryotic sequences in blue, and prokaryotic sequences in bright green.

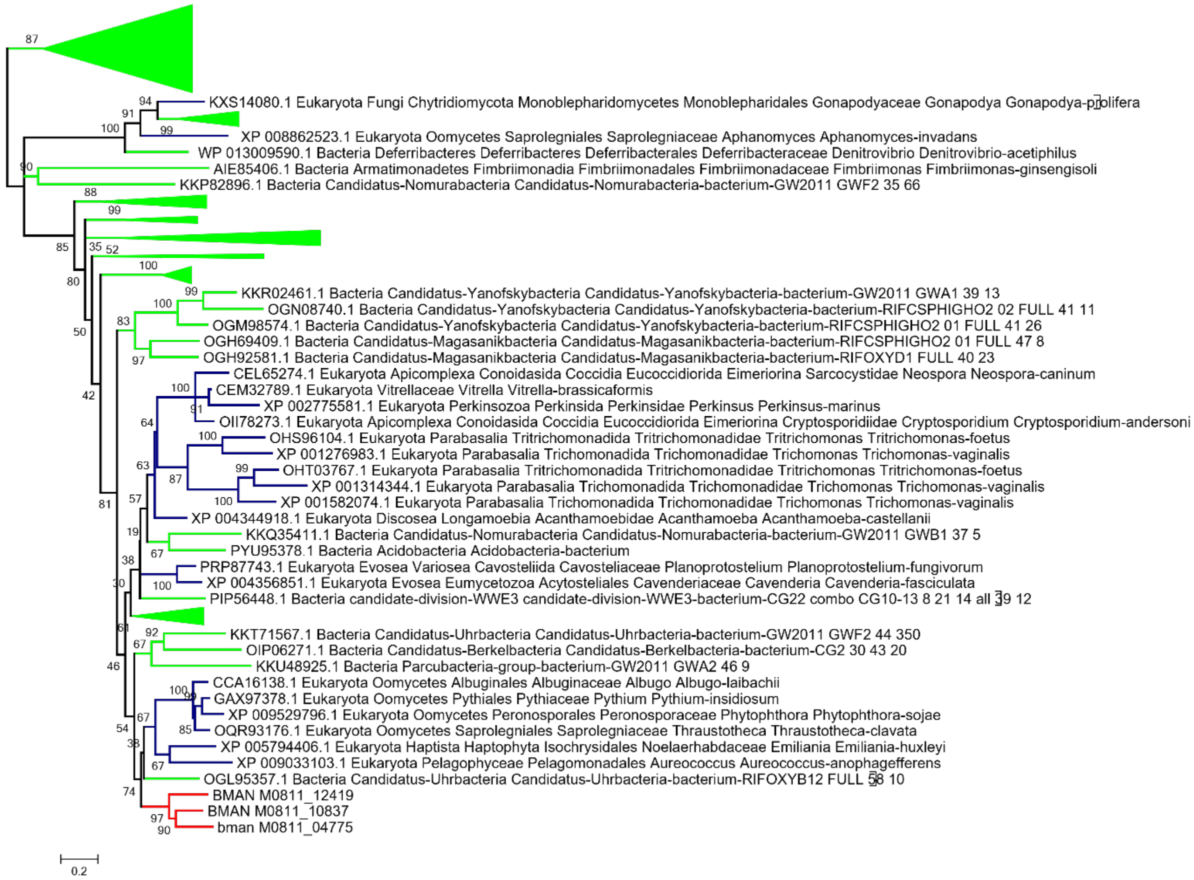

Figure S17J Phylogenetic reconstruction of Phosphoglycerate mutase (OG 2532). Alignment with MAFFT, site selection BMGE and phylogeny IQTree model LG4X,1,000 ultrafast bootstrap. *Anaeramoeba* sequences are in red, eukaryotic sequences in blue, and prokaryotic sequences in bright green.

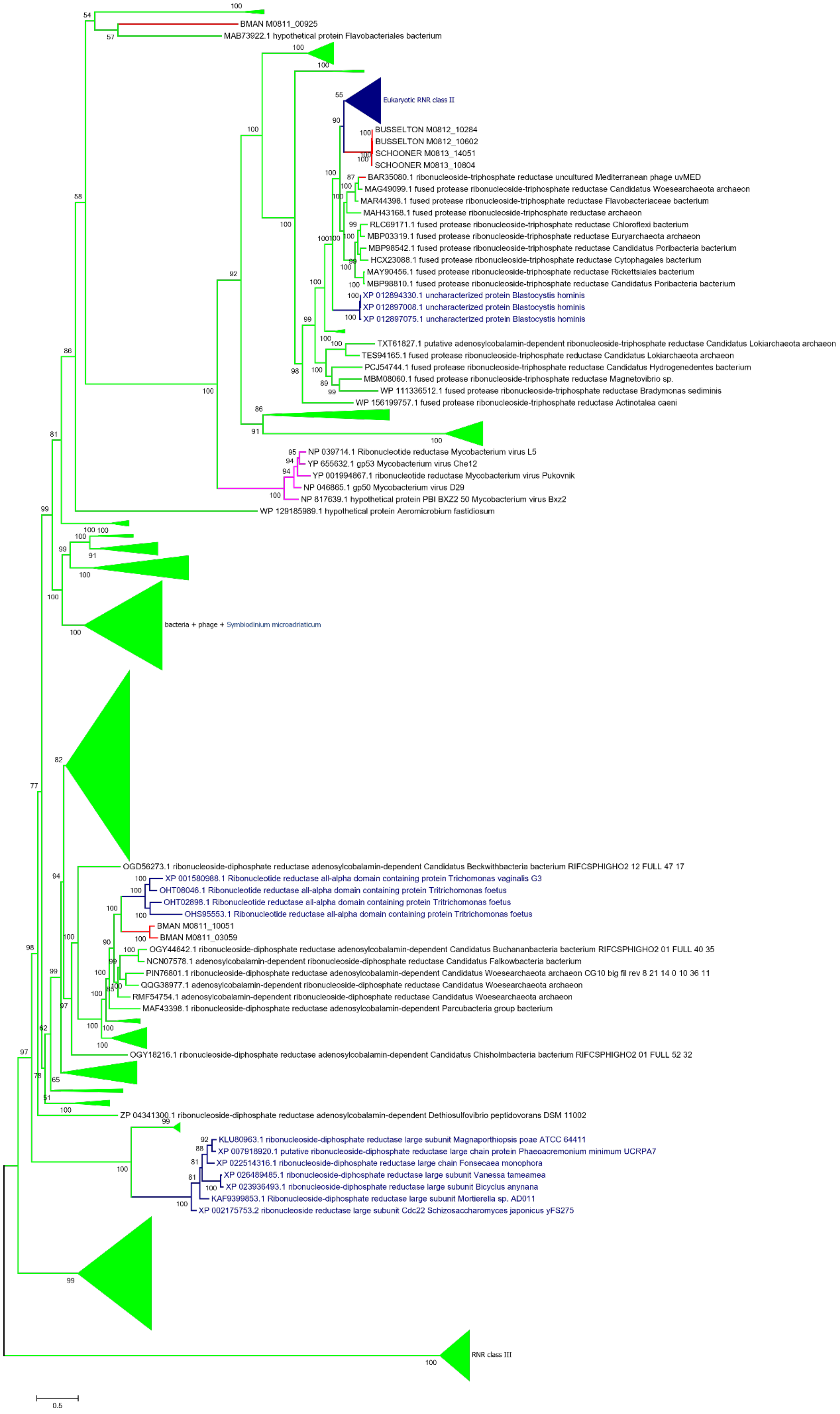

BMAN M0811_00925
MAB73922.1 hypothetical protein Flavobacteriales bacterium
Eukaryotic RNR class II
BUSSELTON M0812_10284
BUSSELTON M0812_10602
SCHOONER M0813_14051
SCHOONER M0813_10804
BAR35080.1 ribonucleoside-triphosphate reductase uncultured Mediterranean phage uvMED
MAG49099.1 fused protease ribonucleoside-triphosphate reductase Candidatus Woesearchaeota archaeon
MAR44398.1 fused protease ribonucleoside-triphosphate reductase Flavobacteriaceae bacterium
MAH43168.1 fused protease ribonucleoside-triphosphate reductase archaeon
RLC69171.1 fused protease ribonucleoside-triphosphate reductase Chloroflexi bacterium
MBP03319.1 fused protease ribonucleoside-triphosphate reductase Euryarchaeota archaeon
MBP98542.1 fused protease ribonucleoside-triphosphate reductase Candidatus Poribacteria bacterium
HCX23088.1 fused protease ribonucleoside-triphosphate reductase Cytophagales bacterium
MAY90456.1 fused protease ribonucleoside-triphosphate reductase Rickettsiales bacterium
MBP98810.1 fused protease ribonucleoside-triphosphate reductase Candidatus Poribacteria bacterium
XP 012894330.1 uncharacterized protein Blastocystis hominis
XP 012897008.1 uncharacterized protein Blastocystis hominis
XP 012897075.1 uncharacterized protein Blastocystis hominis
TXT61827.1 putative adenosylcobalamin-dependent ribonucleoside-triphosphate reductase Candidatus Lokiarchaeota archaeon
TES94165.1 fused protease ribonucleoside-triphosphate reductase Candidatus Lokiarchaeota archaeon
PCJ54744.1 fused protease ribonucleoside-triphosphate reductase Candidatus Hydrogenedentes bacterium
MBM08060.1 fused protease ribonucleoside-triphosphate reductase Magnetovibrio sp.
WP 111336512.1 fused protease ribonucleoside-triphosphate reductase Bradymonas sediminis
WP 156199757.1 fused protease ribonucleoside-triphosphate reductase Actinotalea caeni
NP 039714.1 Ribonucleotide reductase Mycobacterium virus L5
YP 655632.1 gp53 Mycobacterium virus Che12
YP 001994867.1 ribonucleotide reductase Mycobacterium virus Pukovnik
NP 046865.1 gp50 Mycobacterium virus D29
NP 817639.1 hypothetical protein PBI BXZ2 50 Mycobacterium virus Bxz2
WP 129185989.1 hypothetical protein Aeromicrobium fastidiosum
bacteria + phage + Symbiodinium microadriaticum
OGD56273.1 ribonucleoside-diphosphate reductase adenosylcobalamin-dependent Candidatus Beckwithbacteria bacterium RIFCSPHIGHO2 12 FULL 47 17
XP 001580988.1 Ribonucleotide reductase all-alpha domain containing protein Trichomonas vaginalis G3
OHT08046.1 Ribonucleotide reductase all-alpha domain containing protein Tritrichomonas foetus
OHT02898.1 Ribonucleotide reductase all-alpha domain containing protein Tritrichomonas foetus
OHS95553.1 Ribonucleotide reductase all-alpha domain containing protein Tritrichomonas foetus
BMAN M0811_10051
BMAN M0811_03059
OGY44642.1 ribonucleoside-diphosphate reductase adenosylcobalamin-dependent Candidatus Buchananbacteria bacterium RIFCSPHIGHO2 01 FULL 40 35
NCN07578.1 adenosylcobalamin-dependent ribonucleoside-diphosphate reductase Candidatus Falkowbacteria bacterium
PIN76801.1 ribonucleoside-diphosphate reductase adenosylcobalamin-dependent Candidatus Woesearchaeota archaeon CG10 big fil rev 8 21 14 0 10 36 11
QQG38977.1 adenosylcobalamin-dependent ribonucleoside-diphosphate reductase Candidatus Woesearchaeota archaeon
RMF54754.1 adenosylcobalamin-dependent ribonucleoside-diphosphate reductase Candidatus Woesearchaeota archaeon
MAF43398.1 ribonucleoside-diphosphate reductase adenosylcobalamin-dependent Parcubacteria group bacterium
OGY18216.1 ribonucleoside-diphosphate reductase adenosylcobalamin-dependent Candidatus Chisholmbacteria bacterium RIFCSPHIGHO2 01 FULL 52 32
ZP 04341300.1 ribonucleoside-diphosphate reductase adenosylcobalamin-dependent Dethiosulfovibrio peptidovorans DSM 11002
KLU80963.1 ribonucleoside-diphosphate reductase large subunit Magnaporthiopsis poae ATCC 64411
XP 007918920.1 putative ribonucleoside-diphosphate reductase large chain protein Phaeoacremonium minimum UCRPA7
XP 022514316.1 ribonucleoside-diphosphate reductase large chain Fonsecaea monophora
XP 026489485.1 ribonucleoside-diphosphate reductase large subunit Vanessa tameamea
XP 023936493.1 ribonucleoside-diphosphate reductase large subunit Bicyclus anynana
KAF9399853.1 Ribonucleoside-diphosphate reductase large subunit Mortierella sp. AD011
XP 002175753.2 ribonucleoside reductase large subunit Cdc22 Schizosaccharomyces japonicus yFS275
RNR class III
0.5

Figure S17K Phylogenetic reconstruction of Ribonucleotide reductases. Alignment with MAFFT, selection of sites with less than 30% gaps and phylogeny IQTree model C20+G4 1000 ultrafast bootstrap. *Anaeramoeba* sequences are in red, eukaryotic sequences in blue, viral sequences in light purple and prokaryotic sequences in bright green.

.

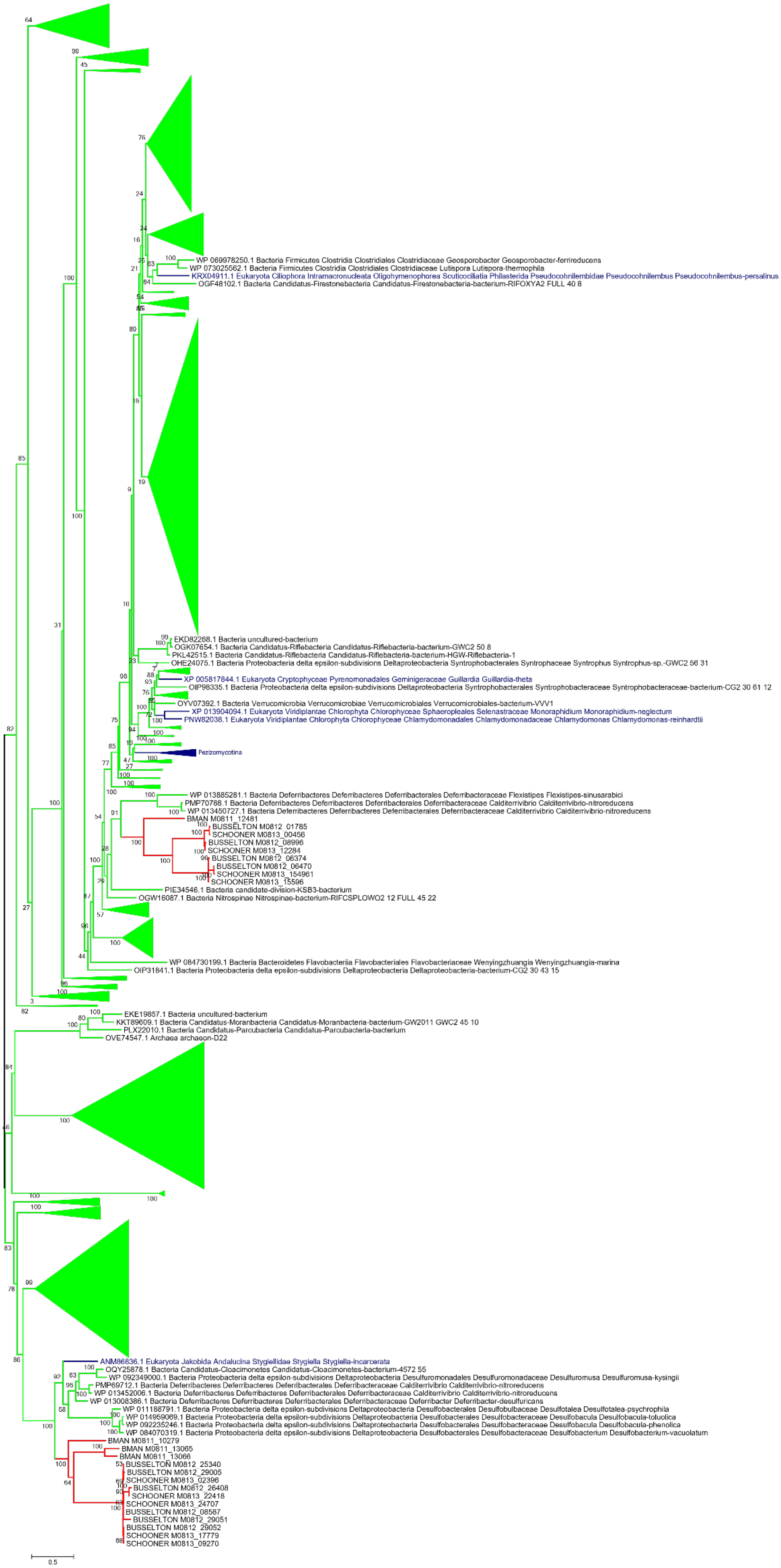
WP 069978250.1 Bacteria Firmicutes Clostridia Clostridiales Clostridiaceae Geosporobacter Geosporobacter-ferrireducens
WP 073025562.1 Bacteria Firmicutes Clostridia Clostridiales Clostridiaceae Lutispora Lutispora-thermophila
KRX04911.1 Eukaryota Ciliophora Intramacronucleata Oligohymenophorea Scuticociliatia Philasterida Pseudocohnilembidae Pseudocohnilembus Pseudocohnilembus-persalinus
OGF48102.1 Bacteria Candidatus-Firestonebacteria Candidatus-Firestonebacteria-bacterium-RIFOXYA2 FULL 40 8
EKD82268.1 Bacteria uncultured-bacterium
OGK07654.1 Bacteria Candidatus-Riflebacteria Candidatus-Riflebacteria-bacterium-GWC2 50 8
PKL42515.1 Bacteria Candidatus-Riflebacteria Candidatus-Riflebacteria-bacterium-HGW-Riflebacteria-1
OHE24075.1 Bacteria Proteobacteria delta epsilon-subdivisions Deltaproteobacteria Syntrophobacterales Syntrophaceae Syntrophus Syntrophus-sp.-GWC2 56 31
XP 005817844.1 Eukaryota Cryptophyceae Pyrenomonadales Geminigeraceae Guillardia Guillardia-theta
OIP98335.1 Bacteria Proteobacteria delta epsilon-subdivisions Deltaproteobacteria Syntrophobacterales Syntrophobacteraceae Syntrophobacteraceae-bacterium-CG2 30 61 12
OYV07392.1 Bacteria Verrucomicrobia Verrucomicrobiae Verrucomicrobiales Verrucomicrobiales-bacterium-VVV1
XP 013904094.1 Eukaryota Viridiplantae Chlorophyta Chlorophyceae Sphaeropleales Selenastraceae Monoraphidium Monoraphidium-neglectum
PNW82038.1 Eukaryota Viridiplantae Chlorophyta Chlorophyceae Chlamydomonadales Chlamydomonadaceae Chlamydomonas Chlamydomonas-reinhardtii
Pezizomycotina
WP 013885281.1 Bacteria Deferribacteres Deferribacteres Deferribacterales Deferribacteraceae Flexistipes Flexistipes-sinusarabici
PMP70788.1 Bacteria Deferribacteres Deferribacteres Deferribacterales Deferribacteraceae Calditerrivibrio Calditerrivibrio-nitroreducens
WP 013450727.1 Bacteria Deferribacteres Deferribacteres Deferribacterales Deferribacteraceae Calditerrivibrio Calditerrivibrio-nitroreducens
BMAN M0811_12481
BUSSELTON M0812_01785
SCHOONER M0813_00456
BUSSELTON M0812_08996
SCHOONER M0813_12284
BUSSELTON M0812_06374
BUSSELTON M0812_06470
SCHOONER M0813_154961
SCHOONER M0813_15596
PIE34546.1 Bacteria candidate-division-KSB3-bacterium
OGW16087.1 Bacteria Nitrospinae Nitrospinae-bacterium-RIFCSPLOWO2 12 FULL 45 22
WP 084730199.1 Bacteria Bacteroidetes Flavobacteriia Flavobacteriales Flavobacteriaceae Wenyingzhuangia Wenyingzhuangia-marina
OIP31841.1 Bacteria Proteobacteria delta epsilon-subdivisions Deltaproteobacteria Deltaproteobacteria-bacterium-CG2 30 43 15
EKE19857.1 Bacteria uncultured-bacterium
KKT89609.1 Bacteria Candidatus-Moranbacteria Candidatus-Moranbacteria-bacterium-GW2011 GWC2 45 10
PLX22010.1 Bacteria Candidatus-Parcubacteria Candidatus-Parcubacteria-bacterium
OVE74547.1 Archaea archaeon-D22
ANM86836.1 Eukaryota Jakobida Andalucina Stygiellidae Stygiella Stygiella-incarcerata
OQY25878.1 Bacteria Candidatus-Cloacimonetes Candidatus-Cloacimonetes-bacterium-4572 55
WP 092349000.1 Bacteria Proteobacteria delta epsilon-subdivisions Deltaproteobacteria Desulfuromonadales Desulfuromonadaceae Desulfuromusa Desulfuromusa-kysingii
PMP69712.1 Bacteria Deferribacteres Deferribacteres Deferribacterales Deferribacteraceae Calditerrivibrio Calditerrivibrio-nitroreducens
WP 013452006.1 Bacteria Deferribacteres Deferribacteres Deferribacterales Deferribacteraceae Calditerrivibrio Calditerrivibrio-nitroreducens
WP 013008386.1 Bacteria Deferribacteres Deferribacteres Deferribacterales Deferribacteraceae Deferribacter Deferribacter-desulfuricans
WP 011188791.1 Bacteria Proteobacteria delta epsilon-subdivisions Deltaproteobacteria Desulfobacterales Desulfobulbaceae Desulfotalea Desulfotalea-psychrophila
WP 014959069.1 Bacteria Proteobacteria delta epsilon-subdivisions Deltaproteobacteria Desulfobacterales Desulfobacteraceae Desulfobacula Desulfobacula-toluolica
WP 092235246.1 Bacteria Proteobacteria delta epsilon-subdivisions Deltaproteobacteria Desulfobacterales Desulfobacteraceae Desulfobacula Desulfobacula-phenolica
WP 084070319.1 Bacteria Proteobacteria delta epsilon-subdivisions Deltaproteobacteria Desulfobacterales Desulfobacteraceae Desulfobacterium Desulfobacterium-vacuolatum
BMAN M0811_10279
BMAN M0811_13065
BMAN M0811_13066
BUSSELTON M0812_25340
BUSSELTON M0812_29005
SCHOONER M0813_02396
BUSSELTON M0812_26408
SCHOONER M0813_22418
SCHOONER M0813_24707
BUSSELTON M0812_08587
BUSSELTON M0812_29051
BUSSELTON M0812_29052
SCHOONER M0813_17779
SCHOONER M0813_09270
0.5

Figure S17L Phylogenetic reconstruction of NADH oxidase. Alignment with MAFFT, site selection BMGE, and phylogeny IQTree model C20+G4,1,000 ultrafast bootstrap. *Anaeramoeba* sequences are in red, eukaryotic sequences in blue, and prokaryotic sequences in bright green.

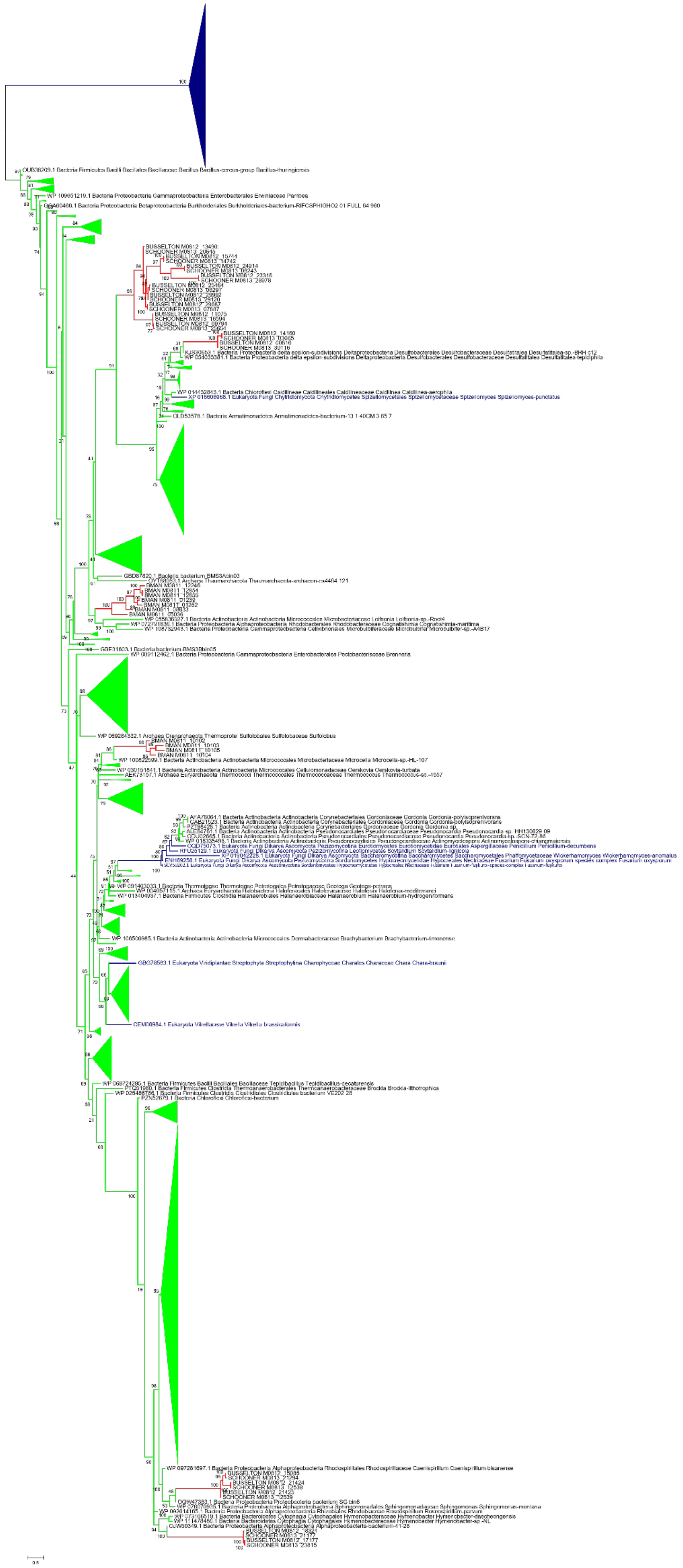

OUB36209.1 Bacteria Firmicutes Bacilli Bacillales Bacillaceae Bacillus Bacillus-cereus-group Bacillus-thuringiensis
WP 100651219.1 Bacteria Proteobacteria Gammaproteobacteria Enterobacterales Erwiniaceae Pantoea
GBD87820.1 Bacteria bacterium BMS3Abin03
GBE31603.1 Bacteria bacterium BMS3Bbin05
WP 069284332.1 Archaea Crenarchaeota Thermoprotei Sulfolobales Sulfolobaceae Sulfolobus
GBG78583.1 Eukaryota Viridiplantae Streptophyta Streptophytina Charophyceae Charales Characeae Chara Chara-braunii
CEM08984.1 Eukaryota Vitrellaceae Vitrella Vitrella brassicaformis
WP 097281697.1 Bacteria Proteobacteria Alphaproteobacteria Rhodospirillales Rhodospirillaceae Caenispirillum Caenispirillum bisanense
0.5

Figure S17M Phylogenetic reconstruction of OsmC. Alignment with MAFFT, site selection BMGE and phylogeny IQTree model LG4X 1000 ultrafast bootstrap. *Anaeramoeba* sequences are in red, Eukaryotic sequences in blue and prokaryotic sequences in bright green.

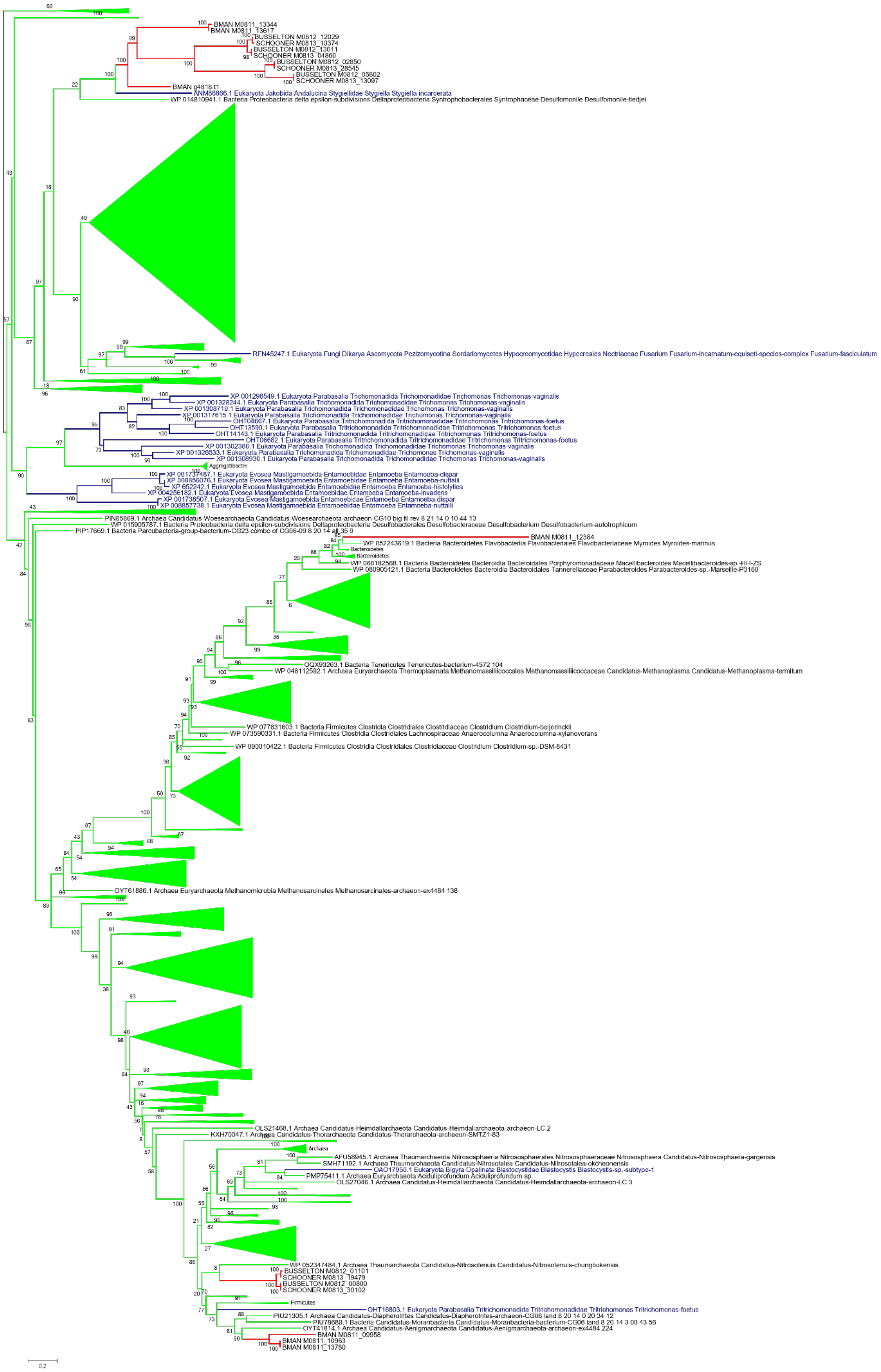
BMAN M0811_13344
BMAN M0811_13617
BUSSELTON M0812_12029
SCHOONER M0813_10374
BUSSELTON M0812_13011
SCHOONER M0813_04860
BUSSELTON M0812_02850
SCHOONER M0813_28545
BUSSELTON M0812_05802
SCHOONER M0813_13097
BMAN g4818.t1
ANM86866.1 Eukaryota Jakobida Andalucina Stygiellidae Stygiella Stygiella-incarcerata
WP 014810941.1 Bacteria Proteobacteria delta epsilon-subdivisions Deltaproteobacteria Syntrophobacterales Syntrophaceae Desulfomonile Desulfomonile-tiedjei
RFN45247.1 Eukaryota Fungi Dikarya Ascomycota Pezizomycotina Sordariomycetes Hypocreomycetidae Hypocreales Nectriaceae Fusarium Fusarium-incarnatum-equiseti-species-complex Fusarium-fasciculatum
XP 001298549.1 Eukaryota Parabasalia Trichomonadida Trichomonadidae Trichomonas Trichomonas-vaginalis
XP 001328244.1 Eukaryota Parabasalia Trichomonadida Trichomonadidae Trichomonas Trichomonas-vaginalis
XP 001308719.1 Eukaryota Parabasalia Trichomonadida Trichomonadidae Trichomonas Trichomonas-vaginalis
XP 001317615.1 Eukaryota Parabasalia Trichomonadida Trichomonadidae Trichomonas Trichomonas-vaginalis
OHT04667.1 Eukaryota Parabasalia Tritrichomonadida Tritrichomonadidae Tritrichomonas Tritrichomonas-foetus
OHT13590.1 Eukaryota Parabasalia Tritrichomonadida Tritrichomonadidae Tritrichomonas Tritrichomonas-foetus
OHT14143.1 Eukaryota Parabasalia Tritrichomonadida Tritrichomonadidae Tritrichomonas Tritrichomonas-foetus
OHT06682.1 Eukaryota Parabasalia Tritrichomonadida Tritrichomonadidae Tritrichomonas Tritrichomonas-foetus
XP 001302386.1 Eukaryota Parabasalia Trichomonadida Trichomonadidae Trichomonas Trichomonas-vaginalis
XP 001326533.1 Eukaryota Parabasalia Trichomonadida Trichomonadidae Trichomonas Trichomonas-vaginalis
XP 001308930.1 Eukaryota Parabasalia Trichomonadida Trichomonadidae Trichomonas Trichomonas-vaginalis
Aggregatibacter
XP 001737487.1 Eukaryota Evosea Mastigamoebida Entamoebidae Entamoeba Entamoeba-dispar
XP 008856076.1 Eukaryota Evosea Mastigamoebida Entamoebidae Entamoeba Entamoeba-nuttalli
XP 652242.1 Eukaryota Evosea Mastigamoebida Entamoebidae Entamoeba Entamoeba-histolytica
XP 004256182.1 Eukaryota Evosea Mastigamoebida Entamoebidae Entamoeba Entamoeba-invadens
XP 001738507.1 Eukaryota Evosea Mastigamoebida Entamoebidae Entamoeba Entamoeba-dispar
XP 008857738.1 Eukaryota Evosea Mastigamoebida Entamoebidae Entamoeba Entamoeba-nuttalli
PIN85869.1 Archaea Candidatus Woesearchaeota Candidatus Woesearchaeota archaeon CG10 big fil rev 8 21 14 0 10 44 13
WP 015905787.1 Bacteria Proteobacteria delta epsilon-subdivisions Deltaproteobacteria Desulfobacterales Desulfobacteraceae Desulfobacterium Desulfobacterium-autotrophicum
PIP17669.1 Bacteria Parcubacteria-group-bacterium-CG23 combo of CG06-09 8 20 14 all 35 9
BMAN M0811_12384
WP 052243619.1 Bacteria Bacteroidetes Flavobacteriia Flavobacteriales Flavobacteriaceae Myroides Myroides-marinus
Bacteroidetes
Bacteroidetes
WP 068182568.1 Bacteria Bacteroidetes Bacteroidia Bacteroidales Porphyromonadaceae Macellibacteroides Macellibacteroides-sp.-HH-ZS
WP 080905121.1 Bacteria Bacteroidetes Bacteroidia Bacteroidales Tannerellaceae Parabacteroides Parabacteroides-sp.-Marseille-P3160
OQX93263.1 Bacteria Tenericutes Tenericutes-bacterium-4572 104
WP 048112592.1 Archaea Euryarchaeota Thermoplasmata Methanomassiliicoccales Methanomassiliicoccaceae Candidatus-Methanoplasma Candidatus-Methanoplasma-termitum
WP 077831603.1 Bacteria Firmicutes Clostridia Clostridiales Clostridiaceae Clostridium Clostridium-beijerinckii
WP 073590331.1 Bacteria Firmicutes Clostridia Clostridiales Lachnospiraceae Anaerocolumna Anaerocolumna-xylanovorans
WP 090010422.1 Bacteria Firmicutes Clostridia Clostridiales Clostridiaceae Clostridium Clostridium-sp.-DSM-8431
OYT61886.1 Archaea Euryarchaeota Methanomicrobia Methanosarcinales Methanosarcinales-archaeon-ex4484 138
OLS21468.1 Archaea Candidatus-Heimdallarchaeota Candidatus-Heimdallarchaeota-archaeon-LC 2
KXH70047.1 Archaea Candidatus-Thorarchaeota Candidatus-Thorarchaeota-archaeon-SMTZ1-83
Archaea
AFU58945.1 Archaea Thaumarchaeota Nitrososphaeria Nitrososphaerales Nitrososphaeraceae Nitrososphaera Candidatus-Nitrososphaera-gargensis
SMH71192.1 Archaea Thaumarchaeota Candidatus-Nitrosotalea Candidatus-Nitrosotalea-okcheonensis
OAO17950.1 Eukaryota Bigyra Opalinata Blastocystidae Blastocystis Blastocystis-sp.-subtype-1
PMP75411.1 Archaea Euryarchaeota Aciduliprofundum Aciduliprofundum-sp.
OLS27046.1 Archaea Candidatus-Heimdallarchaeota Candidatus-Heimdallarchaeota-archaeon-LC 3
WP 052347484.1 Archaea Thaumarchaeota Candidatus-Nitrosotenuis Candidatus-Nitrosotenuis-chungbukensis
BUSSELTON M0812_01101
SCHOONER M0813_19479
BUSSELTON M0812_00800
SCHOONER M0813_30102
Firmicutes
OHT16803.1 Eukaryota Parabasalia Tritrichomonadida Tritrichomonadidae Tritrichomonas Tritrichomonas-foetus
PIU21305.1 Archaea Candidatus-Diapherotrites Candidatus-Diapherotrites-archaeon-CG08 land 8 20 14 0 20 34 12
PIU78689.1 Bacteria Candidatus-Moranbacteria Candidatus-Moranbacteria-bacterium-CG06 land 8 20 14 3 00 43 56
OYT41814.1 Archaea Candidatus-Aenigmarchaeota Candidatus-Aenigmarchaeota-archaeon-ex4484 224
BMAN M0811_09958
BMAN M0811_10963
BMAN M0811_13780
0.2

Figure S17N Phylogenetic reconstruction of Nitroreductases. Alignment with MAFFT, site selection BMGE and phylogeny IQTree model LG4X 1000 ultrafast bootstrap. *Anaeramoeba* sequences are in red, Eukaryotic sequences in blue and prokaryotic sequences in bright green.

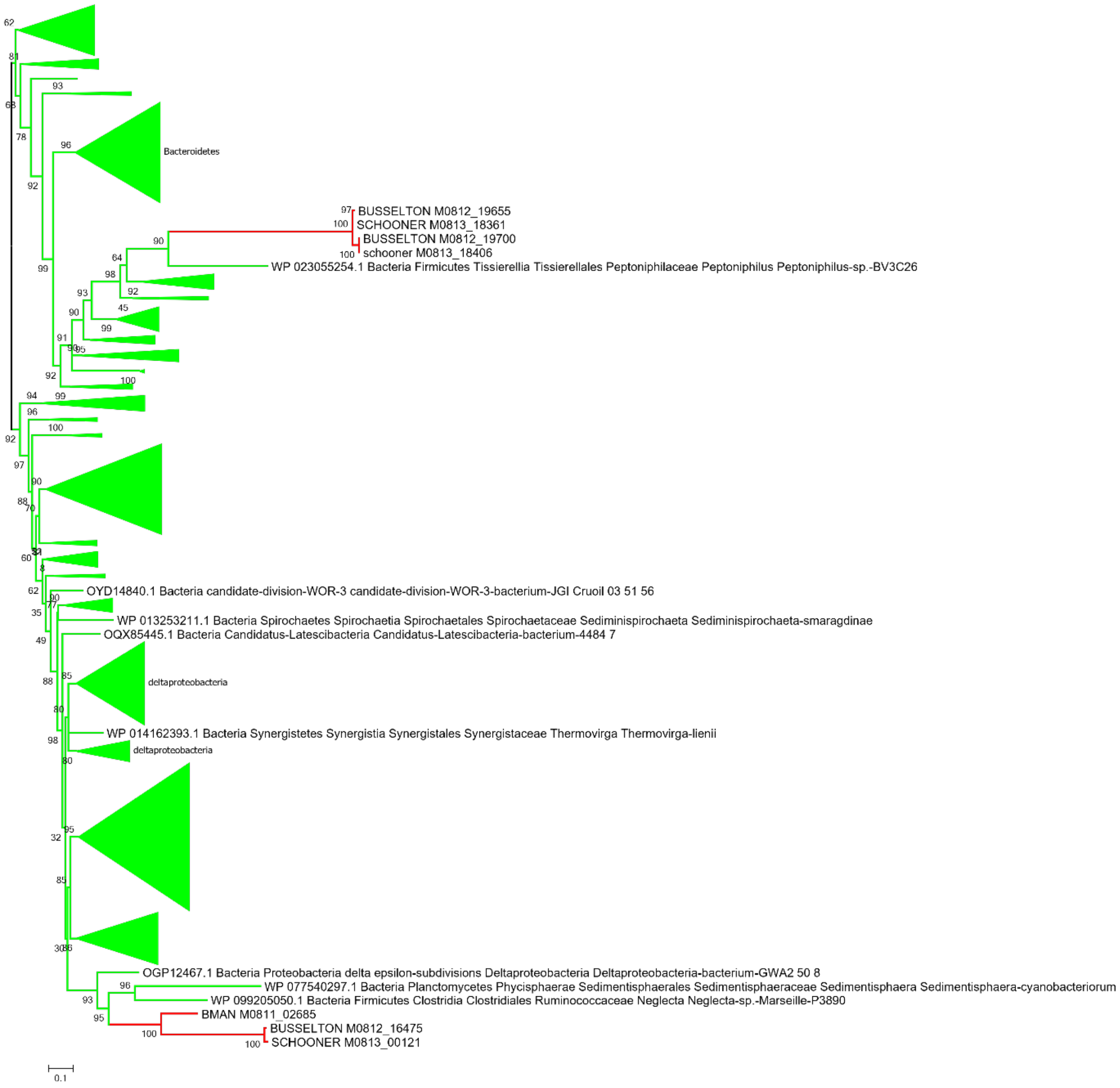

Figure S17O Phylogenetic reconstruction of Rubrerythrin. Alignment with MAFFT, site selection BMGE and phylogeny IQTree model LG4X 1000 ultrafast bootstrap. *Anaeramoeba* sequences are in red and prokaryotic sequences in bright green.

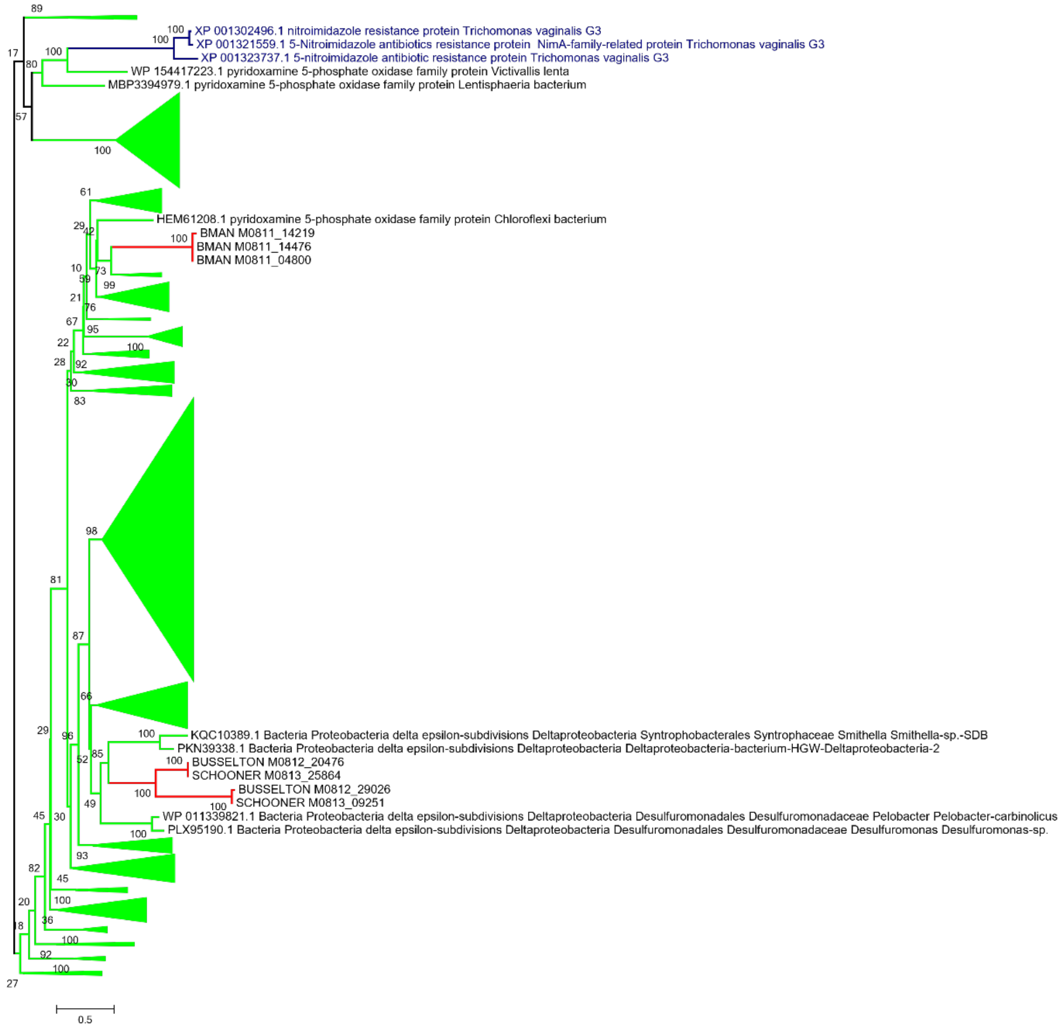

Figure S17P Phylogenetic reconstruction of 5-Nitroimidazole reductases. Alignment with MAFFT, selection of sites with less than 30% gaps, and phylogeny IQtree model LG4X 1000 ultrafast bootstrap. *Anaeramoeba* sequences are in red, eukaryotic sequences in blue, and prokaryotic sequences in bright green.

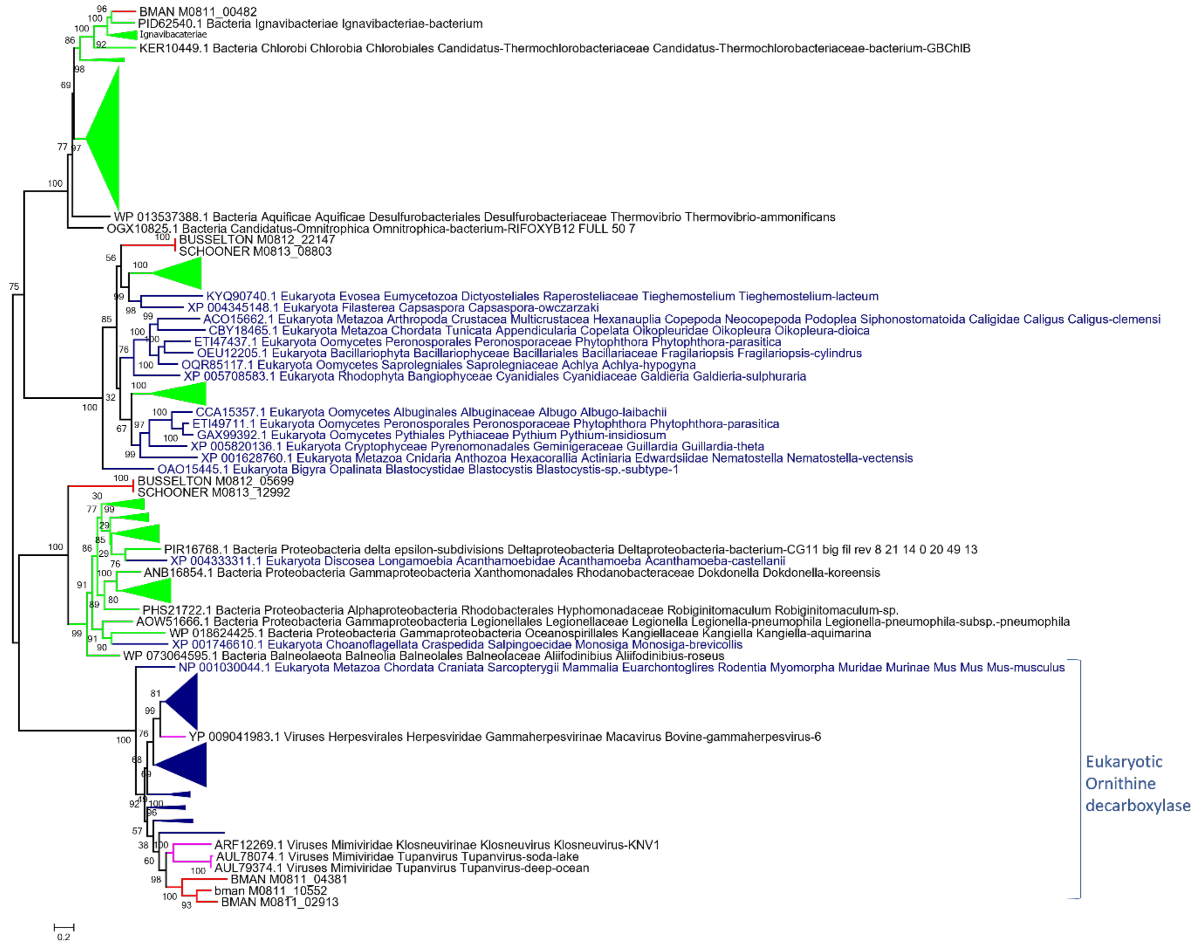

Figure S17Q Phylogenetic reconstruction of Diaminopimelate decarboxylase. Alignment with MAFFT, site selection BMGE and phylogeny IQtree model LG4X 1000 ultrafast bootstrap. *Anaeramoeba* sequences are in red, eukaryotic sequences in blue, viral sequences in fushia, and prokaryotic sequences in bright green.

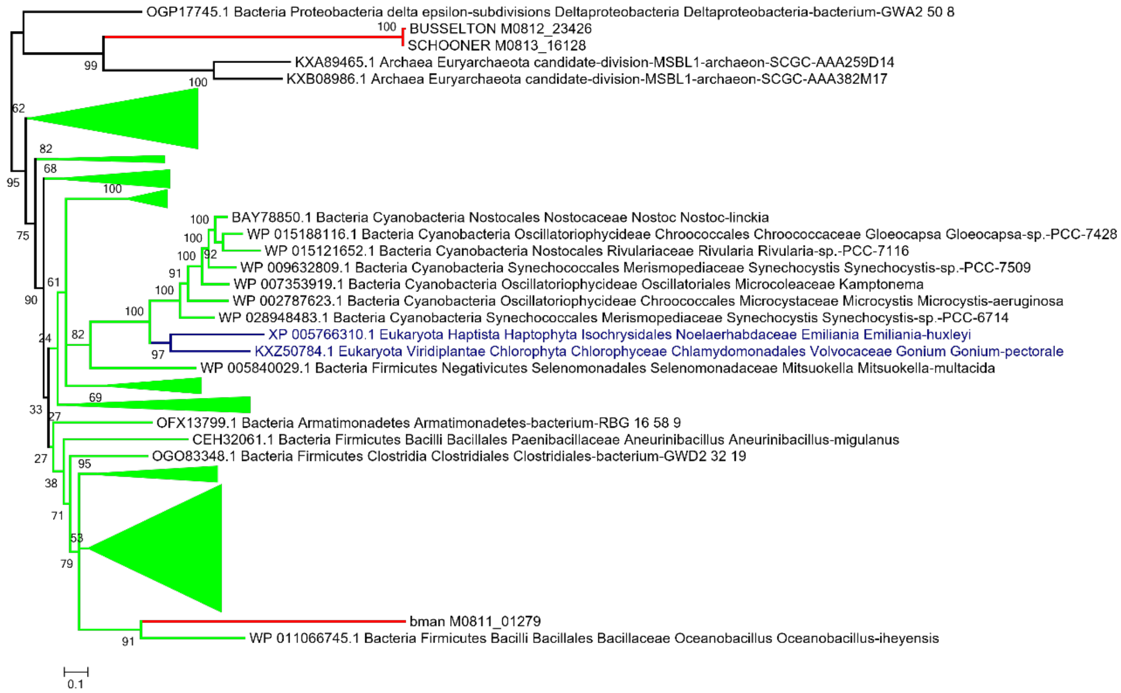

Figure S17R Phylogenetic reconstruction of Diaminopimelate epimerase. Alignment with MAFFT, site selection BMGE and phylogeny IQtree model LG4X 1000 ultrafast bootstrap. *Anaeramoeba* sequences are in red, eukaryotic sequences in blue, and prokaryotic sequences in bright green.

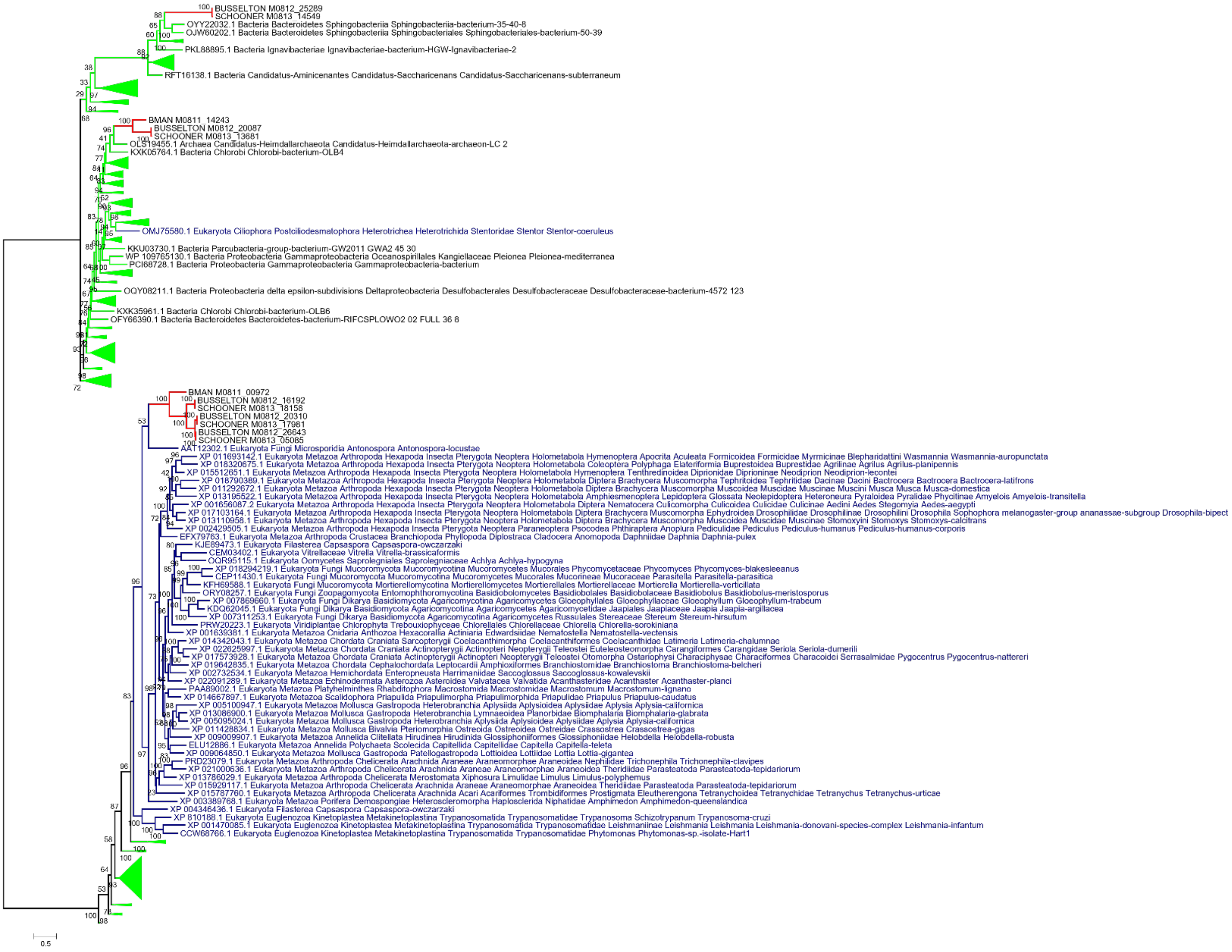

Figure S17S Phylogenetic reconstruction of Saccharopine dehydrogenase. Alignment with MAFFT, selection of sites with less than 30% gaps and phylogeny IQtree model C20+G4 1000 ultrafast bootstrap. *Anaeramoeba* sequences are in red, eukaryotic sequences in blue, and prokaryotic sequences in bright green.

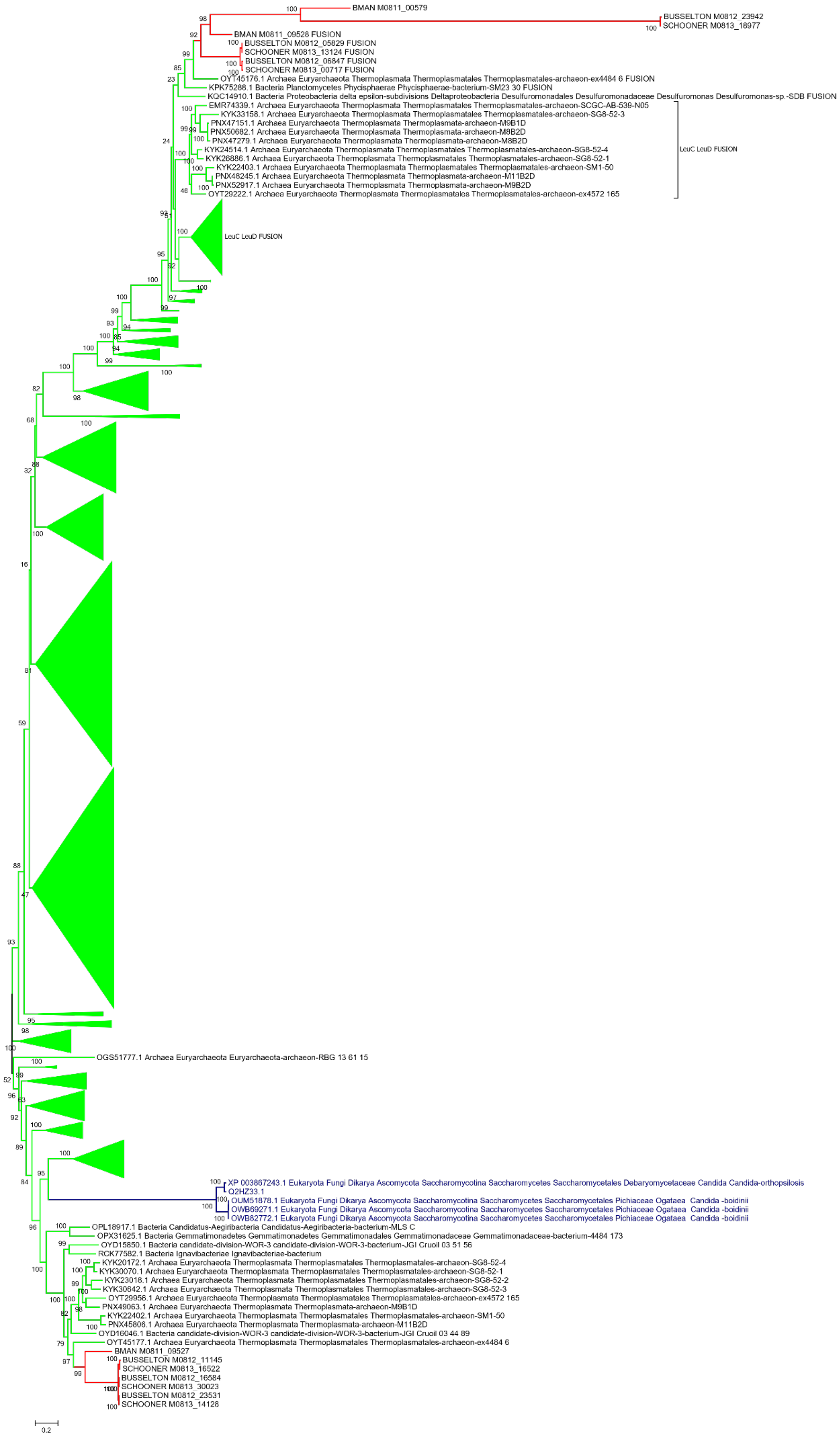

BMAN M0811_00579
BUSSELTON M0812_23942
SCHOONER M0813_18977
BMAN M0811_09528 FUSION
BUSSELTON M0812_05829 FUSION
SCHOONER M0813_13124 FUSION
BUSSELTON M0812_06847 FUSION
SCHOONER M0813_00717 FUSION
OYT45176.1 Archaea Euryarchaeota Thermoplasmata Thermoplasmatales Thermoplasmatales-archaeon-ex4484 6 FUSION
KPK75288.1 Bacteria Planctomycetes Phycisphaerae Phycisphaerae-bacterium-SM23 30 FUSION
KQC14910.1 Bacteria Proteobacteria delta epsilon-subdivisions Deltaproteobacteria Desulfuromonadales Desulfuromonadaceae Desulfuromonas Desulfuromonas-sp.-SDB FUSION
EMR74339.1 Archaea Euryarchaeota Thermoplasmata Thermoplasmatales Thermoplasmatales-archaeon-SCGC-AB-539-N05
KYK33158.1 Archaea Euryarchaeota Thermoplasmata Thermoplasmatales Thermoplasmatales-archaeon-SG8-52-3
PNX47151.1 Archaea Euryarchaeota Thermoplasmata Thermoplasmata-archaeon-M9B1D
PNX50682.1 Archaea Euryarchaeota Thermoplasmata Thermoplasmata-archaeon-M8B2D
PNX47279.1 Archaea Euryarchaeota Thermoplasmata Thermoplasmata-archaeon-M8B2D
KYK24514.1 Archaea Euryarchaeota Thermoplasmata Thermoplasmatales Thermoplasmatales-archaeon-SG8-52-4
KYK26886.1 Archaea Euryarchaeota Thermoplasmata Thermoplasmatales Thermoplasmatales-archaeon-SG8-52-1
KYK22403.1 Archaea Euryarchaeota Thermoplasmata Thermoplasmatales Thermoplasmatales-archaeon-SM1-50
PNX48245.1 Archaea Euryarchaeota Thermoplasmata Thermoplasmata-archaeon-M11B2D
PNX52917.1 Archaea Euryarchaeota Thermoplasmata Thermoplasmata-archaeon-M9B2D
OYT29222.1 Archaea Euryarchaeota Thermoplasmata Thermoplasmatales Thermoplasmatales-archaeon-ex4572 165
LeuC LeuD FUSION
LeuC LeuD FUSION
OGS51777.1 Archaea Euryarchaeota Euryarchaeota-archaeon-RBG 13 61 15
XP 003867243.1 Eukaryota Fungi Dikarya Ascomycota Saccharomycotina Saccharomycetes Saccharomycetales Debaryomycetaceae Candida Candida-orthopsilosis
Q2HZ33.1
OUM51878.1 Eukaryota Fungi Dikarya Ascomycota Saccharomycotina Saccharomycetes Saccharomycetales Pichiaceae Ogataea Candida -boidinii
OWB69271.1 Eukaryota Fungi Dikarya Ascomycota Saccharomycotina Saccharomycetes Saccharomycetales Pichiaceae Ogataea Candida -boidinii
OWB82772.1 Eukaryota Fungi Dikarya Ascomycota Saccharomycotina Saccharomycetes Saccharomycetales Pichiaceae Ogataea Candida -boidinii
OPL18917.1 Bacteria Candidatus-Aegiribacteria Candidatus-Aegiribacteria-bacterium-MLS C
OPX31625.1 Bacteria Gemmatimonadetes Gemmatimonadetes Gemmatimonadales Gemmatimonadaceae Gemmatimonadaceae-bacterium-4484 173
OYD15850.1 Bacteria candidate-division-WOR-3 candidate-division-WOR-3-bacterium-JGI Cruoil 03 51 56
RCK77582.1 Bacteria Ignavibacteriae Ignavibacteriae-bacterium
KYK20172.1 Archaea Euryarchaeota Thermoplasmata Thermoplasmatales Thermoplasmatales-archaeon-SG8-52-4
KYK30070.1 Archaea Euryarchaeota Thermoplasmata Thermoplasmatales Thermoplasmatales-archaeon-SG8-52-1
KYK23018.1 Archaea Euryarchaeota Thermoplasmata Thermoplasmatales Thermoplasmatales-archaeon-SG8-52-2
KYK30642.1 Archaea Euryarchaeota Thermoplasmata Thermoplasmatales Thermoplasmatales-archaeon-SG8-52-3
OYT29956.1 Archaea Euryarchaeota Thermoplasmata Thermoplasmatales Thermoplasmatales-archaeon-ex4572 165
PNX49063.1 Archaea Euryarchaeota Thermoplasmata Thermoplasmata-archaeon-M9B1D
KYK22402.1 Archaea Euryarchaeota Thermoplasmata Thermoplasmatales Thermoplasmatales-archaeon-SM1-50
PNX45806.1 Archaea Euryarchaeota Thermoplasmata Thermoplasmata-archaeon-M11B2D
OYD16046.1 Bacteria candidate-division-WOR-3 candidate-division-WOR-3-bacterium-JGI Cruoil 03 44 89
OYT45177.1 Archaea Euryarchaeota Thermoplasmata Thermoplasmatales Thermoplasmatales-archaeon-ex4484 6
BMAN M0811_09527
BUSSELTON M0812_11145
SCHOONER M0813_16522
BUSSELTON M0812_16584
SCHOONER M0813_30023
BUSSELTON M0812_23531
SCHOONER M0813_14128
0.2

Figure S17T Phylogenetic reconstruction of LeuC. Alignment with MAFFT, site selection BMGE and phylogeny IQtree model LG4X 1000 ultrafast bootstrap. *Anaeramoeba* sequences are in red, eukaryotic sequences in blue, and prokaryotic sequences in bright green.

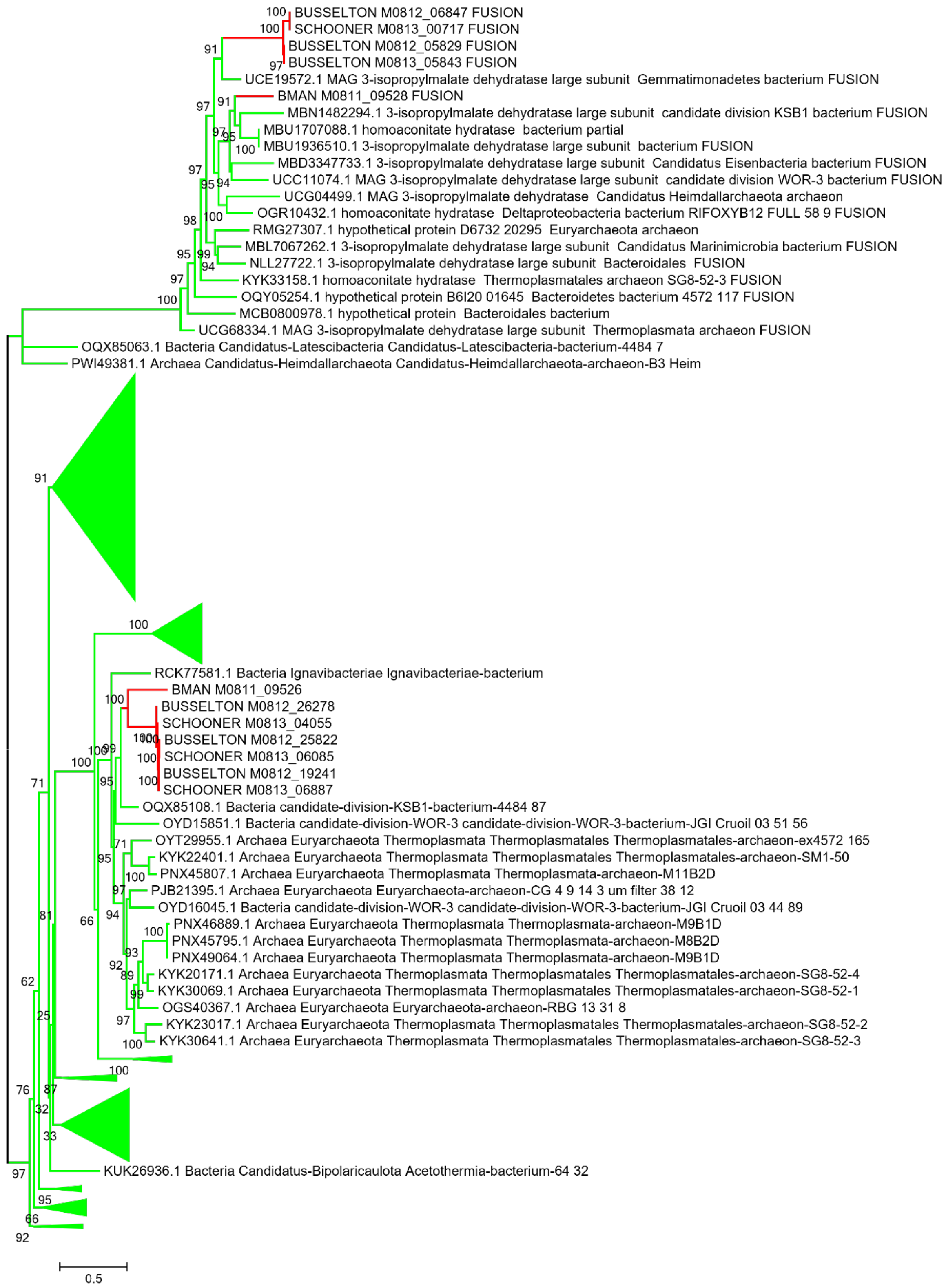

Figure S17U Phylogenetic reconstruction of LeuD. Alignment with Muscle, site selection BMGE and phylogeny IQtree model LG4X 1000 ultrafast bootstrap. *Anaeramoeba* sequences are in red, eukaryotic sequences in blue, and prokaryotic sequences in bright green.

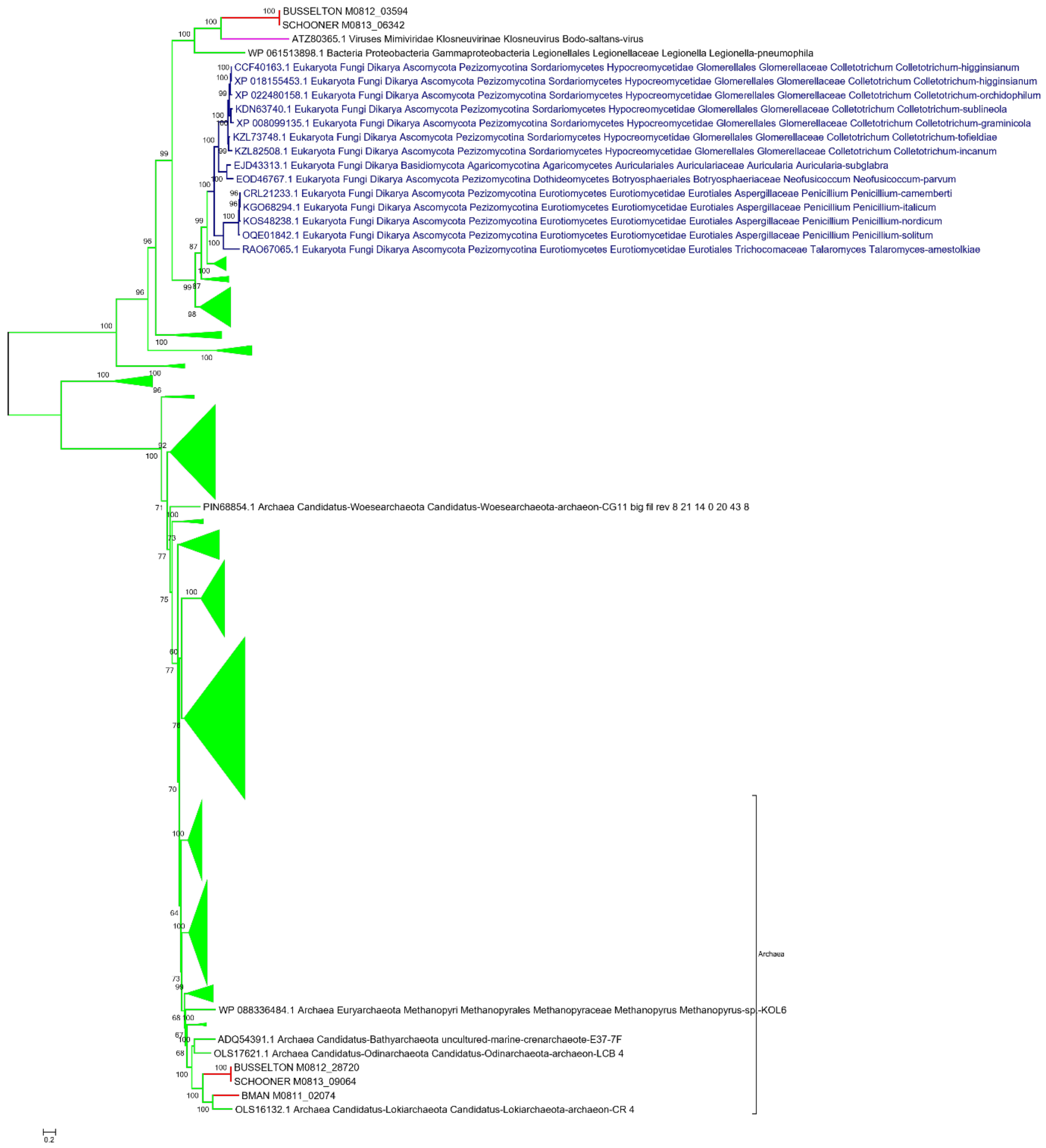

Figure S17V Phylogenetic reconstruction of FAD-dependent dehydrogenase. Alignment with MAFFT, site selection with BMGE and phylogeny with IQtree model LG4X with 1000 ultrafast bootstrap. *Anaeramoeba* sequences are in red, eukaryotic sequences in blue, viral sequences in light purple and prokaryotic sequences in bright green.

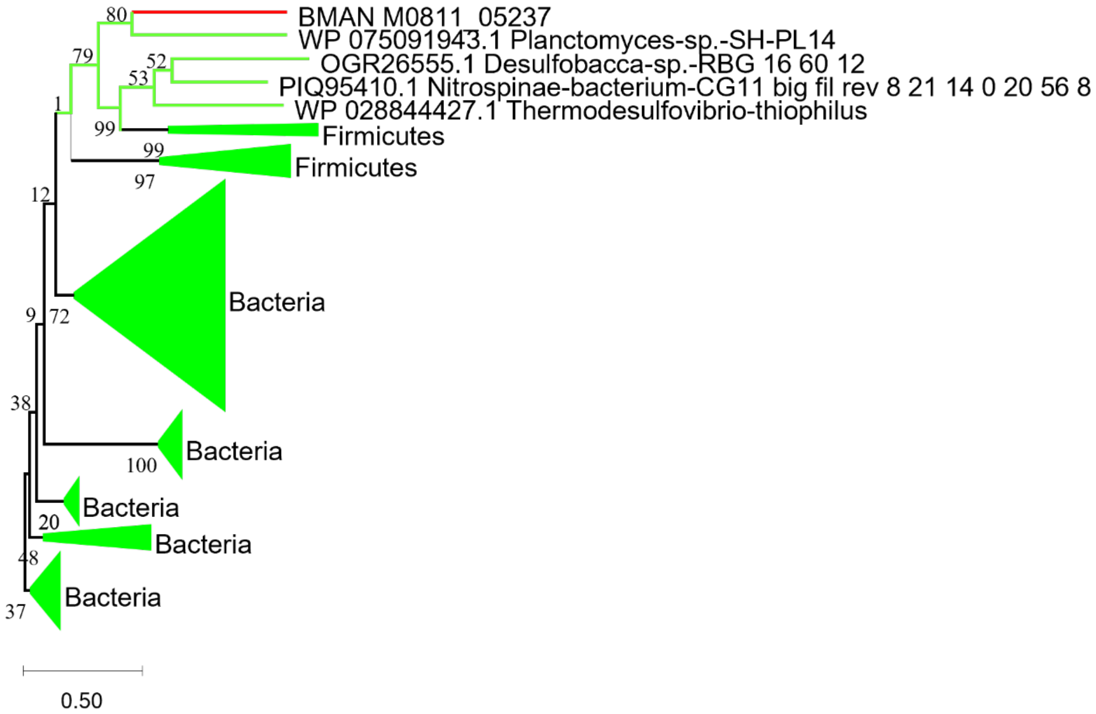

Figure S17W Phylogenetic reconstruction of CobC. Alignment with MAFFT, site selection BMGE and phylogeny IQtree model LG4X 1000 ultrafast bootstrap. *Anaeramoeba* sequences are in red, and prokaryotic sequences in bright green.

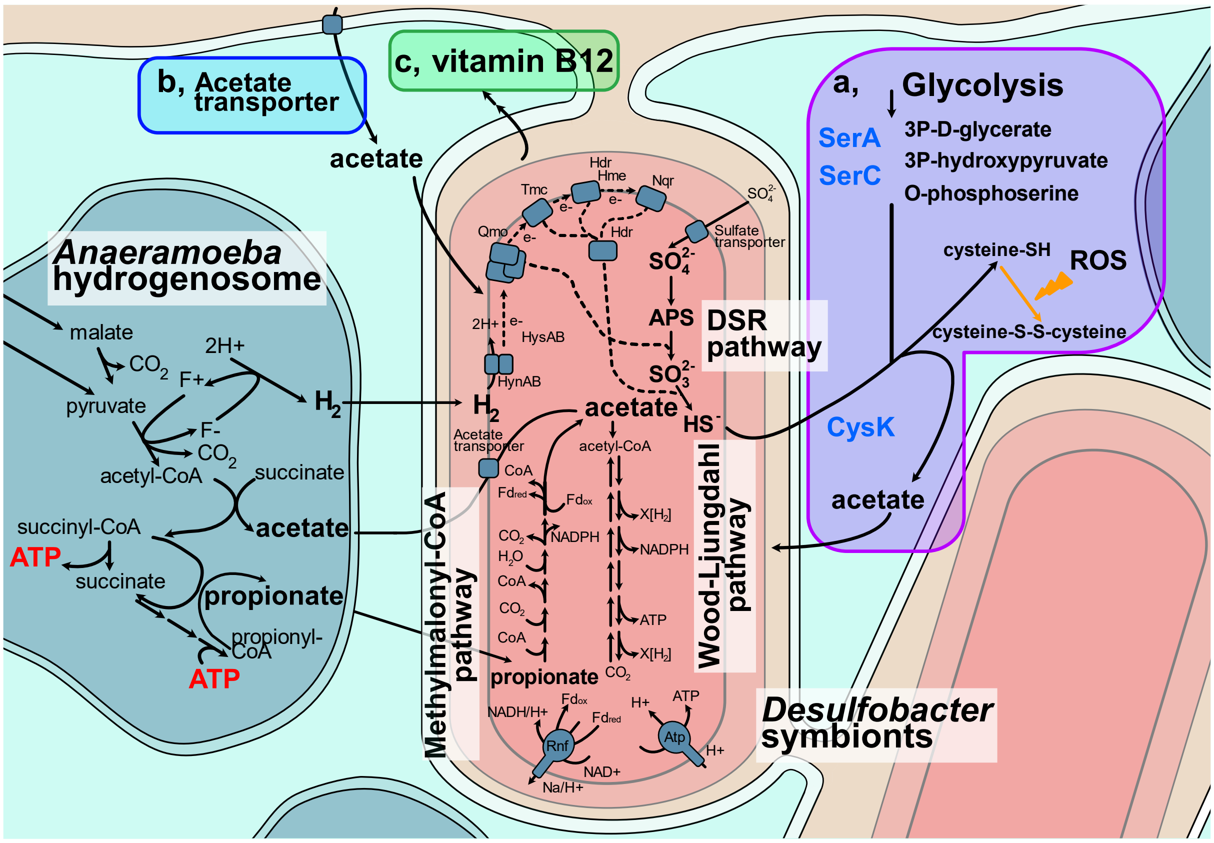

Figure S18: *Anaeramoeba* uses ancestral and LGT-derived enzymes to interact with its symbionts. Suggested syntrophic interactions between *Anaeramoeba* hydrogenosomes (blue) and symbionts (pink/salmon) based on metabolic reconstruction from transcriptomic and genomic evidence. The host's ATP-producing hydrogenosomes generate H_2, acetate, and propionate as end-products. Based on metatranscriptomic data, the symbionts use the products of the hydrogenosomes by prominently expressing the dissimilatory sulfate reduction (DSR), methylmalonyl-CoA, and the Wood-Ljungdahl pathways. The symbionts are in deep membrane-pits with a connection to the cell surrounding that gives ready access to sulfate (gold). **a,** A hybrid pathway to reuse SH^- is formed by the ancestral SerC and the LGTs CysK and SerA (purple). The pathway would recycle acetate for the host or symbiont and provide reduced cysteine for mitigating ROS. **b,** The host has laterally transferred acetate transporters that might help to provide acetate to the symbionts (light blue). The actual localization and orientation of the acetate transporters are unknown, and we only show a suggested localization. **c,** Vitamin B12 is produced by the symbionts and might be transferred to the host that requires it as a cofactor for several enzymes (green). *A. ignava* BMAN has CobC, the final enzyme of the vitamin B12 synthesis pathway as an LGT.

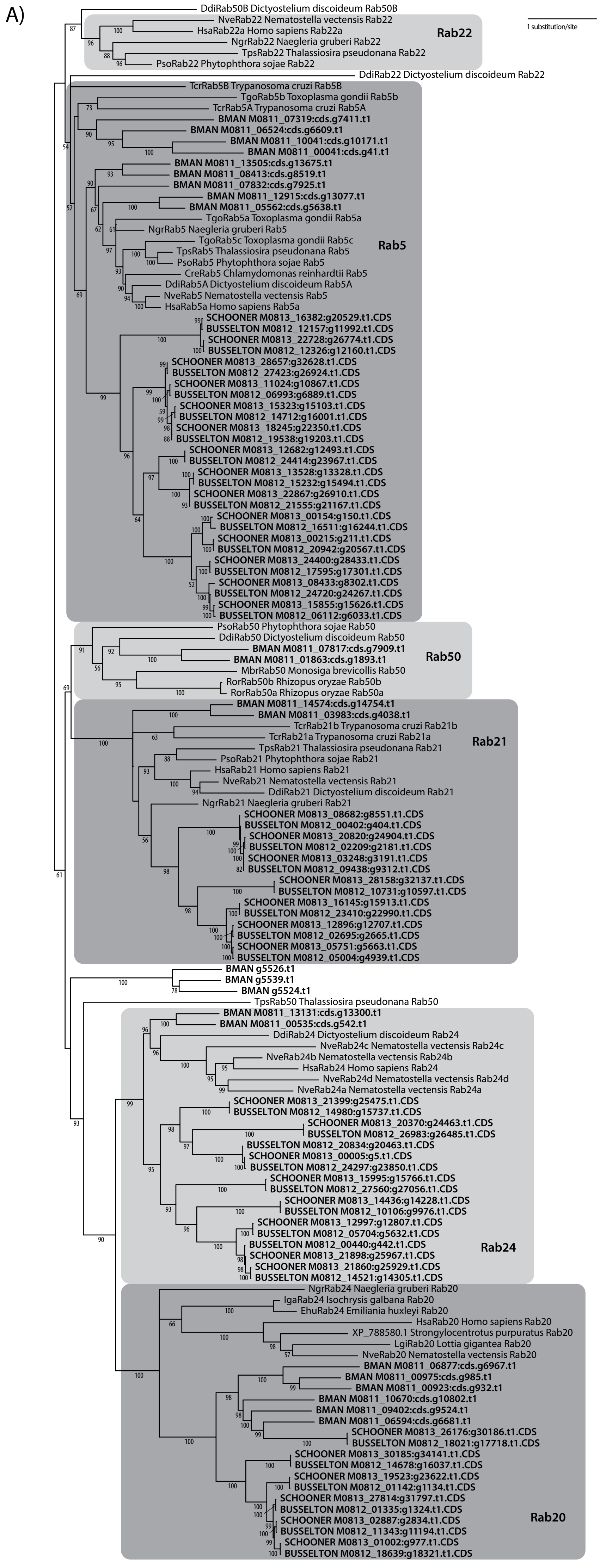
A)
1 substitution/site
DdiRab50B Dictyostelium discoideum Rab50B
NveRab22 Nematostella vectensis Rab22
HsaRab22a Homo sapiens Rab22a
NgrRab22 Naegleria gruberi Rab22
TpsRab22 Thalassiosira pseudonana Rab22
PsoRab22 Phytophthora sojae Rab22
Rab22
DdiRab22 Dictyostelium discoideum Rab22
TcrRab5B Trypanosoma cruzi Rab5B
TgoRab5b Toxoplasma gondii Rab5b
TcrRab5A Trypanosoma cruzi Rab5A
BMAN M0811_07319:cds.g7411.t1
BMAN M0811_06524:cds.g6609.t1
BMAN M0811_10041:cds.g10171.t1
BMAN M0811_00041:cds.g41.t1
BMAN M0811_13505:cds.g13675.t1
BMAN M0811_08413:cds.g8519.t1
BMAN M0811_07832:cds.g7925.t1
BMAN M0811_12915:cds.g13077.t1
BMAN M0811_05562:cds.g5638.t1
TgoRab5a Toxoplasma gondii Rab5a
NgrRab5 Naegleria gruberi Rab5
TgoRab5c Toxoplasma gondii Rab5c
TpsRab5 Thalassiosira pseudonana Rab5
PsoRab5 Phytophthora sojae Rab5
CreRab5 Chlamydomonas reinhardtii Rab5
DdiRab5A Dictyostelium discoideum Rab5A
NveRab5 Nematostella vectensis Rab5
HsaRab5a Homo sapiens Rab5a
Rab5
SCHOONER M0813_16382:g20529.t1.CDS
BUSSELTON M0812_12157:g11992.t1.CDS
SCHOONER M0813_22728:g26774.t1.CDS
BUSSELTON M0812_12326:g12160.t1.CDS
SCHOONER M0813_28657:g32628.t1.CDS
BUSSELTON M0812_27423:g26924.t1.CDS
SCHOONER M0813_11024:g10867.t1.CDS
BUSSELTON M0812_06993:g6889.t1.CDS
SCHOONER M0813_15323:g15103.t1.CDS
BUSSELTON M0812_14712:g16001.t1.CDS
SCHOONER M0813_18245:g22350.t1.CDS
BUSSELTON M0812_19538:g19203.t1.CDS
SCHOONER M0813_12682:g12493.t1.CDS
BUSSELTON M0812_24414:g23967.t1.CDS
SCHOONER M0813_13528:g13328.t1.CDS
BUSSELTON M0812_15232:g15494.t1.CDS
SCHOONER M0813_22867:g26910.t1.CDS
BUSSELTON M0812_21555:g21167.t1.CDS
SCHOONER M0813_00154:g150.t1.CDS
BUSSELTON M0812_16511:g16244.t1.CDS
SCHOONER M0813_00215:g211.t1.CDS
BUSSELTON M0812_20942:g20567.t1.CDS
SCHOONER M0813_24400:g28433.t1.CDS
BUSSELTON M0812_17595:g17301.t1.CDS
SCHOONER M0813_08433:g8302.t1.CDS
BUSSELTON M0812_24720:g24267.t1.CDS
SCHOONER M0813_15855:g15626.t1.CDS
BUSSELTON M0812_06112:g6033.t1.CDS
PsoRab50 Phytophthora sojae Rab50
DdiRab50 Dictyostelium discoideum Rab50
BMAN M0811_07817:cds.g7909.t1
BMAN M0811_01863:cds.g1893.t1
MbrRab50 Monosiga brevicollis Rab50
RorRab50b Rhizopus oryzae Rab50b
RorRab50a Rhizopus oryzae Rab50a
Rab50
BMAN M0811_14574:cds.g14754.t1
BMAN M0811_03983:cds.g4038.t1
TcrRab21b Trypanosoma cruzi Rab21b
TcrRab21a Trypanosoma cruzi Rab21a
TpsRab21 Thalassiosira pseudonana Rab21
PsoRab21 Phytophthora sojae Rab21
HsaRab21 Homo sapiens Rab21
NveRab21 Nematostella vectensis Rab21
DdiRab21 Dictyostelium discoideum Rab21
NgrRab21 Naegleria gruberi Rab21
Rab21
SCHOONER M0813_08682:g8551.t1.CDS
BUSSELTON M0812_00402:g404.t1.CDS
SCHOONER M0813_20820:g24904.t1.CDS
BUSSELTON M0812_02209:g2181.t1.CDS
SCHOONER M0813_03248:g3191.t1.CDS
BUSSELTON M0812_09438:g9312.t1.CDS
SCHOONER M0813_28158:g32137.t1.CDS
BUSSELTON M0812_10731:g10597.t1.CDS
SCHOONER M0813_16145:g15913.t1.CDS
BUSSELTON M0812_23410:g22990.t1.CDS
SCHOONER M0813_12896:g12707.t1.CDS
BUSSELTON M0812_02695:g2665.t1.CDS
SCHOONER M0813_05751:g5663.t1.CDS
BUSSELTON M0812_05004:g4939.t1.CDS
BMAN g5526.t1
BMAN g5539.t1
BMAN g5524.t1
TpsRab50 Thalassiosira pseudonana Rab50
BMAN M0811_13131:cds.g13300.t1
BMAN M0811_00535:cds.g542.t1
DdiRab24 Dictyostelium discoideum Rab24
NveRab24c Nematostella vectensis Rab24c
NveRab24b Nematostella vectensis Rab24b
HsaRab24 Homo sapiens Rab24
NveRab24d Nematostella vectensis Rab24d
NveRab24a Nematostella vectensis Rab24a
SCHOONER M0813_21399:g25475.t1.CDS
BUSSELTON M0812_14980:g15737.t1.CDS
SCHOONER M0813_20370:g24463.t1.CDS
BUSSELTON M0812_26983:g26485.t1.CDS
BUSSELTON M0812_20834:g20463.t1.CDS
SCHOONER M0813_00005:g5.t1.CDS
BUSSELTON M0812_24297:g23850.t1.CDS
SCHOONER M0813_15995:g15766.t1.CDS
BUSSELTON M0812_27560:g27056.t1.CDS
SCHOONER M0813_14436:g14228.t1.CDS
BUSSELTON M0812_10106:g9976.t1.CDS
SCHOONER M0813_12997:g12807.t1.CDS
BUSSELTON M0812_05704:g5632.t1.CDS
BUSSELTON M0812_00440:g442.t1.CDS
SCHOONER M0813_21898:g25967.t1.CDS
SCHOONER M0813_21860:g25929.t1.CDS
BUSSELTON M0812_14521:g14305.t1.CDS
Rab24
NgrRab24 Naegleria gruberi Rab20
IgaRab24 Isochrysis galbana Rab20
EhuRab24 Emiliania huxleyi Rab20
HsaRab20 Homo sapiens Rab20
XP_788580.1 Strongylocentrotus purpuratus Rab20
LgiRab20 Lottia gigantea Rab20
NveRab20 Nematostella vectensis Rab20
BMAN M0811_06877:cds.g6967.t1
BMAN M0811_00975:cds.g985.t1
BMAN M0811_00923:cds.g932.t1
BMAN M0811_10670:cds.g10802.t1
BMAN M0811_09402:cds.g9524.t1
BMAN M0811_06594:cds.g6681.t1
SCHOONER M0813_26176:g30186.t1.CDS
BUSSELTON M0812_18021:g17718.t1.CDS
SCHOONER M0813_30185:g34141.t1.CDS
BUSSELTON M0812_14678:g16037.t1.CDS
SCHOONER M0813_19523:g23622.t1.CDS
BUSSELTON M0812_01142:g1134.t1.CDS
SCHOONER M0813_27814:g31797.t1.CDS
BUSSELTON M0812_01335:g1324.t1.CDS
SCHOONER M0813_02887:g2834.t1.CDS
BUSSELTON M0812_11343:g11194.t1.CDS
SCHOONER M0813_01002:g977.t1.CDS
BUSSELTON M0812_18639:g18321.t1.CDS
Rab20

B)

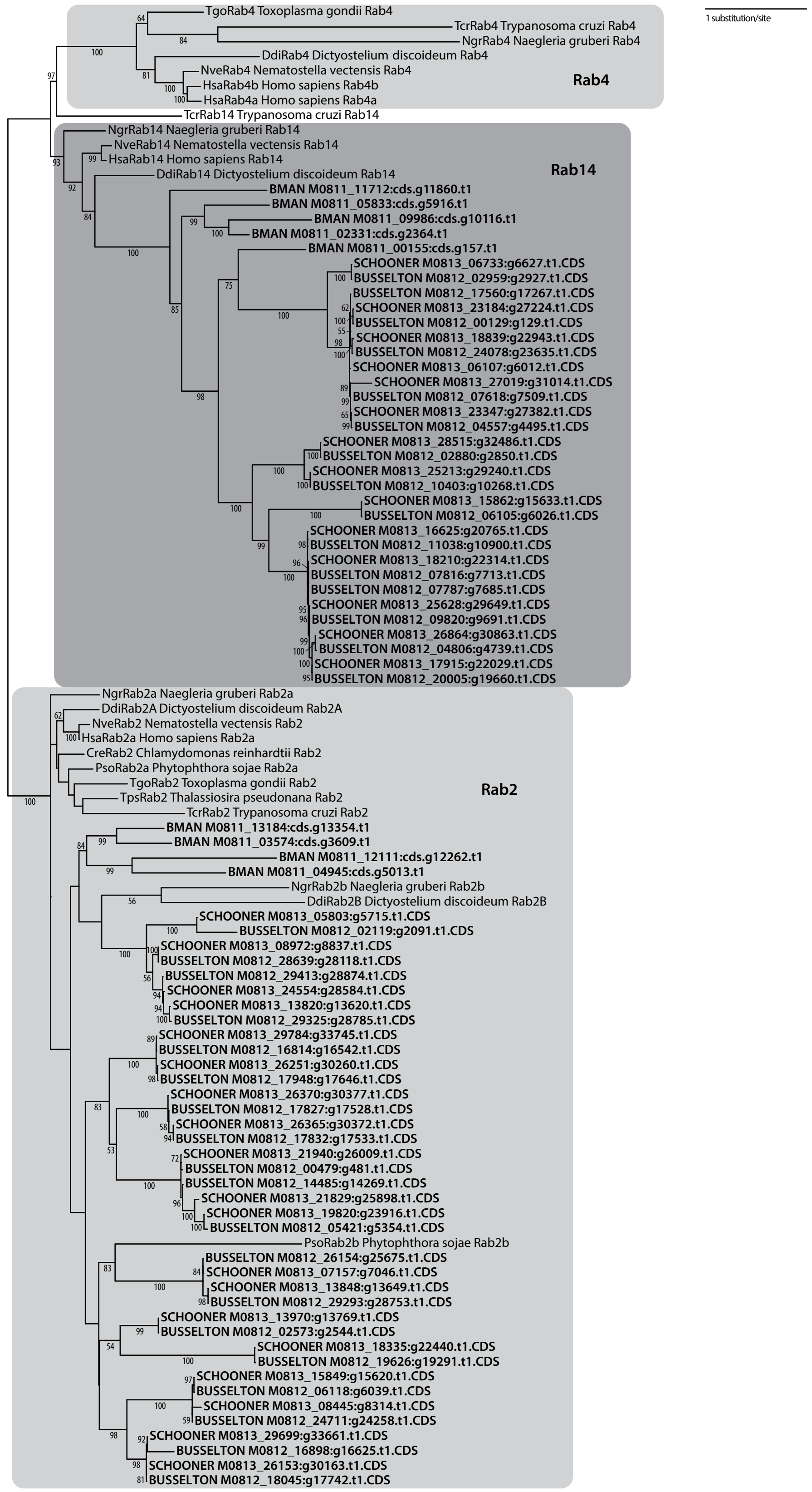
1 substitution/site
Rab4
TgoRab4 Toxoplasma gondii Rab4
TcrRab4 Trypanosoma cruzi Rab4
NgrRab4 Naegleria gruberi Rab4
DdiRab4 Dictyostelium discoideum Rab4
NveRab4 Nematostella vectensis Rab4
HsaRab4b Homo sapiens Rab4b
HsaRab4a Homo sapiens Rab4a
TcrRab14 Trypanosoma cruzi Rab14
Rab14
NgrRab14 Naegleria gruberi Rab14
NveRab14 Nematostella vectensis Rab14
HsaRab14 Homo sapiens Rab14
DdiRab14 Dictyostelium discoideum Rab14
BMAN M0811_11712:cds.g11860.t1
BMAN M0811_05833:cds.g5916.t1
BMAN M0811_09986:cds.g10116.t1
BMAN M0811_02331:cds.g2364.t1
BMAN M0811_00155:cds.g157.t1
SCHOONER M0813_06733:g6627.t1.CDS
BUSSELTON M0812_02959:g2927.t1.CDS
BUSSELTON M0812_17560:g17267.t1.CDS
SCHOONER M0813_23184:g27224.t1.CDS
BUSSELTON M0812_00129:g129.t1.CDS
SCHOONER M0813_18839:g22943.t1.CDS
BUSSELTON M0812_24078:g23635.t1.CDS
SCHOONER M0813_06107:g6012.t1.CDS
SCHOONER M0813_27019:g31014.t1.CDS
BUSSELTON M0812_07618:g7509.t1.CDS
SCHOONER M0813_23347:g27382.t1.CDS
BUSSELTON M0812_04557:g4495.t1.CDS
SCHOONER M0813_28515:g32486.t1.CDS
BUSSELTON M0812_02880:g2850.t1.CDS
SCHOONER M0813_25213:g29240.t1.CDS
BUSSELTON M0812_10403:g10268.t1.CDS
SCHOONER M0813_15862:g15633.t1.CDS
BUSSELTON M0812_06105:g6026.t1.CDS
SCHOONER M0813_16625:g20765.t1.CDS
BUSSELTON M0812_11038:g10900.t1.CDS
SCHOONER M0813_18210:g22314.t1.CDS
BUSSELTON M0812_07816:g7713.t1.CDS
BUSSELTON M0812_07787:g7685.t1.CDS
SCHOONER M0813_25628:g29649.t1.CDS
BUSSELTON M0812_09820:g9691.t1.CDS
SCHOONER M0813_26864:g30863.t1.CDS
BUSSELTON M0812_04806:g4739.t1.CDS
SCHOONER M0813_17915:g22029.t1.CDS
BUSSELTON M0812_20005:g19660.t1.CDS
Rab2
NgrRab2a Naegleria gruberi Rab2a
DdiRab2A Dictyostelium discoideum Rab2A
NveRab2 Nematostella vectensis Rab2
HsaRab2a Homo sapiens Rab2a
CreRab2 Chlamydomonas reinhardtii Rab2
PsoRab2a Phytophthora sojae Rab2a
TgoRab2 Toxoplasma gondii Rab2
TpsRab2 Thalassiosira pseudonana Rab2
TcrRab2 Trypanosoma cruzi Rab2
BMAN M0811_13184:cds.g13354.t1
BMAN M0811_03574:cds.g3609.t1
BMAN M0811_12111:cds.g12262.t1
BMAN M0811_04945:cds.g5013.t1
NgrRab2b Naegleria gruberi Rab2b
DdiRab2B Dictyostelium discoideum Rab2B
SCHOONER M0813_05803:g5715.t1.CDS
BUSSELTON M0812_02119:g2091.t1.CDS
SCHOONER M0813_08972:g8837.t1.CDS
BUSSELTON M0812_28639:g28118.t1.CDS
BUSSELTON M0812_29413:g28874.t1.CDS
SCHOONER M0813_24554:g28584.t1.CDS
SCHOONER M0813_13820:g13620.t1.CDS
BUSSELTON M0812_29325:g28785.t1.CDS
SCHOONER M0813_29784:g33745.t1.CDS
BUSSELTON M0812_16814:g16542.t1.CDS
SCHOONER M0813_26251:g30260.t1.CDS
BUSSELTON M0812_17948:g17646.t1.CDS
SCHOONER M0813_26370:g30377.t1.CDS
BUSSELTON M0812_17827:g17528.t1.CDS
SCHOONER M0813_26365:g30372.t1.CDS
BUSSELTON M0812_17832:g17533.t1.CDS
SCHOONER M0813_21940:g26009.t1.CDS
BUSSELTON M0812_00479:g481.t1.CDS
BUSSELTON M0812_14485:g14269.t1.CDS
SCHOONER M0813_21829:g25898.t1.CDS
SCHOONER M0813_19820:g23916.t1.CDS
BUSSELTON M0812_05421:g5354.t1.CDS
PsoRab2b Phytophthora sojae Rab2b
BUSSELTON M0812_26154:g25675.t1.CDS
SCHOONER M0813_07157:g7046.t1.CDS
SCHOONER M0813_13848:g13649.t1.CDS
BUSSELTON M0812_29293:g28753.t1.CDS
SCHOONER M0813_13970:g13769.t1.CDS
BUSSELTON M0812_02573:g2544.t1.CDS
SCHOONER M0813_18335:g22440.t1.CDS
BUSSELTON M0812_19626:g19291.t1.CDS
SCHOONER M0813_15849:g15620.t1.CDS
BUSSELTON M0812_06118:g6039.t1.CDS
SCHOONER M0813_08445:g8314.t1.CDS
BUSSELTON M0812_24711:g24258.t1.CDS
SCHOONER M0813_29699:g33661.t1.CDS
BUSSELTON M0812_16898:g16625.t1.CDS
SCHOONER M0813_26153:g30163.t1.CDS
BUSSELTON M0812_18045:g17742.t1.CDS

C)

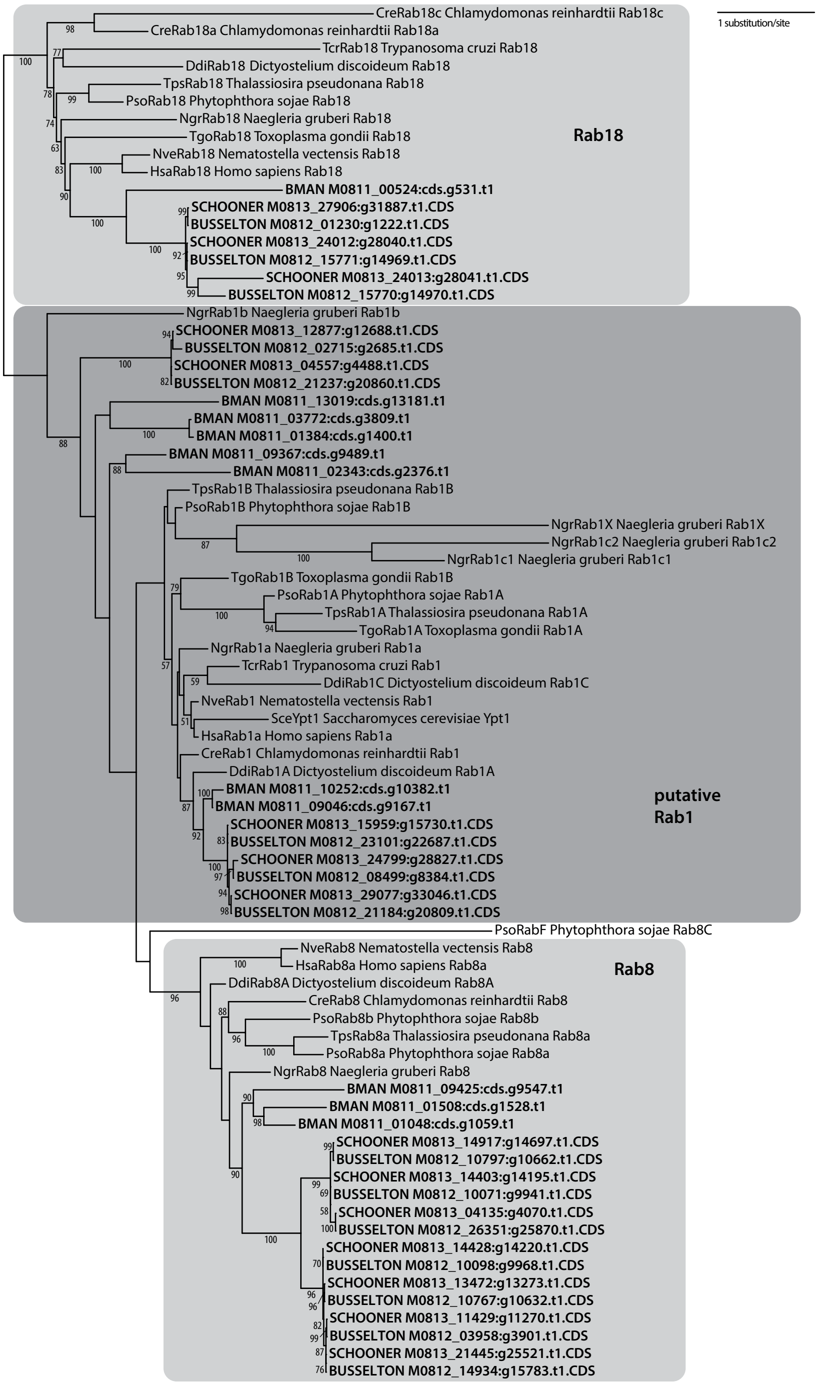

1 substitution/site
Rab18
CreRab18c Chlamydomonas reinhardtii Rab18c
CreRab18a Chlamydomonas reinhardtii Rab18a
TcrRab18 Trypanosoma cruzi Rab18
DdiRab18 Dictyostelium discoideum Rab18
TpsRab18 Thalassiosira pseudonana Rab18
PsoRab18 Phytophthora sojae Rab18
NgrRab18 Naegleria gruberi Rab18
TgoRab18 Toxoplasma gondii Rab18
NveRab18 Nematostella vectensis Rab18
HsaRab18 Homo sapiens Rab18
BMAN M0811_00524:cds.g531.t1
SCHOONER M0813_27906:g31887.t1.CDS
BUSSELTON M0812_01230:g1222.t1.CDS
SCHOONER M0813_24012:g28040.t1.CDS
BUSSELTON M0812_15771:g14969.t1.CDS
SCHOONER M0813_24013:g28041.t1.CDS
BUSSELTON M0812_15770:g14970.t1.CDS
NgrRab1b Naegleria gruberi Rab1b
SCHOONER M0813_12877:g12688.t1.CDS
BUSSELTON M0812_02715:g2685.t1.CDS
SCHOONER M0813_04557:g4488.t1.CDS
BUSSELTON M0812_21237:g20860.t1.CDS
BMAN M0811_13019:cds.g13181.t1
BMAN M0811_03772:cds.g3809.t1
BMAN M0811_01384:cds.g1400.t1
BMAN M0811_09367:cds.g9489.t1
BMAN M0811_02343:cds.g2376.t1
TpsRab1B Thalassiosira pseudonana Rab1B
PsoRab1B Phytophthora sojae Rab1B
NgrRab1X Naegleria gruberi Rab1X
NgrRab1c2 Naegleria gruberi Rab1c2
NgrRab1c1 Naegleria gruberi Rab1c1
TgoRab1B Toxoplasma gondii Rab1B
PsoRab1A Phytophthora sojae Rab1A
TpsRab1A Thalassiosira pseudonana Rab1A
TgoRab1A Toxoplasma gondii Rab1A
NgrRab1a Naegleria gruberi Rab1a
TcrRab1 Trypanosoma cruzi Rab1
DdiRab1C Dictyostelium discoideum Rab1C
NveRab1 Nematostella vectensis Rab1
SceYpt1 Saccharomyces cerevisiae Ypt1
HsaRab1a Homo sapiens Rab1a
CreRab1 Chlamydomonas reinhardtii Rab1
DdiRab1A Dictyostelium discoideum Rab1A
BMAN M0811_10252:cds.g10382.t1
BMAN M0811_09046:cds.g9167.t1
SCHOONER M0813_15959:g15730.t1.CDS
BUSSELTON M0812_23101:g22687.t1.CDS
SCHOONER M0813_24799:g28827.t1.CDS
BUSSELTON M0812_08499:g8384.t1.CDS
SCHOONER M0813_29077:g33046.t1.CDS
BUSSELTON M0812_21184:g20809.t1.CDS
putative
Rab1
PsoRabF Phytophthora sojae Rab8C
Rab8
NveRab8 Nematostella vectensis Rab8
HsaRab8a Homo sapiens Rab8a
DdiRab8A Dictyostelium discoideum Rab8A
CreRab8 Chlamydomonas reinhardtii Rab8
PsoRab8b Phytophthora sojae Rab8b
TpsRab8a Thalassiosira pseudonana Rab8a
PsoRab8a Phytophthora sojae Rab8a
NgrRab8 Naegleria gruberi Rab8
BMAN M0811_09425:cds.g9547.t1
BMAN M0811_01508:cds.g1528.t1
BMAN M0811_01048:cds.g1059.t1
SCHOONER M0813_14917:g14697.t1.CDS
BUSSELTON M0812_10797:g10662.t1.CDS
SCHOONER M0813_14403:g14195.t1.CDS
BUSSELTON M0812_10071:g9941.t1.CDS
SCHOONER M0813_04135:g4070.t1.CDS
BUSSELTON M0812_26351:g25870.t1.CDS
SCHOONER M0813_14428:g14220.t1.CDS
BUSSELTON M0812_10098:g9968.t1.CDS
SCHOONER M0813_13472:g13273.t1.CDS
BUSSELTON M0812_10767:g10632.t1.CDS
SCHOONER M0813_11429:g11270.t1.CDS
BUSSELTON M0812_03958:g3901.t1.CDS
SCHOONER M0813_21445:g25521.t1.CDS
BUSSELTON M0812_14934:g15783.t1.CDS

D)

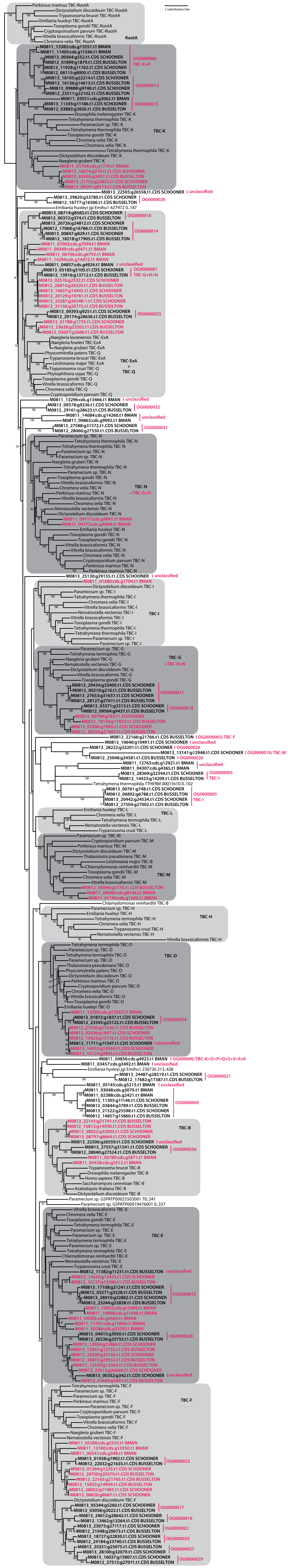
1 substitution/site
RootA
TBC-K
TBC-ExA
+
TBC-Q
TBC-N
+TBC-G+H
TBC-I
TBC-G
+TBC-H+N
TBC-L
TBC-M
TBC-H
TBC-D
TBC-B
TBC-E
TBC-F

Figure S19: Phylogenetic analyses of Rab and TBC proteins in *Anaeramoeba*. a-c, Phylogenetic analyses of Rab proteins of subfamilies containing endocytic Rabs **a,** Rabs 2, 4, and 14 **b,** and Rabs 1, 8, and 18 **c,**. **d,** Phylogenetic analysis of TBC proteins. *Anaeramoeba* sequences are in bold. Sequences having the same identity in the phylogenetic analysis and orthogroup (OG) clustering (Supplementary Data 6D) are in pink. Sequences in black have OG identity displayed next to them. Tree files and alignments are available at FigShare: https://doi.org/10.6084/m9.figshare.22193497.v1

a.

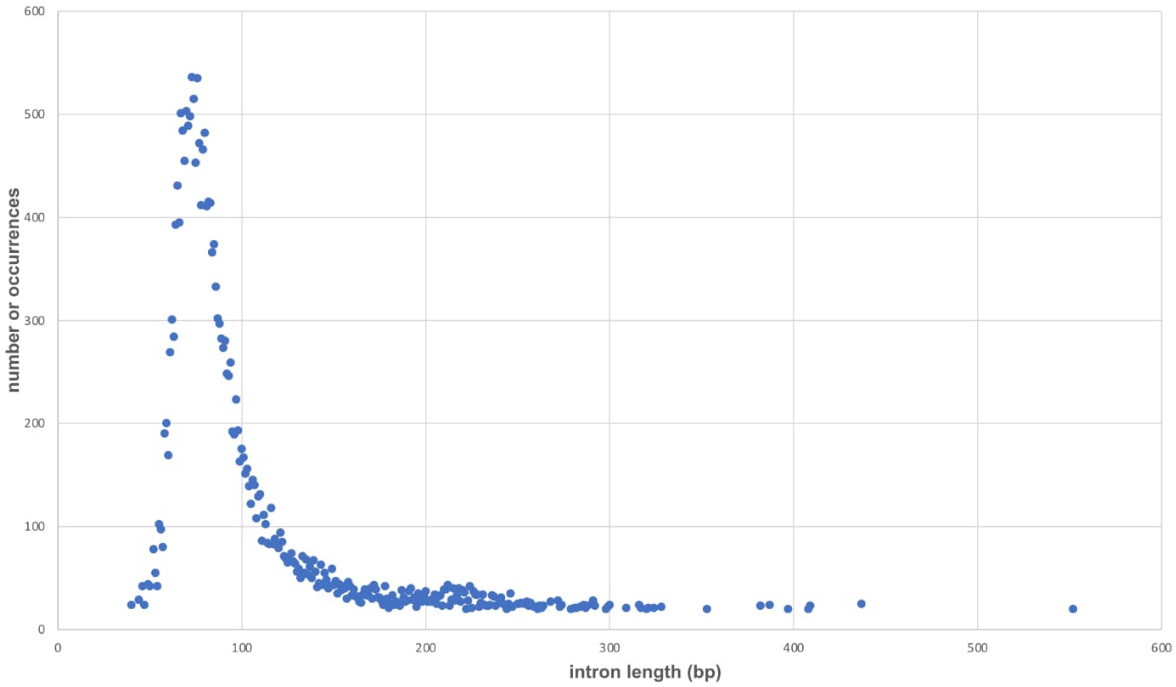

Figure S20A Intron lengths in *A. flamelloides* BMAN.

b.

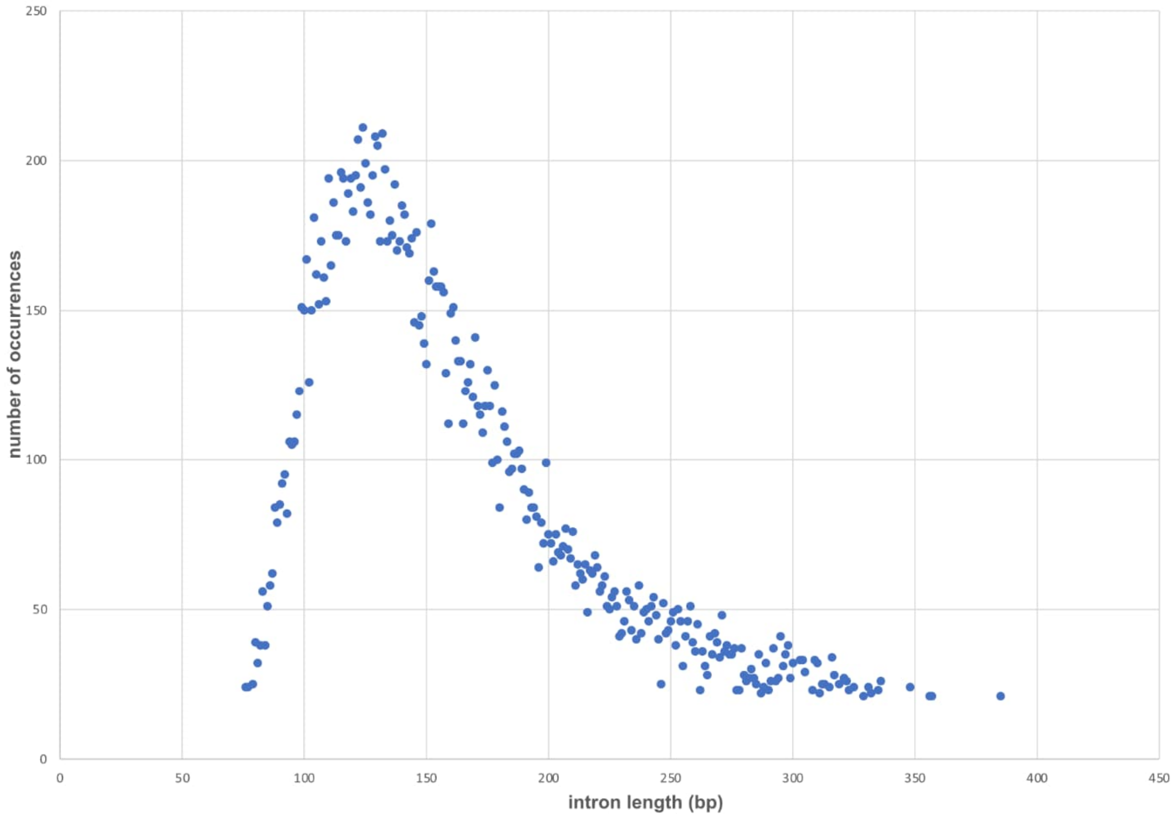

Figure S20B Intron lengths in *A. flamelloides* BUSSELTON2.

c.

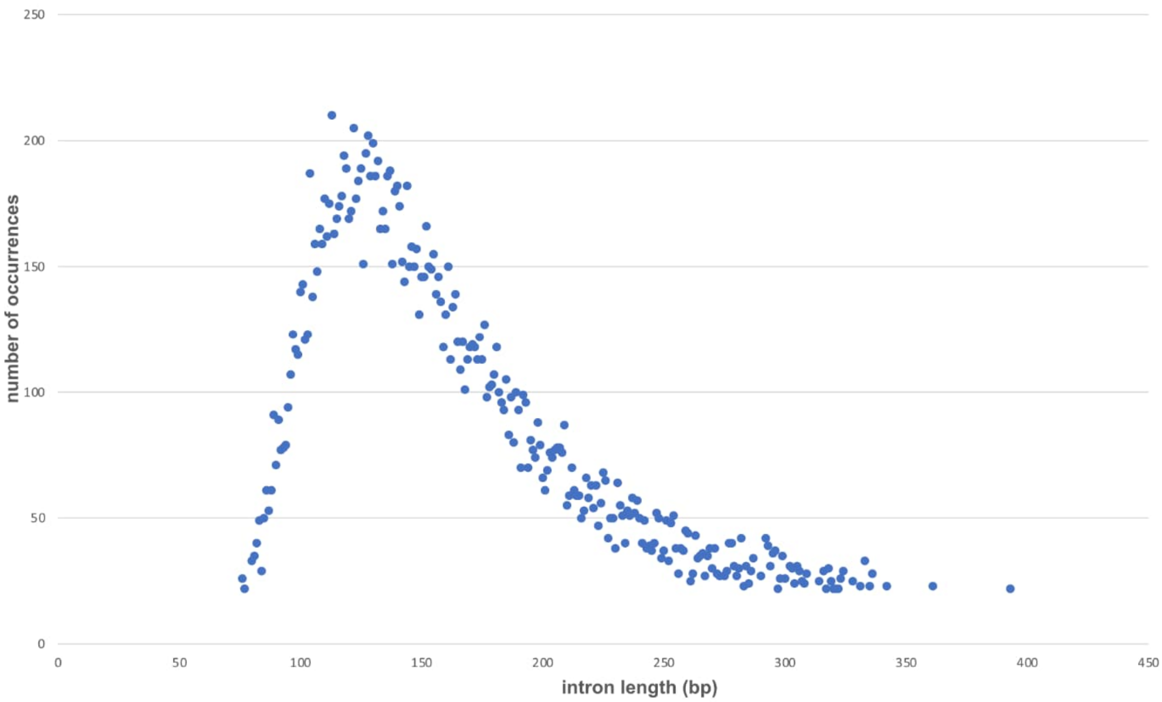

Figure S20C Intron lengths in *A. flamelloides* SCHOONER1.

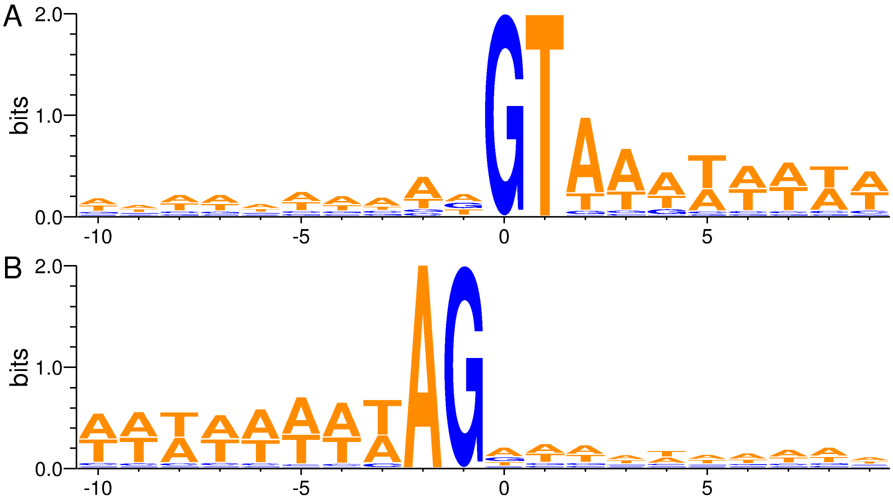

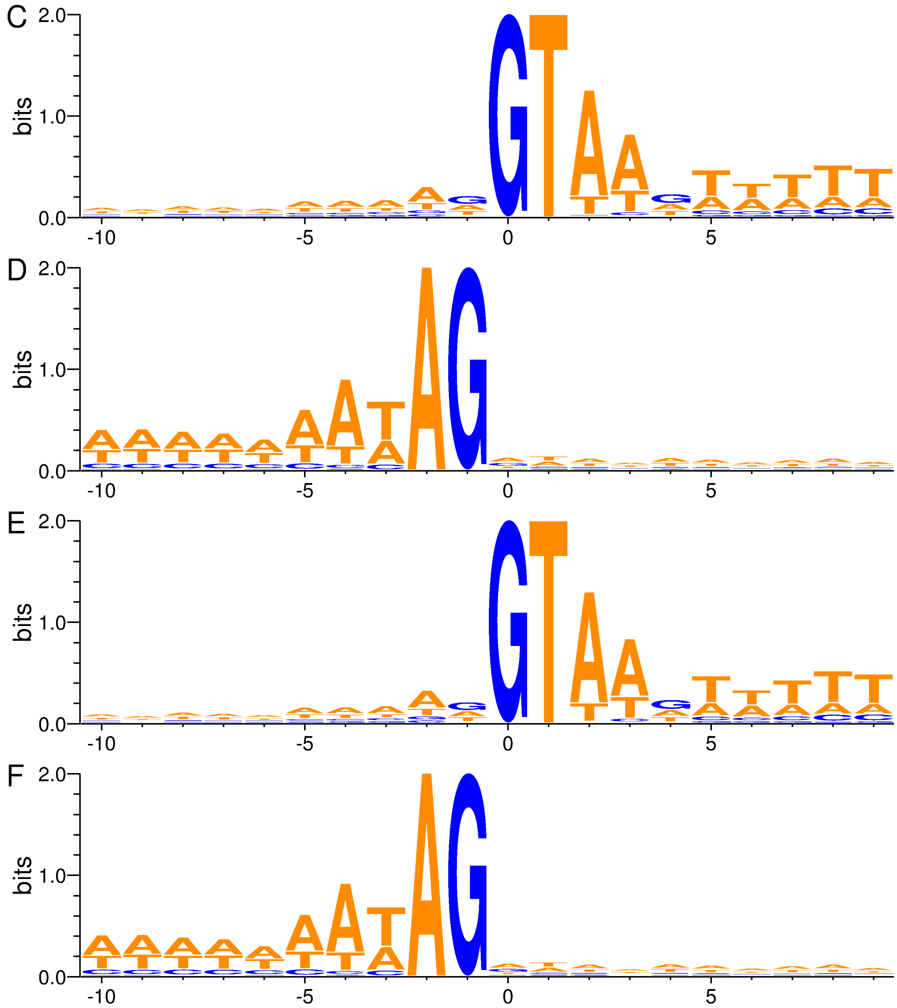

Figure S20: Introns characteristics in *Anaeramoeba* a, Intron lengths in *A. ignava* BMAN, **b,** *A. flamelloides* BUSSELTON2, **c,** *A. flamelloides* SCHOONER1. **d,** Characteristics of intron splice sites. Sequence logo plots showing subpanel A., 10 bases upstream of donor sites and 8 downstream in BMAN, subpanel B., 8 bases upstream of acceptor site and 10 bases downstream in BMAN, subpanel C., 10 bases upstream of donor sites and 8 downstream in BUSSELTON2, subpanel D., 8 bases upstream of acceptor site and 10 bases downstream in BUSSELTON2, subpanel E., 10 bases upstream of donor sites and 8 downstream in SCHOONER1, subpanel F., 8 bases upstream of acceptor site and 10 bases downstream in SCHOONER1. The sequence logos were generated using the WebLogo3 server [5].

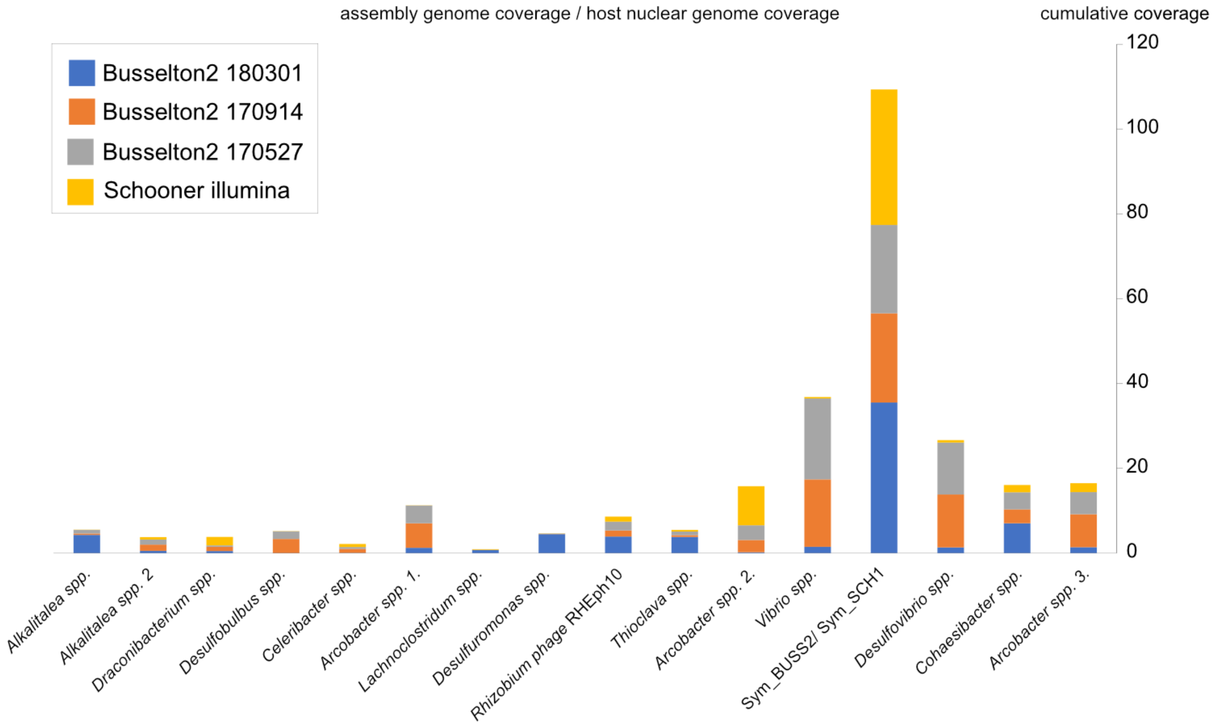

Figure S21: Sym_BUSS2 / Sym_SCH1 are consistently associated with *Anaeramoeba flamelloides*. Four sequencing datasets generated from DNA from four individual *A. flamelloides* cell isolations were mapped using minimap2 against either *A. flamelloides* BUSSELTON2 or SCHOONER1 genome and the total metagenome assemblies. The assembly genome / host nuclear genome coverages were calculated from bam-files using coverage statistics from pileup.sh in the BBMap package v38.20 (sourceforge.net/projects/bbmap/). The cumulative coverage was plotted with coverage from the different replicates indicated by blue (*A. flamelloides* BUSSELTON2 180301), orange (*A. flamelloides* BUSSELTON2 170914), grey (*A. flamelloides* BUSSELTON2 170527) and yellow (Schooner illumina). The different labels on the x-axis are major assembly components derived from metagenomic assemblies of *A. flamelloides* BUSSELTON2 and SCHOONER1. The assembly component annotations were assigned using the top blastn hit in NCBI.

Supplementary Tables

Table S1 General genome statistics for *A. ignava BMAN, A. flamelloides* BUSSELTON2 *and A. flamelloides* SCHOONER1*.*

	Anaeramoeba ignava BMAN	*Anaeramoeba flamelloides* BUSSELTON2	*Anaeramoeba flamelloides* SCHOONER1
Genome size (Mbp)	42,843,398	254,083,75	259,028,67
# of scaffolds	147	76	346
N50	512,274	12,797,701	1,575,887
GC%	19.35	23.63	24.77
# of genes	14,837	30,401	30,317
# of CDSs	14,759	29,744	29,839
# of tRNAs	74	637	456

Table S2 Symbiont genome characteristics.

	Sym_BMAN	Sym_BUSS2	Sym_SCH1	*Desulfobacterium autotrophicum*
Chromosome size (bp)	6,055,721 (closed circular)	4,968,664 (closed circular)	4,994,604 (26 contigs)	5,657,780
CDS (functional)	5,256	3,840	3,823	4,768
GC%	52.96	47.60	47.50	48.80
Pseudogenes	37	922	1,000	79
IS elements[1]	60	624	666	75
Symbionts/amoeba	12.6	35.3	36.5	-

[1]Predicted by ISsaga v2.0 (http://issaga.biotoul.fr/ISsaga2/).

Table S3A Introns

	Anaeramoeba ignava BMAN	*Anaeramoeba flamelloides* BUSSELTON2	*Anaeramoeba flamelloides* SCHOONER1
# of introns	32,108	39,831	39,247
Average length (bps)	300	1,459	1,504
Median length (bps)	102	264	278
Mode length (bps)	76	124	113
Longest (bps)	4,168	10,402	10,385
Shortest (bps)	35	49	44
Percentage of genome	22.5	22.8	22.7
Introns/genomic kb	0.74	0.15	0.15
Introns/gene	2.13	1.3	1.3
GC% of all introns (genome)	8.14 (19.36)	22.8 (23.63)	22.06 (23.78)

Table S3B Noncanonical intron boundaries

	Anaeramoeba ignava BMAN	*Anaeramoeba flamelloides* BUSSELTON2	*Anaeramoeba flamelloides* SCHOONER1
AT-AC	33	0	0
GC-AG	20	7	6
GT-AA	1	0	0
TA-AG	1	0	0
5’ (G/A)TATCCTTT	32 (27 A, 5 G)	8 (8 G)	10 (10 G)

Supplementary Note 1

Anaeramoeba host genomes:

Introns in Anaeramoeba

Among the three genomes analyzed, SCHOONER1 and BUSSELTON2 host genomes have a very similar intron complement in terms of the number of introns overall (Table S3A) and their size ranges (Table S3, Figure S20). The genomic context is equally similar with regard to the percentage of the genome devoted to introns, the GC% of all the introns and the number of introns per gene and per genomic kb. The BMAN genome has almost as many introns (Table S3A) as SCHOONER1 and BUSSELTON2 (32,108, 39,247, 39,831 respectively) but apart from this the characteristics diverge. BMAN introns tend to be much smaller with a mode length of 76 versus 113 for SCHOONER1 and 124 for BUSSELTON2 and the longest intron found in BMAN (4168 bps) is less than half of the longest ones in the other genomes (Table S3A, Figure S20). Equally striking is the GC% of the introns. While SCHOONER1, BUSSELTON2 and BMAN all have very GC% poor genomes (23.78, 23.63 and 19.36 respectively), the introns in BMAN are even more GC% poor averaging only 8.14 GC% which is less than half of the genome globally while the GC percentage of introns in SCHOONER1 and BUSSELTON2 are close to their overall genomes (Table S3A). The AT richness of all three genomes is exemplified in the bases immediately before and after the splice sites. The surrounding bases are heavily weighted towards As and Ts (Figure S20D). Because the BMAN genome is 1/6th the size of the other two genomes while having almost as many introns, its number of introns per kb is considerably higher (0.74 versus 0.15). The smaller genome size of BMAN, while having almost as many introns, also accounts for why the percentage of intronic sequence in all three genomes is very similar.

The number of introns that possess non-standard splice sites (5' GT, 3' AG) in the three genomes is extremely small but also in line with other sequenced genomes [6–8]. Only 55 of 32108 (0.17%) introns in BMAN had noncanonical boundaries while in SCHOONER1 and BUSSELTON2 the numbers were even lower, 6 and 7 respectively (Table S3B). All of the noncanonical intron boundaries in SCHOONER1 and BUSSELTON2 were of the GC-AG type while in BMAN 33 introns had AT-AC splice sites. Traditionally, AT-AC boundaries were considered diagnostic for U12 type introns. However, this is no longer the case and whether an intron is spliced by the major or minor (U12 type) spliceosome is determined by analyzing the branch point and critically, the first few bases in the intron [9]. U12 type introns have a highly conserved motif at the 5' end, starting with the splice site. The first base can be either an A or a G followed by TATCCTTT. BMAN has 32 introns that start with (G/A)TATCCTTT (Table S3B) with 27 of them being AT-AC introns while in SCHOONER1 all 10 of the introns that start with the conserved motif are GT-AG introns as are the 8 in BUSSELTON2 that have the motif. The presence of the distinctive U12 5' motif in this limited set of introns strongly suggests that this lineage has a minor spliceosome. However, there was no conserved branch point sequence among these potential U12 type introns. None of the branch points were similar to the reported conserved branch point motif [9]. However, it should be noted that the published branch point motif is based on a very limited set of introns from model organisms. The fact that *Anaeramoebae* shares the 5' motif with the model organisms but not the branch point motif suggests that only the 5' motif is truly diagnostic for U12 type introns.

Gene expansion and contraction analysis

We compared the gene families of the three *Anaeramoeba* genomes to nine other microbial eukaryotes including those from Parabasalia (*Trichomonas vaginalis*), Oxymonadida

(*Monocercomonoides exilis*), Fornicata (*Carpediemonas frisia*, *Carpediemonas membranifera*, *Kipferlia bialata*, *Giardia intestinalis*, *Spironucleus salmonicida*), Heterolobosea (*Naegleria gruberi*), and Amorphea (*Dictyostelium discoideum*). Across all genomes, a total of 565 and 4,704 families were deemed core and accessory, respectively (Supplementary Data 4A-C). 1,806 gene families are taxon-specific, and of these, 67, 74 and 107 are specific to BUSSELTON2, SCHOONER1 and BMAN, respectively (Supplementary Data 4D). When comparing *Anaeramoeba* to other metamonads (parabasalids, oxymonads and fornicates), *Naegleria gruberi* and *Dictyostelium discoideum*, 92 families were considered specific to *Anaeramoeba* (Supplementary Data 4E). Note that only 557 core and 1,557 accessory families were retained for the protein family expansion/contraction analysis across all taxa after filtering. Expansions $\geq$ 5-fold were observed in 110 core and 448 accessory families, whereas contractions were only detected in 18 accessory families (Supplementary Data 4F,G). When comparing only *A. flamelloides* to *A. ignava*, we identified 2,597 core and 688 accessory families but only 2,578 and 89 of those were retained for the analysis (Supplementary Data 4F). Of these, expansions were detected in 330 core families while contractions were found in 13 core families (Supplementary Data 4H). The contractions of *A. flamelloides* likely correspond to expansions in *A. ignava* rather than contractions in *A. flamelloides* (Supplementary Data 4F,H).

Enrichment of ribosomal proteins suggest specialized compartmentalization in A. flamelloides

Ribosomes are formed by rRNA and ribosomal proteins (RPs). In eukaryotes, they consist of a small subunit (40S) formed by 18S rRNA and up to 33 RPs, and of a large (60S) subunit formed by 28S, 5.8S, and 5S rRNAs and up to 46 RPs [10]. We retrieved two 18S rRNA sequences for BUSSELTON2 located in different scaffolds, one is a fragment of 1041bp and the other one is 3654bp long sharing 99% identity, and also two sequences for SCHOONER1 of 3362 bp and 3446 bp long with shared identity of 99.3%. Five and seven 28S rRNA sequences with varied lengths (i.e., 1384 to 4562 bp, with identities ranging from 90-99.8%) were identified in different SCHOONER1 and BUSSELTON2 scaffolds, respectively. We were able to validate most of the annotated RPs from 40S and 60S subunits that were flagged by our expansion analysis (Figure S16, Supplementary Data 4J,K). In addition, our findings showcase *Anaeramoeba*'s RPs repertoire as the most complete among all metamonads studied, and that expansions in *A. flamelloides* correspond to a recent event that occurred in the last common ancestor of SCHOONER1 and BUSSELTON2. Proteins within each family are very similar in size and percentage identity at the amino acid and nucleotide levels. Given these high identities, we queried 10 proteins located up and downstream of each RP ortholog to find out whether they were similar or shared similar functional annotations with other scaffolds along loci containing their respective RP ortholog. We found that RPs-flanking proteins belong to loci associated with varied functions that were rarely shared among the RPs-flanking regions of their respective orthologs in all comparisons. This reinforces that those are bona fide expansions and not assembly duplication artifacts. Since RPs were expanded, we looked for evidence of expansion in tRNAs with tRNA-scan-SE [11]. This yielded 667, 682 and 111 tRNA copies in BUSSELTON2, SCHOONER1, and BMAN, respectively. The copy numbers for BUSSELTON2 and SCHOONER1 appear to be consistent with the expansion of gene families associated to translation metabolic pathways.

The near complete 40S and 60S RPs repertoire, their large abundances, and the expansion in tRNA genes may suggest that *A. flamelloides* strains are able to assemble 'subpopulations' of ribosomes, and given the intricate relationships that have probably been developed between the host and symbionts, it may be likely that these subpopulations of ribosomes can have unique properties that influence the functions of the proteins they produce, in a similar way as shown by new evidence from model organisms (reviewed in [12]). Hence, since ribosomal proteins provide access to unique modes of translation, it might be possible that *A. flamelloides*

has developed some sort of ‘ribosome code’ as ribosomes assembled with varied ribosomal protein composition may confer specialized functions, and ribosomal proteins paralog specificity may define novel means of translational control [12–14].

Supplementary Note 2

Lateral gene transfers into the Anaeramoebae:

Extended analyses of selected LGT candidates

Stairs et al. used transcriptomics to reconstruct the metabolism of the MRO of *A. ignava* and *A. flamelloides* BUSSELTON2 and found it to be a hydrogenosome. Several hydrogenosome proteins were detected in our LGT screen and are involved in several aspects of hydrogenosome metabolism such as dealing with oxidative stress and amino acid synthesis (see Supplementary Data 5). The donor taxa of the hydrogenosomal LGT-derived genes are variable. These authors also found that *Anaeramoeba* spp. have several enzymes involved in ATP production that were not found in other Metamonada [15]. Some of these enzymes were acquired through LGT. For example, a bacterial 3-hydroxybutyrate dehydrogenase is found in *A. ignava* (Figure S17A), and two bacterial methylmalonyl CoA epimerases (MME) of independent origin are found in *A. flamelloides* (Figure S17B). Interestingly, *A. flamelloides* also has a eukaryotic version of the enzyme which is the one predicted to be targeted to the MRO (*A. ignava* only has a eukaryotic MME).

We used KofamKoala [16] to assess the extent to which *Anaeramoeba* contains metabolic "modules" (sub-pathways) encoded entirely by LGT-derived genes. We found that strongly supported LGT-derived proteins typically form chimeric pathways with eukaryotic proteins (note that less than a third of the LGT-derived genes were successfully annotated by the software). The sole exception is an apparently intact Leloir pathway in *A. flamelloides*, which suggests that the organism can metabolize galactose. Interestingly, while the pathway is typically comprised of four stand-alone enzymes, the *A. flamelloides* Leloir pathway contains two fused proteins. BUSSELTON_g20257.t1 and SCHOONER_g30019.t1 are fusions of galactose mutarotase (GalM) and galactokinase (acetyl-CoA carboxylase, GalK), and BUSSELTON_g13285.t1 and SCHOONER_g28623.t1 are fused galactokinase (GalK), galactose 1-P uridyltransferase (GalT) and UDP-galactose 4 epimerase (GalE). Phylogenetic reconstructions of each of the four enzymes shows that members of the bacterial phylum "Candidatus Omnitrophica" branch as sister taxa and thus could be the donor lineage (Figure S17C). The *Anaeramoeba* Leloir pathway genes could be derived from an operon, which would have facilitated fusion protein formation after LGT. Due to the fragmented nature of available genomic assemblies for "Candidatus Omnitrophica", we were not able to determine whether the Leloir pathway genes indeed reside in an operon in this organism. However, such genes are operonic in other bacteria such as *E. coli* [17] (i.e., GalETKM). Interestingly, whereas the utilization of galactose has apparently been lost in the *A. flamelloides* symbionts due to the loss of *galE*, a complete Leloir pathway is present in Sym_BMAN.

We observed differences in the predicted high-level functions of LGTs inferred to have been acquired in the common ancestor of *A. ignava* and *A. flamelloides* and those that were acquired after the two species diverged (the possibility of secondary loss must also be acknowledged). Of the genes acquired in the *Anaeramoeba* common ancestor, functional categories E (amino acid metabolism), C (Energy metabolism) and G (Carbohydrate metabolism) are the most highly represented (Figure 5C, blue bar). This is similar to what was observed in *Mastigamoeba,* a LGT-prone eukaryote found in anaerobic environments [18], as well as in diverse parasitic eukaryotes [19].

We analyzed proteins predicted to have been acquired by LGT in the common ancestor of *A. ignava* and *A. flamelloides* in order to assess how they might 'plug in' to the metabolism of the *Desulfobacteraceae* symbionts. The results speak strongly to the complementarity of the partner organisms. For example, a cysteine synthase K and a D-3-phosphoglycerate dehydrogenase are LGT-derived (Figure S17D,E), and together with SerC phosphoserine aminotransferase (which is itself not detected in our LGT screen but found in the genomes), these enzymes are linked to glycolysis, to the re-use of SH^- produced by the symbionts, and to provision of acetate to the symbionts (Figure S18). This is one example where an *Anaeramoeba* pathway is composed of eukaryotic, putatively ancestral enzymes, and LGT-derived enzymes.

Another example of metabolic complementarity involves an acetate transporter (Figure S17F) acquired in the *Anaeramoeba* common ancestor. Acquisition of this enzyme by LGT might have played a role in the establishment of the symbiosis. From the data in hand it is not clear whether this LGT directly facilitates the transport of acetate from *Anaeramoeba* to the symbiont or if it simply imports acetate from the environment that could be routed to the symbiont secondarily. In any case, it is noteworthy that the acetate transporter gene has duplicated numerous times after acquisition, which is consistent with functional specialization (*A. ignava* has seven paralogs, *A. flamelloides* BUSSELTON2 has 35, and *A. flamelloides* SCHOONER1 has 32).

Genes associated with anaerobiosis were also acquired in the common ancestor of *A. ignava* and *A. flamelloides*. For example, and as has been described in several diplomonads [20] and other anaerobic protists [21], *Anaeramoeba* spp. seem able to perform pyrophosphate-dependent glycolysis, which uses pyrophosphate (PPi) instead of ATP as a phosphate donor. This capacity appears to have been enabled by the *Anaeramoeba* ancestor having two pyruvate phosphate dikinase (PPD) enzymes (Figure S17G), one shared with Parabasalia and thus likely acquired before *Anaeramoeba* and *Trichomonas* diverged from one another. The other PPD is exclusive to *Anaeramoeba*. Two other enzymes acting downstream of PPD are also found in *Anaeramoeba* spp. but were not detected in our LGT screen, due to presence of eukaryote-eukaryote LGTs after an initial acquisition from bacteria. These two enzymes are ATP-independent phosphofructokinase (Figure S17H) and phosphoglycerate mutase (Figure S17I). *A. ignava* has also acquired a second copy of phosphoglycerate mutase (Figure S17J). The chimeric nature of glycolysis in *Anaeramoeba* (involving both prokaryotic and eukaryotic enzymes) may have enhanced ATP production, which would have been crucial given their lack of a TCA cycle, which relies on oxidative phosphorylation [22].

Genes allowing survival in anoxic environments were also acquired multiple times in the history of *Anaeramoeba*. Strategies for mitigating the loss of the eukaryotic aerobic ribonucleotide reductase (RNR) in other metamonads include reliance on the salvaging of exogenous deoxynucleosides in *Giardia* [23], acquisition of class III RNR from prokaryotes in *Spironucleus*, *Trepomonas* [24] and *Kipferlia bialata* [20,25], and acquisition of class II RNR (*Trichomonas* and *Tritrichomonas*) [26], as well as a bacterial class I RNR in *Tritrichomonas*. *A. ignava* and *A. flamelloides* have different RNR gene suites, none of which encode the classical oxygen sensitive eukaryotic class I RNR but instead encode several class II RNRs, which are vitamin B12 dependent and oxygen insensitive (Figure S17K). *A. flamelloides* RNRs branch with the few known eukaryotic RNR class II enzymes (monomers) that are thought to have been acquired from bacteria or viruses on several occasions (Figure S17K) [26,27], and were also putatively transferred among eukaryotes [26]. By contrast, BMAN RNRs constitute (at least) two evolutionarily distinct branches. One shares recent common ancestry with *Trichomonas vaginalis* RNR class II and archaeal RNR class II (dimer), as well as bacterial enzymes (Figure S17K), and another branches ambiguously with diverse bacteria.

The *Anaeramoeba* common ancestor appears to have acquired enzymes to deal with oxidative stress via LGT. For example, as mentioned in [15], several NADH oxidases are found in *Anaeramoeba.* Interestingly, they were acquired on multiple occasions in *Anaeramoeba* history from different donors (see next section for a global screening of serial acquisitions) (Figure S17L), likely including *Desulfobacteraceae* (Figure S17L). Other anaerobic protists have NADH oxidases but they do not belong to the same clade, suggesting that they were acquired independently [28,29].

Serially acquired genes in Anaeramoeba *speak to continual adaptive pressures*

We found evidence of serially acquired genes in *Anaeramoeba*. A total of 45 orthologs are predicted to have been acquired multiple times in one or both *Anaeramoeba* species. Additionally, 25 orthologs were independently acquired in *A. ignava* and *A. flamelloides* (or acquired in their common ancestor and then differentially lost; see Supplementary Data 5). These results are summarized below.

Orthologous genes involved in fighting oxidative stress were acquired multiple times in *Anaeramoeba* history. The phylogeny of OsmC proteins (Figure S17M) suggests that they were acquired (at least) six times, four times in *A. flamelloides* and twice in *A. ignava*, from various donors. Nitroreductases were acquired once in their common ancestor, and then twice in *A. flamelloides* and once in *A. ignava* (Figure S17N). Note that the phylogeny of nitroreductases suggests that several eukaryotes, many of which are anaerobes (*Trichomonas foetus* and *vaginalis*, several species of the *Entamoeba* genus, *Stygiella incarcerata*, and *Blastocystis* subtype 1) have also acquired nitroreductases from prokaryotes—and separately from *Anaeramoeba*. Thioredoxins were also acquired independently in both *Anaeramoeba* species while a rubrerythrin gene was acquired in the *Anaeramoeba* common ancestor and once in *A. flamelloides* (Figure S17O). This suggests that LGT is an ongoing feature of *Anaeramoeba*, one that gives rise to functionally differentiated genes that impact the biology of the organism. A more complete set of LGT-derived genes involved in oxygen detoxification is presented in Supplementary Data 5. Several of these genes were previously described as having been laterally acquired in several Metamonada [20]. Strikingly, the evolutionary origins of these genes are diverse: bacteria, archaea and viruses are all predicted donors.

Biosynthesis of sterols, which are key constituents of canonical eukaryotic membranes, requires molecular oxygen. In contrast, prokaryotic hopanoid biosynthesis does not require molecular oxygen as a substrate, and the squalene is directly cyclized by the enzyme squalene-hopene cyclase (SHC) [30]. Several anaerobic eukaryotes have been shown to encode a bacterial-like SHC, more specifically a squalene tetrahymenol cyclase (STC) that was likely transferred between eukaryotes after an initial acquisition from bacteria [31]. More recently, the anaerobic yeast *Schizosaccharomyces japonicus* was shown to have acquired a bacterial SHC independently, allowing growth in sterol-free media under anaerobic conditions [32]. Strikingly, *A. flamelloides* possess two evolutionarily distinct SHC genes. The first forms a sister branch with eukaryotic STCs, while the second one emerges among bacterial SHCs (Figure 5F). Additionally, the *A. ignava* and *A. flamelloides* ancestor appears to have acquired one of the enzymes of the mevalonate pathway from archaea, i.e., hydroxymethylglutaryl-CoA reductase (one of the pathways synthesizing the terpenoid backbone). As all the other enzymes in the pathway are eukaryotic, this represents an example of orthologous replacement of the eukaryotic enzyme by its prokaryotic counterpart, resulting in a chimeric mevalonate pathway.

Two Nim genes encoding 5-nitroimidazole reductases or related pyridoxamine 5'-phosphate oxidase were independently acquired by *A. ignava* and *A. flamelloides* from various donors (Figure S17P). Nim proteins are associated with acquired resistance to metronidazole in bacterial anaerobes [33]. Interestingly, other anaerobic protists have acquired Nim genes from bacteria [34], including *Trichomonas vaginalis*; in this case Nim proteins are targeted to the

hydrogenosome [35] (and in another recently sequenced parabasalid, *Histomonas meleagridis* KAH0787444.1). Their exact role in metroimidazole resistance is still under investigation [34] but their independent acquisitions in several anaerobic protists suggests that they are functionally important. Interestingly, while the *Desulfobacter* spp. associated with *A. ignava* has a full-length Nim gene, the Nim genes of the epibionts of *A. flamelloides* are pseudogenes.

Three genes involved in the synthesis of lysine, belonging to each of two known pathways were acquired either independently or multiple times in *Anaeramoeba* spp. (two genes in the diaminopimelic acid pathway and one in the homocitrate-aminoadipate pathway) (Figure S17Q,R). A branch comprising eukaryotic and viral ornithine decarboxylases is present in the diaminopimelate decarboxylase tree, suggesting that viruses could have mediated a eukaryote-to-eukaryote gene transfer. Both *Anaeramoeba* species also acquired several genes involved in leucine biosynthesis. They possess stand-alone LeuC and LeuD (large and small subunits of isopropyl malate isomerase, respectively), as well as a LeuC-LeuD fusion (Figure S17T,U). These genes were seemingly acquired separately, as the trees for each subunit show that the stand-alone and fused versions do not cluster together (Figure S17T). *A. ignava* fused LeuC-LeuD has eight introns while the standalone subunits have none, suggesting that the former was acquired earlier than the latter. In line with this hypothesis, the standalone subunits share 66.6 % identity with their best prokaryotic hit on average (Supplementary Data 5) (the fused versions still have on average 57.8 % of identity with their best prokaryotic hits). Interestingly, the fused and unfused versions of the LeuC and LeuD genes in *A. ignava* BMAN are next to each other in the genome despite their independent origin (in the following order: LeuD; LeuC; LeuD-LeuC fusion). This suggests that rearrangements happened after acquisition.

Finally, the phylogeny of two FAD-dependent dehydrogenases (Figure S17V) belonging to the same COG underscores the extent to which LGT has impacted *Anaeramoeba* genomes. One LGT-derived gene is present in both species and emerges among archaeal sequences. *A. flamelloides* encodes a second FAD-dependent dehydrogenase. Its closest sister branch is an enzyme from a eukaryotic dsDNA virus and bacterial sequences. The topology of the latter clade is also compatible with a virus being an intermediate between prokaryotic species and *Anaeramoeba*.

Supplementary Note 3

Vitamin B12 in Anaeramoebae and their symbionts

Extended analyses of vitamin B12 in Anaeramoeba *and* Desulfobacter *symbionts*

Vitamin B12 (vitB12), also known as cobalamin, is a complex Co^{2+}-containing modified tetrapyrrole that acts as a cofactor. In prokaryotes, there are over 15 enzymes that have a vitB12 cofactor [36]. The ability to synthesize cobamides *de novo* involves approximately 30 steps [37] and is found in ~37% of prokaryotes [36]. No known eukaryote presents evidence for cobamide *de novo* biosynthesis. Nonetheless, some encode a few enzymes that modify cobalamin or use it as cofactor [38]. Only four of the latter are known [27,38]. First, methylmalonyl-CoA mutase (MCM) and methylmalonyl-CoA epimerase (MCE) are involved in odd-chain fatty acid metabolism in the mitochondria of animals. Second, B12-dependent ribonucleotide reductase (RNR) is found in a few mostly anaerobic eukaryotes. There are both B12-independent and B12-dependent forms of ribonucleotide reductase (RNR) (type I & III RNR are B12 independent, type II is B12 dependent), involved in deoxyribose biosynthesis [39,40]. Most eukaryotes have the type I isoform, but this isoform is oxygen sensitive, so several anaerobes have acquired vitB12-dependent RNR from bacteria, viruses, or even other eukaryotes on several occasions [27,41]. Third, two isozymes of methionine synthase are similarly found. MetH is B12-dependent and is found in animals, while an alternative B12-independent form of methionine synthase (*metE*) is found in land plants and fungi. Based on the presence of *metH* and the absence of functional *metE*, approximately half of all cultured eukaryotic algal species from marine and freshwater environments are predicted to require exogenously produced corrinoids for growth (Croft et al. 2005; Helliwell et al. 2011). This property appeared not to be monophyletic but rather scattered in the tree. MetH has a higher catalytic rate compared to MetE [42], factors that might explain why it can be advantageous to possess *metH* despite its dependency to vitB12. Several studies suggest that vitB12 might be exchanged between prokaryotic producers and B12 auxotroph algae, which would provide the bacteria with photosynthate [43,44]. Other studies predict that corrinoid requirements can be fulfilled through indirect production and release into the water column upon death and cell lysis [45].

The initial observations that (i) *Anaeramoeba* have acquired from bacteria vitB12-dependent metH and vitB12-dependent RNR (and that the vitB12-independent isozymes are absent) and (ii) that the *Desulfobacter* symbionts encode some cobalamin biosynthesis genes led us to wonder if *Anaeramoeba* have additional enzymes requiring vitB12 and if vitB12 could be exchanged between *Anaeramoeba* and *Desulfobacter*.

In order to better understand the vitB12 picture of the *Anaeramoeba* system, we:

(A) scanned the *Desulfobacter* symbionts associated with *A. ignava* and *A. flamelloides* genomes for vitB12 synthesis and vitB12 uptake genes to confirm that they can synthesize it *de novo*
(B) scanned the *Anaeramoeba* genomes for
- (i) additional vitB12-dependent enzymes, besides MetH and RNR.
- (ii) cobalamin synthesis genes. Indeed the acquisition from bacteria of enzymes involved in the final steps of cobalamin synthesis was described in some diatoms [46].
- (iii) vitB12 import enzymes.

(A) The *Desulfobacter* symbionts associated with *A. ignava* and *A. flamelloides* (BUSSELTON2 and SCHOONER1) seems to encode a full pathway for anaerobic vitB12

synthesis including: several cobalt import proteins, ALA synthesis, synthesis of the tetrapyrrole precursor, corrin ring synthesis, adenosylation, nucleotide loop assembly, aminopropanol linker, synthesis of lower ligand DMB, addition of alpha-ribazol phosphate. The only enzyme that is not found is CobC which is responsible for the very last step in which the phosphate is removed from the lower ligand alpha-ribazol after the later was added to the corrin ring. None of CobZ [47], CblX, CblY and CblZ, [48], which are nonorthologous replacements of CobC were found either. However, this step might not be needed for cobalamin use in *Desulfobacter* or this step might occur through an undescribed phosphatase as (i) *Desulfobacterium autotrophicum*, another member of *Desulfobacteraceae* was shown experimentally to produce cobalamin but not to encode CobC either [36]; (ii) in some archaea (e.g., *Halobacterium*), the phosphatase has not been identified [49] suggesting that other non orthologous phosphatase are yet to be described.
Despite likely producing cobalamin *de novo*, the *Desulfobacter* symbionts also have several enzymes involved in the uptake of cobalamin related to BtuB. The coexistence of the *de novo* synthesis pathway and uptake proteins is known to occur in other vitB12 producers as well [36]. The *Desulfobacter* symbionts have several vitB12-dependent enzymes so do not produce cobalamin only for *Anaeramoeba*.

(B)
(i) *A. ignava* and *A. flamelloides* have orthologs of the three vitB12-dependent enzymes found in eukaryotes. As previously mentioned, the type II RNR and metH were acquired from bacteria, while methylmalonyl-CoA mutase and methylmalonyl-CoA mutase-associated GTPase MeaB (MeaB is a small G-protein involved in loading coenzyme B_{12} to MCM [50] are eukaryotic.
Interestingly, *A. flamelloides* strains BUSSELTON2 and SCHOONER1 appear to possess two additional LGT-derived vitB12-dependent enzymes, neither of which have been described to occur in eukaryotes.

The first is a B12-dependent reductive dehalogenase, the presence of which opens the possibility that *A. flamelloides* might be able to metabolize halogenated compounds. The *A. flamelloides* enzymes are related to archaeal proteins. The presence of this dehalogenase in members of the *Lokiarchaeota* and *Thorarchaeota* was recently highlighted [51,52]. The *Desulfobacter* symbionts do not encode the orthologous gene but have the related vitB12-dependent epoxyqueusine reductase.

The second enzyme is B12-dependent Ethanol ammonia-lyase, EutBC. Ethanolamine serves as a source of carbon and nitrogen for a variety of bacteria [53–55]. EutBC is the core enzyme in ethanolamine utilisation. It breaks down ethanolamine into acetaldehyde and ammonia [55].

Crucially, the *Desulfobacter* symbionts associated with *A. flamelloides* appear not to have EutBC while the symbiont associated with *A. ignava* encodes it. A similar pattern is found for EutT, which is the corrinoid-adenosyltransferase dedicated to produce the vitB12 cofactor ethanolamine for ethanolamine ammonia lyase, and for the acetaldehyde dehydrogenase EutE. After the break down of ethanolamine by EutBC, EutE converts the acetaldehyde intermediate to acetyl-CoA, which enters the carbon pool of the cell. This suggests that the latter do rely less than the former on *Anaeramoeba* to feed the Wood-Ljungdahl pathway with acetate. This observation is in line with a longer association between *A. flamelloides* and its symbiont compared with the *A. ignava*/*Desulfobacter* system.
The ethanolamine utilisation genes in bacteria are organised in operons that are variable in length, ranging from the core EutB and EutC only in some species to up to 17 genes in other [55]. Interestingly, *Anaeramoeba* Ethanolamine ammonia lyase consist of a fused EutB and EutC as found in some *Deltaproteobacteria* [55] while the *A. ignava* symbiont has open reading frame for EutB and one for EutC.

Neither *A. flamelloides* BUSSELTON2 nor *A. flamelloides* SCHOONER1 have EutE, suggesting that the Ethanolamine ammonia lyase is not devoted to produce acetate. Rather, ethanolamine might be used as a source of nitrogen. Interestingly, *A. flamelloides* have the alcohol dehydrogenase EutG that converts acetaldehyde into ethanol (also found in the three symbionts). In *Anaeramoeba*, the enzyme appears to have been acquired from archaea.

(ii)

- The eukaryotic cobalamin adenosyl transferase is found in the three Anaeramoebae. This enzyme catalyzes the conversion of cobalamin into adenosylcobalamin, the form used as a cofactor by Methylmalonyl-CoA mutase.
- *A. ignava* have laterally acquired an alpha-ribazol phosphatase (CobC), corresponding to the very last step of cobalamin synthesis (that was not found in the symbionts). The lateral acquisition of some steps of cobalamin synthesis including cobC has occurred in a subset of diatoms auxotroph for vitB12 [46]. We have discussed the fact that the absence of cobC in the *Desulfobacter* might not be crucial given that cobC is not found in the symbionts. Consequently, and because *A. flamelloides* is lacking cobC, it is unclear what cobC is doing in *A. ignava*. One possibility is that the cobalamin is imported in *A. ignava* with the phosphate attached to the lower ligand and that the removal of the phosphate happens in the eukaryote.

(iii)

- none of the described prokaryotic cobalamin transporters described in [48,56–58] were found in *Anaeramoeba*.
- nor was the diatom-like cobalamin binding protein CBA1 [59].

However, (i) not much is known about cobalamin import in eukaryotes outside of metazoan [58,60], and (ii) the extreme proximity between the symbiont and *Anaeramoeba* likely favorize the import of vitB12 produced by the symbiont by *Anaeramoeba*.

The mechanism by which corrinoids are released from corrinoid-producing microbes into the environment is unclear. As yet, no active means of corrinoid export has been identified [61].

Supplementary Note 4

Anaeramoeba symbionts and their genomes:

Enrichment of amoebae led to enrichment of Desulfobacteraceae

16S rDNA sequencing data of replicate enrichments confirmed the metagenomic sequencing results with distinct profiles in the culture supernatant and the amoeba-enriched cell material (Figure S6B,C). *Bacteroidales and Pseudoalteromonadaceae* were dominant in the culture supernatant of *A. ignava,* whereas *Vibrio* was most abundant in the *A. flamelloides* sample. These lineages were depleted in the amoeba-enriched samples (Figure S6B,C). The obtained relative level of enrichment for the *Desulfobacteraceae* lineage was higher in *A. flamelloides* (Figure S6B) than in *A. ignava* (Figure S6C). We mapped long-reads from individual sequencing runs representing distinct DNA preparations back to the assembled contigs to estimate the respective abundance of each organism from the average contig depth coverage. The data were normalized to the coverage of the *Anaeramoeba* nuclear genome to establish the number of prokaryotic cells / amoeba genome equivalents (Figure S21).

IS element activity in the A. flamelloides *symbionts*

The number of IS elements recorded in SRBs is variable, but some free-living *Desulfobacteraceae* genomes encode high numbers (for example, 130 copies in *Desulfobacula toluolica* Tol2). We found high numbers of transposable elements in both of the *A. flamelloides* symbionts (> 700 copies/genome) (Supplementary Data 2), while the BMAN symbiont encodes 60 IS elements, a comparable number to that of free-living species. The IS elements showed no strong evidence of clustering overall (Figure S11AB) but we found that >200 IS elements were close (<1500 bp) to edges of syntenic blocks, indicating the frequent genome rearrangements might indeed be connected to IS element activity (Figure S11C,D). Many of the high-copy-number IS elements had a complete transposase indicating that they were recently active. The two most abundant IS elements in both genomes were IS256 (Sym_BUSS2 172 copies, Sym_SCH1 194 copies) and IS4_ssgr_IS4Sa (Sym_BUSS2 129 copies, Sym_SCH1 185 copies). In total we detected 13 families in Sym_SCH1 and 14 families in Sym_BUSS2, and about half of them, six families in Sym_SCH1 and seven families in Sym_BUSS2, were considered abundant (>40 copies) (Supplementary Data 2).

Pseudogene curation in A. flamelloides *symbionts*

Automatic methods for pseudogene detection (PGAP and pseudofinder[62]) both indicated an elevated number of pseudogene candidates in the Sym_BUSS2 and Sym_SCH1 genomes compared to Sym_BMAN (Table S2), however the detected numbers were starkly different between the two methods. Pseudogenes were manually curated for Sym_BUSS2 and Sym_SCH1 guided by synteny evidence and the automatic pseudogene predictions.

A. flamelloides *symbionts have lost flagella and have impaired cell wall assembly machinery*

Flagella are crucial to many cells found in fluctuating environments but may become dispensable in stable environments encountered by symbionts [63]. Sym_BMAN shows no degradation of its flagellar operon with 54 full-length genes and no IS element insertions. Strikingly Sym_SCH1 and Sym_BUSS2 both have a ten gene deletion that includes (*flgB*, *flgC*, *fliE*, *fliF*) and a 20 kb inversion at the 3'end compared to Sym_BMAN. Their flagellar loci are

further degraded by pseudogenization (Sym_BUSS2, 14 Ψ / 44 genes; Sym_SCH1, 7 Ψ / 22 genes) and multiple IS element insertions (Sym_BUSS2, 4 insertions; Sym_SCH1, 3 insertions). In addition, Sym_SCH1 has a 27 kbp deletion that deletes 24 genes (Figure S13). We conclude that both *A. flamelloides* symbionts have lost the ability to produce flagella, whereas the *A. ignava* symbiont still retains the capacity to produce flagella.

The three symbionts retain the machinery to produce core lipid A and Kdo. However, Sym_BUSS2 and Sym_SCH1 have pseudogenes of *waaL* that encode O-antigen ligase. Sym_BUSS2 further has a defunct copy of *wzyC*, which encodes O-antigen polymerase. *A. flamelloides* symbionts also might be impaired in decorating lipid A with O-antigen. Having reduced O-antigen on lipid A decrease the permeability barrier at the outer membrane [64], and it could additionally lead to better adherence between host and symbiont.

Adherence

Type IV pili help pathogens such as *Pseudomonas aeruginosa* to colonize a host. The *pilMNOPQ* loci encode factors important for both T4P assembly and twitching motility. The Sym_BMAN and *D. toluolica* loci are syntenic and show no evidence of degradation. The Sym_BUSS2 and Sym_SCH1 loci both have a pseudogenized *pilQ* gene. The gene is frame-shifted in both strains, with Sym_SCH1 having a further two IS element insertions. Further, Sym_BUSS2 and Sym_SCH1 have pseudogenized copies of *pilB* and one of the two copies of *pilU/T*. Sym_BMAN further encodes an incomplete Tad (tight adherence) gene cluster which is not present in Sym_BUSS2 and Sym_SCH1. This gene cluster has shown to be essential for colonization of surfaces by the human pathogen *Actinobacillus actinomycetemcomitans* [65]. Components of the Tad cluster code for a secretion system that exports and assembles bundled Flp pili (fibrils).

The A. ignava *symbionts encode type VI secretion systems and anti-viral defenses*

We detected the presence of several potential protein secretion systems in the symbionts. All three are predicted to encode the Sec-SRP general secretion system while the twin-arginine translocation (TAT) pathway appears compromised in Sym_BUSS2 and Sym_SCH1 by the pseudogenization of *tatB*. Sym_BMAN encodes a Type II secretion and a type VI secretion system (T6SS) where the latter consists of two convergent transcription units of 25 genes, with most genes showing evidence of expression (Figure S14). The first transcription unit includes 12 genes (*tssJKLM, tagF*, *tssA*, *tssBC*, *tssEFGH*) that are conserved in most T6SS and two hypothetical proteins. The second transcription unit encodes the two conserved T6SS factors, *vgrG*, *hcp,* and upstream of these; we find a forkhead associated domain (FHA) protein, a TPR repeat protein, a carbohydrate esterase 4 (CE4) superfamily protein. The downstream genes include a DUF4280 family protein previously suggested as potential spike (PAAR) proteins [2]. Directly downstream of *vgrG,* we identified a DUF4123 protein, which is generally encoded upstream of putative T6SS effectors [66]. The four effector candidates encoded between the DUF4123 domain protein and DUF4280 domain protein have no known function, but putative homologs are present in both free-living and bacterial symbiont genomes.

Phage and plasmid infections can be controlled by a growing list of defense systems and multiple systems are usually found in bacterial and archaeal genomes [67]. We investigated the repertoire of phage and plasmid defense systems in the symbionts and compared it to repertoires of free-living *Desulfobacteraceae* (same set of organisms as used in the phylogenetic analysis in Figure 3M) using the tool PADLOC [67]. We detected very few defense components (Sym_SCH1 – 3 systems, Sym_BUSS2 – 2 systems) in the *A. flamelloides* symbionts (Supplementary Data 10). This is in contrast to most free-living *Desulfobacteraceae* which tend to encode multiple defense systems (average 13.9, MAGS excluded 14.3). Upon close inspection two each of the detected systems of Sym_BUSS2 and Sym_SCH1 encode pseudogenized CRISPR systems of type I-C. The leading *cas2* and terminating *cas3”* genes

are truncated, and no associated CRISPR protospacer array was identified. Sym_BMAN appears more similar to free-living *Desulfobacteraceae* in that it encodes 9 defense systems including an intact type I-F CRISPR system with an array of 25 spacers. None of the spacers were found to be self-matching or belong to known phages or plasmids.

Membrane complexes and transporters

Several highly conserved membrane complexes direct electron transfers in DSR organisms (Pereira 2008). In Sym_BUSS2, we detected high expression (dsrJ – 768 RPKM, dsrO – 751 RPKM, dsrP – 633 RPKM, dsrK – 536 RPKM, dsrM – 390 RPKM) of the DsrMJKOP transmembrane electron carrier complex that shuttles electrons to sulfite reductase. By contrast, this membrane complex is not as prominently expressed in Sym_BMAN (dsrJ – 136 RPKM, dsrO – 109 RPKM, dsrP – 107 RPKM, dsrK – 77 RPKM, dsrM – 136 RPKM). The Rnf respiratory membrane complex is highly expressed in both symbionts. This complex acts a H+/Na+ channel that oxidizes reduced ferredoxin and reduces NAD+ coupled to ion transport. The most highly expressed gene in both Sym_BMAN and Sym_BUSS2 is an outer membrane porin of unknown function. The outer membrane protein OmpA is particularly highly expressed in Sym_BUSS2. In contrast, an Oxalate:formate antiporter is the second highest expressed transporter in Sym_BMAN. Additional transporters with prominent expression include nickel-, ammonium (Sym_BUSS2) and molybdenum transporters (Supplementary Data 3).

Amino acids metabolism in the symbionts

All the three symbionts, Sym_BMAN, Sym_BUSS2 and Sym_SCH1, maintain the ability to synthesize the 20 standard amino acids. Complete pathways were predicted for 16 amino acids (L-arginine, L-asparagine, L-cysteine, L-glutamine, L-glycine, L-isoleucine, L-leucine, L-lysine, L-methionine, L-phenylalanine, L-proline, L-serine, L-threonine, L-tryptophan, L-tyrosine, L-valine) as well as chorismate biosynthesis (Supplementary Data 11). Histidine synthesis contains two gaps where hisI and hisN activities are unaccounted for. These are known pathway gaps known to be present in free-living *Desulfobacteraceae* species (*Desulfobacter vibrioformis* DSM 8776, *Desulfobacula toluolica* Tol2) known to perform L-histidine biosynthesis. L-alanine can be synthesized from cysteine, L-lysine is made using the diaminopimelate-aminotransferase variant pathway, L-proline can be made from glutamate or ornithine and L-glycine from threonine and serine. Isoleucine biosynthesis is also possible via ferredoxin-dependent reductive carboxylation of propanoyl-CoA to 2-oxobutanoate propanoate. Multiple transaminases of different classes are present to likely serve the need for synthesis of L-alanine, L-aspartate and L-glutamate. There are some symbiont-specific differences in the amino acid metabolism;

The *A. flamelloides* symbionts differ in their amino acid pathways. For example, 8 out of 29 Sym_SCH1 and 5 out of 37 Sym_BUSS2 specific proteins respectively function in amino acid pathways (Supplementary Data 11). Sym_SCH1 encodes several additional enzymes involved in Methionine biosynthesis (Homoserine O-acetyltransferase (EC 2.3.1.31), O-acetylhomoserine sulfhydrylase (EC 2.5.1.49), O-succinylhomoserine sulfhydrylase (EC 2.5.1.48). The enzyme complement of the arginine and ornithine degradation pathway is also variable between the two symbionts. Sym_BUSS2 and Sym_SCH1 can assimilate ammonia via glutamate-ammonia ligase and glutamate synthase. Sym_BUSS2 encodes threonine hydratase to convert threonine to isoleucine via 2-oxobutanoate. Sym_BUSS2 and Sym_SCH1 encode the L-arginine degradation pathway that proceeds via arginase.

The amino acid metabolic pathways are more extensive in Sym_BMAN compared to the *A. flamelloides* symbionts. Sym_BMAN encodes 36 and 37 additional genes in amino acid biosynthesis pathways compared to Sym_SCH1 and Sym_BUSS2 respectively (Supplementary Data 11). For example, this includes expanded capabilities in alanine, glycine, arginine and polyamine biosynthesis. They also encode a bigger set of genes in ammonia

assimilation via glutamate and aspartate as well as in proline uptake. Sym_BMAN can make L-asparagine from aspartic acid and L-homocysteine from L-homoserine. Genes for utilization of histidine as well as arginine and ornithine are also present. We detected inactivated copies histidine ammonia-lyase and urocanate hydratase, involved in histidine catabolism, in Sym_BUSS2 and Sym_SCH1.

Selenocysteine and pyrrolysine

The ability to make selenocysteine and selenoproteins is not a universal feature and it may be acquired by lateral transfer events [68]. Selenoproteins are well represented in organisms inhabiting certain anaerobic environments where their unique properties can be advantageous [69–71]. Selenocysteine might be better able to withstand oxidation better than cysteine and could offer an advantage in fluctuating anaerobic environments [72]. The sulfate-oxidizing and sulfate-reducing symbionts of the gutless worm *Olavius algarvensis* was previously shown to encode a large complement of both selenoproteins and pyrrolysine-containing proteins [73]. The *Anaeramoeba* symbiont genomes encode many selenoproteins (BUSS2 – 19, Sch – 20, BMAN – 25). Selenocysteine is predicted to be synthesized from Se^0 and incorporated in proteins in all three *Anaeramoeba* symbionts using the standard bacterial pathway. Enzymes predicted to use selenocysteine includes Selenide, water dikinase and prominent redox-proteins (CoB--CoM-reducing hydrogenase (Sec) delta subunit, DsrK-like, NAD-dependent formate dehydrogenase alpha subunit, Uptake hydrogenase large subunit).

Pyrrolysine is known as the 22nd amino acid that can be incorporated into proteins during translation. Sym_BMAN encodes the necessary enzymatic pathway (proline 2-methylase (pylB), pyrrolysine synthetase (pylC), proline reductase (pylD), pyrrolysine-tRNA(Pyl) ligase large and small subunits) to synthesize and incorporate pyrrolysine into proteins. Trimethylamine--corrinoid protein Co-methyltransferase (MttB) was found to be have an in-frame stopcodon to guide Pyl-insertion. The target for MttB-directed methylation in Sym_BMAN is unclear since no clear target is present.

Nitrogenase

Nitrogen sources are often limited in microbial systems even though molecular nitrogen is abundant in the atmosphere. A limited number of microbes have the ability to use molecular nitrogen as a nitrogen source using an enzyme called nitrogenase. The exotic molybdenum-iron cluster in nitrogenase is able to fix nitrogen directly into ammonia by ATP expenditure. Sym_BMAN encodes a Mo-nitrogenase and associated genes (*nifH*, *nifD*, *nifN*, *nifB*, *nifV*, 2Fe) that allows the fixation of N_2 into ammonia using with the expenditure of ATP.

DNA repair systems of the symbionts

The genome degeneration in the *A. flamelloides* symbionts could indicate ineffective DNA repair systems. We failed to find genes encoding endonuclease IV that binds to and excises apurinic-apyrimidinic sites and excises them to direct DNA repair in both *A. flamelloides* symbionts. This protein is found in Sym_BMAN and free-living members of *Desulfobacteraceae* (Supplementary Data 12). We also found pseudogenized copies of deoxyribodipyrimidine photo-lyase in the *A. flamelloides* symbionts making them less able to respond to UV-induced stress. However, this gene appears to be missing from other *Desulfobacteraceae* genomes including Sym_BMAN (Supplementary Data 12). Several genes whose products are prominent members in DNA replication and repair pathways (*dinB*, *uvrD, and dnaE*) were found to be pseudogenized in Sym_BUSS2 and Sym_SCH1 (Supplementary Data 1). The significance of these gene losses is not clear since most of these are multi-copy genes, and their function is likely to be compensated by the presence of a second redundant copy. Mutator phenotypes have been recovered in natural settings as well as in lab evolution experiments where they can have a selective advantage in finding adaptive mutations under relaxed selective conditions. Mutators are predicted to be favored during nascent symbioses

where the chance of essential genes being perturbed is lower than in later stages in symbiont evolution [74].

Genome erosion buffering and symbiont stability

GroEL/S is believed to buffer the deleterious changes that accrue in symbiont proteins as a result of genome erosion. Constitutive overexpression of GroEL/S is regarded as a critical factor in stabilizing endosymbionts in a wide range of insect symbioses [75] and has been shown to buffer mutations in mutator strains of *E. coli* [76]. In organisms experiencing genome erosion such as *Buchnera aphidicola* GroEL/S can amount to 10% of the total cellular protein [77]. Interestingly we found that GroEL/S are highly expressed (Ranked 24 – 2044 RPKM and 39 – 1353 RPKM) in Sym_BUSS2 while moderately expressed in Sym_BMAN (Ranked 436 – 167 RPKM and 572 – 133 RPKM) in line with their respective degrees of genome erosion (Supplementary Data 3). In addition to this, the chaperone DnaK in Sym_BUSS2 is highly expressed (rank 93 – 663 RPKM) whereas it being expressed at a low level (rank 1734 - 43 RPKM) in Sym_BMAN (Supplementary Data 3).

We found additional cellular defense systems in the symbionts to be highly activated. Superoxide reductase and Rubredoxin defends the cell against highly reactive and toxic superoxide radicals (O_2^-) that is converted into hydrogen peroxide. Both genes are highly expressed in Sym_BUSS2 (rank 27 – 1959 RPKM and rank 66 – 524 RPKM respectively) and in Sym_BMAN (rank 27 – 1386 RPKM and 110 – 496 RPKM) (Supplementary Data 3). The less toxic hydrogen peroxide is detoxified by the action of Rubrerythrin. The symbionts encode several copies of Rubrerythrin (8 copies in Sym_BMAN and 6 copies in Sym_BUSS2). Several rubrerythrin genes in Sym_BUSS2 show high expression and the most highly expressed rubrerythrin gene copy (rank 25 – 2043 RPKM) is transcribed from the same operon as the Per2 peroxide stress regulator which is likewise highly expressed (rank 84 – 690 RPKM). In addition, Sym_BMAN shows high expression of Dodecin (rank 12 – 2136 RPKM) that can neutralize flavins and shield the cell from high flavin reactivity that can be toxic if they accumulate in an uncontrolled way. Several cold-shock proteins and additional stress sensors such as Ribosome hibernation protein YhbH, the previously mentioned Per2 peroxide stress regulator and Phage shock protein A are highly expressed. The abundant expression of cold-shock proteins might be related to the low temperatures the cells are exposed to during cell harvest. The abundant expression of stress coping genes indicates that the symbionts, and especially Sym_BUSS2, is highly active in countering stress perhaps related to the accumulation of slightly deleterious protein coding alleles.

Supplementary Note 5

FIB-SEM analyses:

FIB-SEM general volume statistics

Visualization of segmentations are shown in Figure 2 and Figure S3. The cell contained a single drop-shaped nucleus that occupies 2.80 % of the cell volume. The cell is bounded by a plasma membrane (0.91% of cell volume). Microtubules radiate outwards from the single acentriolar centrosome at the ventral side of the cell. The symbiont and hydrogenosomes form a densely packed mass close to but not in direct contact with nucleus. There are 185 symbionts (183 intact and 2 degenerating) that account for 7.95 % of the total cell volume. The symbionts are housed in a membrane system whose volume was 1.92% of the cell volume. The hydrogenosomes consist of individual organelles but many form a reticulated network that altogether encompass 4.85% of the cell volume. Abundant dense granules (5.40%) of unknown composition are found in the cytoplasm. Dense granules of this type have previously not been observed in Anaeramoebae and might be due to a difference in culturing conditions (ASW+LB instead of SW802 as growth media). We reconstructed additional prokaryotes (1.21%) in the volume that did not have a tight connection to hydrogenosomes and whose enclosing membranes were less structured than those harboring the symbiont population. These cells were invariably inside of membrane vacuoles and some were in the process of being digested.

Tracing of symbiosome subcompartment connections in the FIB-SEM reconstruction

Membrane connections between symbiosome subcompartments were manually traced in all three dimensions (XY, ZY, ZX planes) and annotated in MIB (Figure S4 and Movie S2). Symbiosome subcompartments were sorted into different contiguous groups based on the identified membrane contacts yielding fifteen subcompartments with multiple symbionts and 25 subcompartments with individual symbionts without traceable contact to another symbiosome subcompartment (Figure S5). Two symbionts that are degenerating were not considered in the analysis. The total number of symbionts in the multi-symbiont subcompartments were 158 cells (105, 10, 6, 5, 5, 5, 4, 3, 3, 2, 2, 2, 2, 2, 2) meaning that 86% of the symbionts were connected to least one other cell. Two of the compartments, containing 105 and three symbionts, respectively, were connected to the outside media making for 108 that were in direct contact to the outside media; 108 symbionts were thus inferred to be in direct contact to the outside media (Figure 2H,I, Figure S5I-L, Movie S2).

Supplementary references

1. Minkin, I., Pham, H., Starostina, E., Vyahhi, N. & Pham, S. C-Sibelia: an easy-to-use and highly accurate tool for bacterial genome comparison. *F1000Research* **2**, 258 (2013).
2. Lays, C., Tannier, E. & Henry, T. *Francisella* IglG protein and the DUF4280 proteins: PAAR-like proteins in non-canonical Type VI secretion systems? *Microb. Cell* **3**, 576–578 (2016).
3. Jerlström-Hultqvist, J. *et al.* Genome analysis and comparative genomics of a *Giardia intestinalis* assemblage E isolate. *BMC Genomics* **11**, 543 (2010).
4. Gentekaki, E. *et al.* Extreme genome diversity in the hyper-prevalent parasitic eukaryote *Blastocystis*. *PLOS Biol.* **15**, e2003769 (2017).
5. Crooks, G. E., Hon, G., Chandonia, J.-M. & Brenner, S. E. WebLogo: A Sequence Logo Generator: Figure 1. *Genome Res.* **14**, 1188–1190 (2004).
6. Burset, M. Analysis of canonical and non-canonical splice sites in mammalian genomes. *Nucleic Acids Res.* **28**, 4364–4375 (2000).
7. Pucker, B. & Brockington, S. F. Genome-wide analyses supported by RNA-Seq reveal non-canonical splice sites in plant genomes. *BMC Genomics* **19**, 980 (2018).
8. Kupfer, D. M. *et al.* Introns and Splicing Elements of Five Diverse Fungi. *Eukaryot. Cell* **3**, 1088–1100 (2004).
9. Turunen, J. J., Niemelä, E. H., Verma, B. & Frilander, M. J. The significant other: splicing by the minor spliceosome: Splicing by the minor spliceosome. *Wiley Interdiscip. Rev. RNA* **4**, 61–76 (2013).
10. Ban, N. *et al.* A new system for naming ribosomal proteins. *Curr. Opin. Struct. Biol.* **24**, 165–169 (2014).
11. Chan, P. P. & Lowe, T. M. tRNAscan-SE: Searching for tRNA Genes in Genomic Sequences. in *Gene Prediction* (ed. Kollmar, M.) vol. 1962 1–14 (Springer New York, New York, NY, 2019).

12. Filipovska, A. & Rackham, O. Specialization from synthesis: How ribosome diversity can customize protein function. *FEBS Lett.* **587**, 1189–1197 (2013).
13. Segev, N. & Gerst, J. E. Specialized ribosomes and specific ribosomal protein paralogs control translation of mitochondrial proteins. *J. Cell Biol.* **217**, 117–126 (2018).
14. Ghulam, M. M., Catala, M. & Abou Elela, S. Differential expression of duplicated ribosomal protein genes modifies ribosome composition in response to stress. *Nucleic Acids Res.* **48**, 1954–1968 (2020).
15. Stairs, C. W. *et al.* Anaeramoebae are a divergent lineage of eukaryotes that shed light on the transition from anaerobic mitochondria to hydrogenosomes. *Curr. Biol.* **31**, 5605-5612.e5 (2021).
16. Aramaki, T. *et al.* KofamKOALA: KEGG Ortholog assignment based on profile HMM and adaptive score threshold. *Bioinformatics.* **36**, 2251–2252 (2020).
17. Semsey, S., Krishna, S., Sneppen, K. & Adhya, S. Signal integration in the galactose network of *Escherichia coli*. *Mol. Microbiol.* **65**, 465–476 (2007).
18. Žárský, V. *et al.* The *Mastigamoeba balamuthi* Genome and the Nature of the Free-Living Ancestor of *Entamoeba*. *Mol. Biol. Evol.* **38**, 2240–2259 (2021).
19. Alsmark, C. *et al.* Patterns of prokaryotic lateral gene transfers affecting parasitic microbial eukaryotes. *Genome Biol.* **14**, R19 (2013).
20. Jiménez-González, A., Xu, F. & Andersson, J. O. Lateral Acquisitions Repeatedly Remodel the Oxygen Detoxification Pathway in Diplomonads and Relatives. *Genome Biol. Evol.* **11**, 2542–2556 (2019).
21. Slamovits, C. H. & Keeling, P. J. Pyruvate-Phosphate Dikinase of Oxymonads and Parabasalia and the Evolution of Pyrophosphate-Dependent Glycolysis in Anaerobic Eukaryotes. *Eukaryot. Cell* **5**, 148–154 (2006).
22. Mertens, E. ATP versus pyrophosphate: glycolysis revisited in parasitic protists. *Parasitol. Today* **9**, 122–126 (1993).

23. Baum, K. F., Berens, R. L., Marr, J. J., Harrington, J. A. & Spector, T. Purine deoxynucleoside salvage in *Giardia lamblia*. *J. Biol. Chem.* **264**, 21087–21090 (1989).
24. Xu, F. *et al.* On the reversibility of parasitism: adaptation to a free-living lifestyle via gene acquisitions in the diplomonad *Trepomonas* sp. PC1. *BMC Biol.* **14**, 62 (2016).
25. Xu, F. *et al.* The compact genome of *Giardia muris* reveals important steps in the evolution of intestinal protozoan parasites. *Microb. Genomics* **6**, (2020).
26. Lundin, D., Gribaldo, S., Torrents, E., Sjöberg, B.-M. & Poole, A. M. Ribonucleotide reduction - horizontal transfer of a required function spans all three domains. *BMC Evol. Biol.* **10**, 383 (2010).
27. Crona, M. *et al.* A Rare Combination of Ribonucleotide Reductases in the Social Amoeba *Dictyostelium discoideum*. *J. Biol. Chem.* **288**, 8198–8208 (2013).
28. Leger, M. M., Eme, L., Hug, L. A. & Roger, A. J. Novel Hydrogenosomes in the Microaerophilic Jakobid *Stygiella incarcerata*. *Mol. Biol. Evol.* **33**, 2318–2336 (2016).
29. Stairs, C. W. *et al.* Oxygen induces the expression of invasion and stress response genes in the anaerobic salmon parasite *Spironucleus salmonicida*. *BMC Biol.* **17**, 19 (2019).
30. Bloch, K. The Biological Synthesis of Cholesterol. *Science* **150**, 19–28 (1965).
31. Takishita, K. *et al.* Lateral transfer of tetrahymanol-synthesizing genes has allowed multiple diverse eukaryote lineages to independently adapt to environments without oxygen. *Biol. Direct* **7**, 5 (2012).
32. Bouwknegt, J. *et al.* A squalene–hopene cyclase in *Schizosaccharomyces japonicus* represents a eukaryotic adaptation to sterol-limited anaerobic environments. *Proc. Natl. Acad. Sci.* **118**, e2105225118 (2021).
33. Alauzet, C., Aujoulat, F., Lozniewski, A. & Marchandin, H. A sequence database analysis of 5-nitroimidazole reductase and related proteins to expand knowledge on enzymes responsible for metronidazole inactivation. *Anaerobe* **55**, 29–34 (2019).

34. Pal, D. *et al. Giardia* , *Entamoeba* , and *Trichomonas* Enzymes Activate Metronidazole (Nitroreductases) and Inactivate Metronidazole (Nitroimidazole Reductases). *Antimicrob. Agents Chemother.* **53**, 458–464 (2009).

35. Bradic, M. *et al.* Genetic Indicators of Drug Resistance in the Highly Repetitive Genome of *Trichomonas vaginalis*. *Genome Biol. Evol.* **9**, 1658–1672 (2017).

36. Shelton, A. N. *et al.* Uneven distribution of cobamide biosynthesis and dependence in bacteria predicted by comparative genomics. *ISME J.* **13**, 789–804 (2019).

37. Warren, M. J., Raux, E., Schubert, H. L. & Escalante-Semerena, J. C. The biosynthesis of adenosylcobalamin (vitamin B12). *Nat. Prod. Rep.* **19**, 390–412 (2002).

38. Orłowska, M., Steczkiewicz, K. & Muszewska, A. Utilization of Cobalamin Is Ubiquitous in Early-Branching Fungal Phyla. *Genome Biol. Evol.* **13**, evab043 (2021).

39. Hamilton, F. D. Ribonucleotide Reductase from *Euglena gracilis*. *J. Biol. Chem.* **249**, 4428–4434 (1974).

40. Carell, E. F. & Seeger, J. W. Ribonucleotide reductase activity in vitamin B12-deficient *Euglena gracilis*. *Biochem. J.* **188**, 573–576 (1980).

41. Helliwell, K. E., Wheeler, G. L., Leptos, K. C., Goldstein, R. E. & Smith, A. G. Insights into the Evolution of Vitamin B12 Auxotrophy from Sequenced Algal Genomes. *Mol. Biol. Evol.* **28**, 2921–2933 (2011).

42. Gonzalez, J. C., Banerjee, R. V., Huang, S., Sumner, J. S. & Matthews, R. G. Comparison of cobalamin-independent and cobalamin-dependent methionine synthases from *Escherichia coli*: two solutions to the same chemical problem. *Biochemistry* **31**, 6045–6056 (1992).

43. Croft, M. T., Lawrence, A. D., Raux-Deery, E., Warren, M. J. & Smith, A. G. Algae acquire vitamin B12 through a symbiotic relationship with bacteria. *Nature* **438**, 90–93 (2005).

44. Grant, M. A. A., Kazamia, E., Cicuta, P. & Smith, A. G. Direct exchange of vitamin B12 is demonstrated by modelling the growth dynamics of algal-bacterial cocultures. *ISME J.* **8**, 1418–1427 (2014).
45. Droop, M. R. Vitamins, phytoplankton and bacteria: symbiosis or scavenging? *J. Plankton Res.* **29**, 107–113 (2007).
46. Vancaester, E., Depuydt, T., Osuna-Cruz, C. M. & Vandepoele, K. Comprehensive and Functional Analysis of Horizontal Gene Transfer Events in Diatoms. *Mol. Biol. Evol.* **37**, 3243–3257 (2020).
47. Zayas, C. L., Woodson, J. D. & Escalante-Semerena, J. C. The *cobZ* Gene of *Methanosarcina mazei* Gö1 Encodes the Nonorthologous Replacement of the α-Ribazole-5′-Phosphate Phosphatase (CobC) Enzyme of *Salmonella enterica*. *J. Bacteriol.* **188**, 2740–2743 (2006).
48. Rodionov, D. A., Vitreschak, A. G., Mironov, A. A. & Gelfand, M. S. Comparative Genomics of the Vitamin B12 Metabolism and Regulation in Prokaryotes. *J. Biol. Chem.* **278**, 41148–41159 (2003).
49. Escalante-Semerena, J. C. Conversion of Cobinamide into Adenosylcobamide in Bacteria and Archaea. *J. Bacteriol.* **189**, 4555–4560 (2007).
50. Padovani, D. & Banerjee, R. A G-protein editor gates coenzyme B_{12} loading and is corrupted in methylmalonic aciduria. *Proc. Natl. Acad. Sci.* **106**, 21567–21572 (2009).
51. Manoharan, L. *et al.* Metagenomes from Coastal Marine Sediments Give Insights into the Ecological Role and Cellular Features of *Loki* - and *Thorarchaeota*. *mBio* **10**, e02039-19 (2019).
52. Spang, A. *et al.* Proposal of the reverse flow model for the origin of the eukaryotic cell based on comparative analyses of Asgard archaeal metabolism. *Nat. Microbiol.* **4**, 1138–1148 (2019).
53. Garsin, D. A. Ethanolamine utilization in bacterial pathogens: roles and regulation. *Nat. Rev. Microbiol.* **8**, 290–295 (2010).

54. Lundgren, B. R., Sarwar, Z., Pinto, A., Ganley, J. G. & Nomura, C. T. Ethanolamine Catabolism in *Pseudomonas aeruginosa* PAO1 Is Regulated by the Enhancer-Binding Protein EatR (PA4021) and the Alternative Sigma Factor RpoN. *J. Bacteriol.* **198**, 2318–2329 (2016).
55. Tsoy, O., Ravcheev, D. & Mushegian, A. Comparative Genomics of Ethanolamine Utilization. *J. Bacteriol.* **191**, 7157–7164 (2009).
56. Degnan, P. H., Barry, N. A., Mok, K. C., Taga, M. E. & Goodman, A. L. Human Gut Microbes Use Multiple Transporters to Distinguish Vitamin B12 Analogs and Compete in the Gut. *Cell Host Microbe* **15**, 47–57 (2014).
57. Rempel, S., Colucci, E., de Gier, J. W., Guskov, A. & Slotboom, D. J. Cysteine-mediated decyanation of vitamin B12 by the predicted membrane transporter BtuM. *Nat. Commun.* **9**, 3038 (2018).
58. Zhang, Y., Rodionov, D. A., Gelfand, M. S. & Gladyshev, V. N. Comparative genomic analyses of nickel, cobalt and vitamin B12 utilization. *BMC Genomics* **10**, 78 (2009).
59. Bertrand, E. M. *et al.* Influence of cobalamin scarcity on diatom molecular physiology and identification of a cobalamin acquisition protein. *Proc. Natl. Acad. Sci.* **109**, (2012).
60. Quadros, E. V., Nakayama, Y. & Sequeira, J. M. The binding properties of the human receptor for the cellular uptake of vitamin B12. *Biochem. Biophys. Res. Commun.* **327**, 1006–1010 (2005).
61. Seth, E. C. & Taga, M. E. Nutrient cross-feeding in the microbial world. *Front. Microbiol.* **5**, (2014).
62. Syberg-Olsen, M. J., Garber, A. I., Keeling, P. J., McCutcheon, J. P. & Husnik, F. Pseudofinder: Detection of Pseudogenes in Prokaryotic Genomes. *Mol. Biol. Evol.* **39**, msac153 (2022).
63. Toft, C. & Fares, M. A. The Evolution of the Flagellar Assembly Pathway in Endosymbiotic Bacterial Genomes. *Mol. Biol. Evol.* **25**, 2069–2076 (2008).

64. Whitfield, C., Williams, D. M. & Kelly, S. D. Lipopolysaccharide O-antigens—bacterial glycans made to measure. *J. Biol. Chem.* **295**, 10593–10609 (2020).

65. Planet, P. J., Kachlany, S. C., Fine, D. H., DeSalle, R. & Figurski, D. H. The Widespread Colonization Island of *Actinobacillus actinomycetemcomitans*. *Nat. Genet.* **34**, 193–198 (2003).

66. Liang, X. *et al.* Identification of divergent type VI secretion effectors using a conserved chaperone domain. *Proc. Natl. Acad. Sci.* **112**, 9106–9111 (2015).

67. Payne, L. J. *et al.* PADLOC: a web server for the identification of antiviral defence systems in microbial genomes. *Nucleic Acids Res.* **50**, W541–W550 (2022).

68. Peng, T., Lin, J., Xu, Y.-Z. & Zhang, Y. Comparative genomics reveals new evolutionary and ecological patterns of selenium utilization in bacteria. *ISME J.* **10**, 2048–2059 (2016).

69. Lacourciere, G. M. & Stadtman, T. C. Catalytic properties of selenophosphate synthetases: Comparison of the selenocysteine-containing enzyme from *Haemophilus influenzae* with the corresponding cysteine-containing enzyme from *Escherichia coli*. *Proc. Natl. Acad. Sci.* **96**, 44–48 (1999).

70. Arnér, E. S. J. Selenoproteins—What unique properties can arise with selenocysteine in place of cysteine? *Exp. Cell Res.* **316**, 1296–1303 (2010).

71. Reich, H. J. & Hondal, R. J. Why Nature Chose Selenium. *ACS Chem. Biol.* **11**, 821–841 (2016).

72. Maroney, M. J. & Hondal, R. J. Selenium versus sulfur: Reversibility of chemical reactions and resistance to permanent oxidation in proteins and nucleic acids. *Free Radic. Biol. Med.* **127**, 228–237 (2018).

73. Zhang, Y. & Gladyshev, V. N. High content of proteins containing 21st and 22nd amino acids, selenocysteine and pyrrolysine, in a symbiotic deltaproteobacterium of gutless worm *Olavius algarvensis*. *Nucleic Acids Res.* **35**, 4952–4963 (2007).

74. McCutcheon, J. P., Boyd, B. M. & Dale, C. The Life of an Insect Endosymbiont from the Cradle to the Grave. *Curr. Biol.* **29**, R485–R495 (2019).

75. Kupper, M., Gupta, S. K., Feldhaar, H. & Gross, R. Versatile roles of the chaperonin GroEL in microorganism-insect interactions. *FEMS Microbiol. Lett.* **353**, 1–10 (2014).

76. Fares, M. A., Ruiz-González, M. X., Moya, A., Elena, S. F. & Barrio, E. GroEL buffers against deleterious mutations. *Nature* **417**, 398–398 (2002).

77. Baumann, P., Baumann, L. & Clark, M. A. Levels of *Buchnera aphidicola* Chaperonin GroEL During Growth of the Aphid *Schizaphis graminum*. *Curr. Microbiol.* **32**, 279–285 (1996).
