# Supplementary material for: A unique symbiosome in an anaerobic single-celled eukaryote
Source: Nat Commun. 2024 Nov 9;15:9726. doi: 10.1038/s41467-024-54102-7 (PMC11550330; doi:10.1038/s41467-024-54102-7)
Supplement: Supplementary file 2 — Description of Additional Supplementary Files [file 41467_2024_54102_MOESM2_ESM.pdf]

Description of Additional Supplementary Files

File Name: Supplementary Movie 1

Description: *Anaeramoeba* symbiosome structure. 3D rendering of the symbiosome in *A. flamelloides* BUSSELTON2 based on FIB-SEM data. (symbiont – blue, hydrogenosome – red, symbiont-membrane – gold, nucleus – purple, plasma membrane – yellow, acentriolar centrosome and microtubules - pink). The animation is available at Figshare (https://doi.org/10.6084/m9.figshare.27108724).

File Name: Supplementary Movie 2

Description: Distribution of symbiosome contact sites. Animation showing the positions of 171 symbiont-to-symbiont connections in FIB-SEM volume of *A. flamelloides* BUSSELTON2. The cell is shown in ventral view (slices 50-1286). Membrane contact positions are indicated by a yellow cross with an extra depth of 20 slices. The contact sites correspond to those cataloged Figure S4 in sequential order. Symbiosome subcompartments in contact with the outside media (light blue), multi-symbiont subcompartments (various colors as in Figure S5), individual subcompartments (white), hydrogenosomes (red), nucleus (purple), plasma membrane (light yellow). Scale bar 1 µm. The animation is available at Figshare (https://doi.org/10.6084/m9.figshare.27108751).

File Name: Supplementary Data 1

Description: Pseudogenes in Sym_BUSS2 and Sym_SCH1. a, Genome coordinates of Sym_BUSS2 pseudogenes. **b,** Genome coordinates of Sym_SCH1 pseudogenes. **c,** Sym_BUSS2 pseudogene annotation by eggnog-mapper 2.1.7. **d,** Sym_SCH1 pseudogene annotation by eggnog-mapper 2.1.7.

File Name: Supplementary Data 2

Description: IS elements in the symbionts. Insertion sequence elements predicted by ISaga in the genomes of Sym_BMAN, Sym_BUSS2 and Sym_SCH1.

File Name: Supplementary Data 3

Description: Metatranscriptomics of Sym_BMAN and Sym_BUSS2. Summary tables of symbiont gene expression in **a,** Sym_BMAN and **b,** Sym_BUSS2 with locus ID and annotation data from RAST. Sorted based on the highest total expression in RPKM.

File Name: Supplementary Data 4

Description: Gene families in *Anaeramoeba*. a, Global PANTHER (PTHR) families count by taxon. **b,** Homologous proteins by PANTHER (PTHR) family and taxon. **c,** Homologous proteins counts by PANTHER (PTHR) family and taxon. **d,** Taxon-specific homologous proteins by PANTHER (PTHR) family. **e,** *Anaeramoeba*-specific homologous proteins by PANTHER (PTHR) family. **f,** Relative contractions and expansions among different groups. **g,** Log2 fold change (log2 FC) by PANTHER (PTHR) family for *Anaeramoeba* with respect to other Eukaryotes. **h,** Log2 fold change (log2FC) by PANTHER (PTHR) family

for *A. flamelloides* relative to *A. ignava*. **i,** Top 50 family expansions in *Anaeramoeba* respect to other eukaryotes-group. **j,** Validated orthologous ribosomal proteins accessions by taxon. **k,** Counts of validated orthologous Ribosomal proteins by taxon. **l,** Orthologous assignments for DNA replication, repair and Meiosis systems.

File Name: Supplementary Data 5

Description: LGT statistics. a, Global numbers. **b,** LGTs targeted to hydrogenosome. **b,** Most represented Panther annotation. **c,** LGTs with >60% identity to Bacteria, Viruses or Archaea in NCBI nr. **d,** Acquisitions of the same ortholog. **e,** Host-symbiont interaction candidate proteins.

File Name: Supplementary Data 6

Description: Summary tables of membrane-trafficking components in *Anaeramoeba* a, Coulson-plots. **b,** HOPS-CORVET complex components. **c,** Rab-Orthofinder. **d,** TBC-Orthofinder.

File Name: Supplementary Data 7

Description: Anti-SMASH predictions for *Anaeramoeba* predicted proteomes.

File Name: Supplementary Data 8

Description: Sequencing libraries. Accession codes and description of sequencing datasets generated in the study.

File Name: Supplementary Data 9

Description: FISH probes. Oligonucleotide probes and hybridization conditions used in fluorescence *in situ* hybridization experiments.

File Name: Supplementary Data 10

Description: Defense systems in Desulfobacteraceae. PADLOC [67] was used to search the genomes of diverse free-living Desulfobacteraceae and symbionts of *Anaeramoeba*. MAG genomes are marked in gold.

File Name: Supplementary Data 11

Description: Amino acid pathways and unique proteins in the symbionts a, GapMind predictions of amino acid biosynthesis enzymes in *Anaeramoeba* symbionts and *Desulfobacula toluolica* Tol2. **b,** Unique proteins in pair-wise comparison of Sym_BUSS2 (A) and Sym_SCH1 (B). **c,** Unique proteins in pair-wise comparison of Sym_BUSS2 (A) and Sym_SCH1 (B). **d,** Unique proteins in pair-wise comparison of Sym_SCH1 (A) and Desulfobacter sp. BMAN (B). **e,** Unique proteins in pair-wise comparison of Sym_SCH1 (A) and Sym_BMAN (B). **f,** Unique proteins in pair-wise comparison of Sym_BUSS2 (A) and

Sym_BMAN (B). **g,** Unique proteins in pair-wise comparison of Sym_BUSS2 (A) and Sym_BMAN (B).

File Name: Supplementary Data 12

Description: Symbiont DNA repair. a, Strain vs strain comparison of DNA repair associated proteins. **b,** Presence / absence table of DNA repair proteins in selected Desulfobacteraceae.
