# Supplementary material for: A unique symbiosome in an anaerobic single-celled eukaryote
Source: Nat Commun. 2024 Nov 9;15:9726. doi: 10.1038/s41467-024-54102-7 (PMC11550330; doi:10.1038/s41467-024-54102-7)
Supplement: Supplementary file 17 — Reporting Summary [file 41467_2024_54102_MOESM17_ESM.pdf]

nature portfolio

Corresponding author(s): Jon Jerlström-Hultqvist, Andrew J. Roger

Last updated by author(s): Oct 9, 2024

Reporting Summary

Nature Portfolio wishes to improve the reproducibility of the work that we publish. This form provides structure for consistency and transparency in reporting. For further information on Nature Portfolio policies, see our Editorial Policies and the Editorial Policy Checklist.

Statistics

For all statistical analyses, confirm that the following items are present in the figure legend, table legend, main text, or Methods section.

n/a	Confirmed	
☒	☐	The exact sample size (*n*) for each experimental group/condition, given as a discrete number and unit of measurement
☒	☐	A statement on whether measurements were taken from distinct samples or whether the same sample was measured repeatedly
☒	☐	The statistical test(s) used AND whether they are one- or two-sided<br>*Only common tests should be described solely by name; describe more complex techniques in the Methods section.*
☒	☐	A description of all covariates tested
☒	☐	A description of any assumptions or corrections, such as tests of normality and adjustment for multiple comparisons
☒	☐	A full description of the statistical parameters including central tendency (e.g. means) or other basic estimates (e.g. regression coefficient) AND variation (e.g. standard deviation) or associated estimates of uncertainty (e.g. confidence intervals)
☒	☐	For null hypothesis testing, the test statistic (e.g. *F*, *t*, *r*) with confidence intervals, effect sizes, degrees of freedom and *P* value noted<br>*Give P values as exact values whenever suitable.*
☒	☐	For Bayesian analysis, information on the choice of priors and Markov chain Monte Carlo settings
☒	☐	For hierarchical and complex designs, identification of the appropriate level for tests and full reporting of outcomes
☒	☐	Estimates of effect sizes (e.g. Cohen's *d*, Pearson's *r*), indicating how they were calculated

Our web collection on statistics for biologists contains articles on many of the points above.

Software and code

Policy information about availability of computer code

Data collection	Oxford Nanopore Minion data was collected using the MinKNOW control software.<br>For FIB-SEM, volume acquisition was automated using Auto Slice and View 4 software provided with the Scios dual-beam instrument (Thermo Fischer Scientific)
Data analysis	Genome sequencing and assembly:<br>Illumina sequencing reads were quality checked with FastQC v.0.11.5.<br>Oxford Nanopore reads were basecalled using Guppy v2.3.5 (Oxford Nanopore).<br>Fastq reads were trimmed using Porechop v0.2.3_seqan2.1.1 .<br>Whole genome assembly for A. ignava was performed with ABruijn (v1.0), corrected by Racon (v0.5), then Nanopolish v0.8.4 followed by two rounds of Pilon (v1.22) polishing.<br>For A. flamelloides, Fye (v2.4) was used for assembly which was then corrected by four rounds of Racon (v1.4.13) and polished using Medaka v0.6.2. Final assemblies were generated using five rounds of Pilon (v1.23).<br>Hybrid assembly of symbiont genomes used Unicycler and ANI values were calculated using OAT.<br><br>GC% was calculated by coungc.sh in the BBMap package v38.20<br><br>Read-mapping<br>Read mapping for Illumina DNA data utilized bowtie2 (v2.3.1)<br>For Nanopore read mapping, minimap2 (v2.10-r761) was used.<br>For RNAseq read mapping Hisat2 v2.1.0 was used

Annotation of symbiont genomes used Rapid Annotation using Subsystem Technology v2.0, with insertion sequences predicted with ISsaga v2.0 and pseudogene candidates identified using Pseudofinder v0.11. Synteny was assessed using Sibelia v3.0.7

16S rDNA amplicon analysis used MicrobiomeHelper

Phylogenomic analyses: i) selection of genomes and marker genes used phyloSkeleton v1.1, ii) alignments were conducted with MAFFT-linsi v7.458 and trimmed with BMGE v1.12, iii) phylogenetic trees were inferred using IQTree v2.0.3

Gene family expansions/contraction analysis utilized HMMER v3.1b2 and the Panther Score tool. For membrane trafficking analyses orthologous clustering used OrthoFinder v2.0.0 and alignments were conducted with MAFFT-L-INS-i v7.458 and trimmed using trimAl v1.4.rev15. Phylogenetic trees were estimated using IQ-TREE v1.6.8

Lateral gene transfer analyses used Orthofinder v2.5.4 and alignments were created with MAFFT v7.310 L-INS-i, with BMGE v1.0 used for trimming. Phylogenetic trees were estimated with FastTree v1.0.1 for screening, followed by IQ-TREE v.1.5.5 for in-depth analysis.

Symbiont 16S rDNA sequences were extracted from the RAST annotations and aligned by MAFFT v7

Fluorescence in situ hybridization and immunofluorescence data, image analysis used Zeiss ZEN v3.1 or BioImageXD

FIB-SEM volumes were registered and processed using ImagJ plugins SIFT and Multistackreg. Segmentation of volumes used Microscopy Image Browser v2.84 followed by rendering with ORS Dragonfly v2022.2.0.1399.

For manuscripts utilizing custom algorithms or software that are central to the research but not yet described in published literature, software must be made available to editors and reviewers. We strongly encourage code deposition in a community repository (e.g. GitHub). See the Nature Portfolio guidelines for submitting code & software for further information.

Data

Policy information about availability of data

All manuscripts must include a data availability statement. This statement should provide the following information, where applicable:

- Accession codes, unique identifiers, or web links for publicly available datasets
- A description of any restrictions on data availability
- For clinical datasets or third party data, please ensure that the statement adheres to our policy

Sequencing reads and the annotated genomes of A. flamelloides BUSSELTON2, A. flamelloides SCHOONER1, A. ignava BMAN, Sym_BUSS2, Sym_SCH1 and Sym_BMAN were deposited to NCBI under the BioProject number PRJNA634776.

FIB-SEM data is available for reviewers in Figshare: https://figshare.com/s/00a5b9710d961c94f2fd.

Research involving human participants, their data, or biological material

Policy information about studies with human participants or human data. See also policy information about sex, gender (identity/presentation), and sexual orientation and race, ethnicity and racism.

Reporting on sex and gender	n/a
Reporting on race, ethnicity, or other socially relevant groupings	n/a
Population characteristics	n/a
Recruitment	n/a
Ethics oversight	n/a

Note that full information on the approval of the study protocol must also be provided in the manuscript.

Field-specific reporting

Please select the one below that is the best fit for your research. If you are not sure, read the appropriate sections before making your selection.

☐ Life sciences ☐ Behavioural & social sciences ☒ Ecological, evolutionary & environmental sciences

For a reference copy of the document with all sections, see nature.com/documents/nr-reporting-summary-flat.pdf

Ecological, evolutionary & environmental sciences study design

All studies must disclose on these points even when the disclosure is negative.

Study description	The study involved complete genome sequencing of 3 strains of Anaeramoeba (two strains of A. flamelloides and one strain of A. ignava). Because all the associated microbiota were also subjected to sequencing, complete genome sequences of the associated Desulfobacter symbionts were also generated. Full whole genome gene prediction/annotation was completed and a large battery of analyses (described in the manuscript) were conducted to investigate gene content and evolution. Microscopy analyses established that the endosymbionts within the Anaeramoebae were Desulfobacters and electron microscopic tomography analyses reconstructed the 3D structure of the Anaeramoeba flamelloides cell, with a focus on the symbiosome and host hydrogenosomes. Extensive comparative analyses established likely metabolic interactions between hosts and symbionts and demonstrated evolutionary adaptations of the host to housing the symbionts.
Research sample	The research 'samples' are cultures of the 3 Anaeramoeba strains and associated microbiota whose genomes and transcriptomes were sequenced and which were examined using a variety of microscopy techniques.
Sampling strategy	This is a study involving comparative genomic analyses, trascriptomics, microscopy analyses and a variety of bioinformatic investigations. There was no 'sampling' involved.
Data collection	The primary data for this study include: i) whole genome sequences, ii) transcriptome sequences, iii) in situ fluorescence microscopy analyses gathered using advanced fluorescence and light microscopy and iv) several types of electron microscopy analyses.
Timing and spatial scale	There was no temporal or spatial 'scale' for the analyses since they were all conducted with cultured organisms within the laboratory.
Data exclusions	Data was not excluded from the study.
Reproducibility	For full genome sequencing and comparative analyses there is no need (or expectation by the relevant scientific community) that reproducibility is investigated. The methods are completely reproducible and generate completely reproducible DNA sequences. For the fluorescence in situ hybridization analyses triplicates were performed with one exception (Anaeramoeba ignava BMAN samples) which were done in duplicate (a third replicate could not be done because these difficult-to-maintain cultures died in all laboratories which maintain them).
Randomization	There are no analyses included that have allocation of individual samples into groups, so randomization is not relevant to this work.
Blinding	Blinding is not relevant to this work as it involves genomic studies of cultured organisms.

Did the study involve field work? ☐ Yes ☒ No

Reporting for specific materials, systems and methods

We require information from authors about some types of materials, experimental systems and methods used in many studies. Here, indicate whether each material, system or method listed is relevant to your study. If you are not sure if a list item applies to your research, read the appropriate section before selecting a response.

Materials & experimental systems

n/a	Involved in the study	
☐	☒	Antibodies
☒	☐	Eukaryotic cell lines
☒	☐	Palaeontology and archaeology
☐	☒	Animals and other organisms
☒	☐	Clinical data
☒	☐	Dual use research of concern
☒	☐	Plants

Methods

n/a	Involved in the study	
☒	☐	ChIP-seq
☒	☐	Flow cytometry
☒	☐	MRI-based neuroimaging

Antibodies

Antibodies used	TAT1 antibody: (mouse x mouse hybridoma): cross reacts with alpha tubulin.
Validation	The antibody is a monoclonal antibody raised against developed by immunizing mice with extracted T. brucei brucei cytoskeleton and fusing the mouse spleen cells with the myeloma Sp2-0/Ag. This cell line has been cloned many times and the mixed subclass is now typical of the cloned line. The antibody detects alpha tubulin. TAT1 was first produced by Woods et al. (1989) J Cell Sci 93 (3): 491–500 and in that paper it's activity against alpha tubulin in T. brucei and mammalian brain is demonstrated by western blotting (see Fig. 4). It also cross-reacted with microtubules in immunofluorescence and immunogold labelling of T. brucei cells (Figs. 2 and 3) and in immunofluorescence labelling of Vero cells (African green monkey kidney cell line) (Fig. 5). Specificity for Physarum alpha tubulin

was also demonstrated in that paper.

Eukaryotic cell lines

Policy information about cell lines and Sex and Gender in Research

Cell line source(s)	n/a
Authentication	n/a
Mycoplasma contamination	n/a
Commonly misidentified lines (See ICLAC register)	n/a

Palaeontology and Archaeology

Specimen provenance	n/a
Specimen deposition	n/a
Dating methods	n/a

☐ Tick this box to confirm that the raw and calibrated dates are available in the paper or in Supplementary Information.

Ethics oversight	n/a

Note that full information on the approval of the study protocol must also be provided in the manuscript.

Animals and other research organisms

Policy information about studies involving animals; ARRIVE guidelines recommended for reporting animal research, and Sex and Gender in Research

Laboratory animals	The organisms are not animals, they are anaerobic protists in stable culture. Three isolates were studied: Two strains of Anaeramoeba flamelloides (BUSSELTON and SCHOONER strains) and Anaeramoeba ignava
Wild animals	none
Reporting on sex	These are microbial organisms and it is unknown whether or not they undergo a sexual cycle.
Field-collected samples	There were no field-collected samples
Ethics oversight	Because these are unicellular organisms (microbes), no ethical oversight is required.

Note that full information on the approval of the study protocol must also be provided in the manuscript.

Clinical data

Policy information about clinical studies
All manuscripts should comply with the ICMJE guidelines for publication of clinical research and a completed CONSORT checklist must be included with all submissions.

Clinical trial registration	n/a
Study protocol	n/a
Data collection	n/a
Outcomes	n/a

Dual use research of concern

Policy information about dual use research of concern

Hazards

Could the accidental, deliberate or reckless misuse of agents or technologies generated in the work, or the application of information presented in the manuscript, pose a threat to:

No	Yes	
☒	☐	Public health
☒	☐	National security
☒	☐	Crops and/or livestock
☒	☐	Ecosystems
☒	☐	Any other significant area

Experiments of concern

Does the work involve any of these experiments of concern:

No	Yes	
☒	☐	Demonstrate how to render a vaccine ineffective
☒	☐	Confer resistance to therapeutically useful antibiotics or antiviral agents
☒	☐	Enhance the virulence of a pathogen or render a nonpathogen virulent
☒	☐	Increase transmissibility of a pathogen
☒	☐	Alter the host range of a pathogen
☒	☐	Enable evasion of diagnostic/detection modalities
☒	☐	Enable the weaponization of a biological agent or toxin
☒	☐	Any other potentially harmful combination of experiments and agents

Plants

Seed stocks	n/a
Novel plant genotypes	n/a
Authentication	n/a

ChIP-seq

Data deposition

☐ Confirm that both raw and final processed data have been deposited in a public database such as GEO.

☐ Confirm that you have deposited or provided access to graph files (e.g. BED files) for the called peaks.

Data access links<br>*May remain private before publication.*	n/a
Files in database submission	n/a
Genome browser session<br>(e.g. UCSC)	n/a

Methodology

Replicates	n/a
Sequencing depth	n/a
Antibodies	n/a
Peak calling parameters	n/a
Data quality	n/a
Software	n/a

Flow Cytometry

Plots

Confirm that:

- [ ] The axis labels state the marker and fluorochrome used (e.g. CD4-FITC).
- [ ] The axis scales are clearly visible. Include numbers along axes only for bottom left plot of group (a 'group' is an analysis of identical markers).
- [ ] All plots are contour plots with outliers or pseudocolor plots.
- [ ] A numerical value for number of cells or percentage (with statistics) is provided.

Methodology

Sample preparation	n/a
Instrument	n/a
Software	n/a
Cell population abundance	n/a
Gating strategy	n/a

- [ ] Tick this box to confirm that a figure exemplifying the gating strategy is provided in the Supplementary Information.

Magnetic resonance imaging

Experimental design

Design type	n/a
Design specifications	n/a
Behavioral performance measures	n/a

Acquisition

Imaging type(s)	n/a
Field strength	n/a
Sequence & imaging parameters	n/a
Area of acquisition	n/a

Diffusion MRI ☐ Used ☐ Not used

Preprocessing

Preprocessing software	n/a
Normalization	n/a
Normalization template	n/a
Noise and artifact removal	n/a
Volume censoring	n/a

Statistical modeling & inference

Model type and settings	n/a
Effect(s) tested	n/a

Specify type of analysis: ☐ Whole brain ☐ ROI-based ☐ Both

Statistic type for inference

(See Eklund et al. 2016)

n/a

Correction

n/a

Models & analysis

n/a	Involved in the study
☒	☐ Functional and/or effective connectivity
☒	☐ Graph analysis
☒	☐ Multivariate modeling or predictive analysis

Functional and/or effective connectivity

Report the measures of dependence used and the model details (e.g. Pearson correlation, partial correlation, mutual information).

Graph analysis

Report the dependent variable and connectivity measure, specifying weighted graph or binarized graph, subject- or group-level, and the global and/or node summaries used (e.g. clustering coefficient, efficiency, etc.).

Multivariate modeling and predictive analysis

Specify independent variables, features extraction and dimension reduction, model, training and evaluation metrics.
